# A Standardized Brain Molecular Atlas: A Resource for Systems Modeling and Simulation

**DOI:** 10.3389/fnmol.2021.604559

**Published:** 2021-11-10

**Authors:** Polina Shichkova, Jay S. Coggan, Henry Markram, Daniel Keller

**Affiliations:** ^1^Blue Brain Project, École Polytechnique Fédérale de Lausanne, Geneva, Switzerland; ^2^Laboratory of Neural Microcircuitry, Brain Mind Institute, École Polytechnique Fédérale de Lausanne, Lausanne, Switzerland

**Keywords:** molecular concentrations, neuroproteomics, quantitative resource, data integration, mouse brain, systems modelling and simulation, differential protein expression, meta-analysis

## Abstract

Accurate molecular concentrations are essential for reliable analyses of biochemical networks and the creation of predictive models for molecular and systems biology, yet protein and metabolite concentrations used in such models are often poorly constrained or irreproducible. Challenges of using data from different sources include conflicts in nomenclature and units, as well as discrepancies in experimental procedures, data processing and implementation of the model. To obtain a consistent estimate of protein and metabolite levels, we integrated and normalized data from a large variety of sources to calculate Adjusted Molecular Concentrations. We found a high degree of reproducibility and consistency of many molecular species across brain regions and cell types, consistent with tight homeostatic regulation. We demonstrated the value of this normalization with differential protein expression analyses related to neurodegenerative diseases, brain regions and cell types. We also used the results in proof-of-concept simulations of brain energy metabolism. The standardized Brain Molecular Atlas overcomes the obstacles of missing or inconsistent data to support systems biology research and is provided as a resource for biomolecular modeling.

## Introduction

A deeper understanding of the functions of biomolecular networks requires more accurate and reproducible proteomic and metabolomic concentration profiles. Decades of accumulated data have fed this demand, but the disparity of experimental methods and apparent discrepancies in results have hampered progress and many biological conditions still lack quantitative proteomic and metabolomic characterization.

Studies that reconstruct and simulate molecular systems usually rely on knowledge from various sources but there are not many studies which provide extensive comparison of newly generated data to existing independent sources or integrate and re-analyze data of different provenance (Ho et al., 2018; McKenzie et al., 2018). Consequently, modeling faces the challenge of integrating non-homogeneous data from different experimental protocols, species, ages, cell types and even tissues, as well as measured levels of detail. In fact, systematic errors arising from various experimental procedures can affect the quality of the data, models and simulations, leading to inconsistencies and debates about the biology of the processes and interpretation of observations. An integrated resource is therefore desirable to enhance multiscale analysis of a system and assist subsequent experimental design.

We sought to estimate concentrations of proteins and metabolites in the brain from a multitude of studies, with the goal of providing data of sufficient quality for use in simulations and as a reference for comparison in future studies. The integrated data give a quantitative overview across different brain regions, cell types, organelles, species, ages and conditions, and can serve as a navigator for brain researchers to find new targets for their studies.

There are significant obstacles in obtaining comparable multiscale absolute quantification protein data due to confounding variables resulting from different experimental subjects and approaches. Proteomic quantification methods usually require preselection of specific protein targets to be measured because of logistical issues in experimental setup (Remes et al., 2020). Even though the literature describes many comprehensive transcriptomics data sets (Cahoy et al., 2008; Tasic et al., 2016; McKenzie et al., 2018; Zeisel et al., 2018), due to regulatory mechanisms and turnover, protein levels are not always well-correlated with gene expression (Vogel et al., 2010; Schwanhäusser et al., 2011; Edfors et al., 2016; Silva and Vogel, 2016; Li et al., 2017; Mandad et al., 2018; Eraslan et al., 2019). This complicates the use of transcriptomics data in biochemical simulations. Nonetheless, gene expression can help infer protein level estimates, when other measurements are not available.

The final product of the pipeline developed in this work is a normalized molecular concentration database called the Brain Molecular Atlas (also referred to as Molecular Atlas). We found a high degree of data reproducibility across studies, as well as consistency among brain regions. We demonstrate its potential for creating more accurate representations of biomolecular systems that are simulation-ready.

As a case study, we present an analysis of molecular profiles associated with Alzheimer’s (AD) and experimental autoimmune encephalomyelitis (EAE) diseases. In a second demonstration, we apply the Molecular Atlas to the examination of energy metabolism-related processes. Although studied for decades, this field is in need of improved detailed models as there are ongoing debates about energy metabolism mechanisms and even which homeostatic processes are mediated by well-known pathways (Baeza-Lehnert et al., 2019; Gerkau et al., 2019).

## Materials and Methods

**FIGURE 1:**
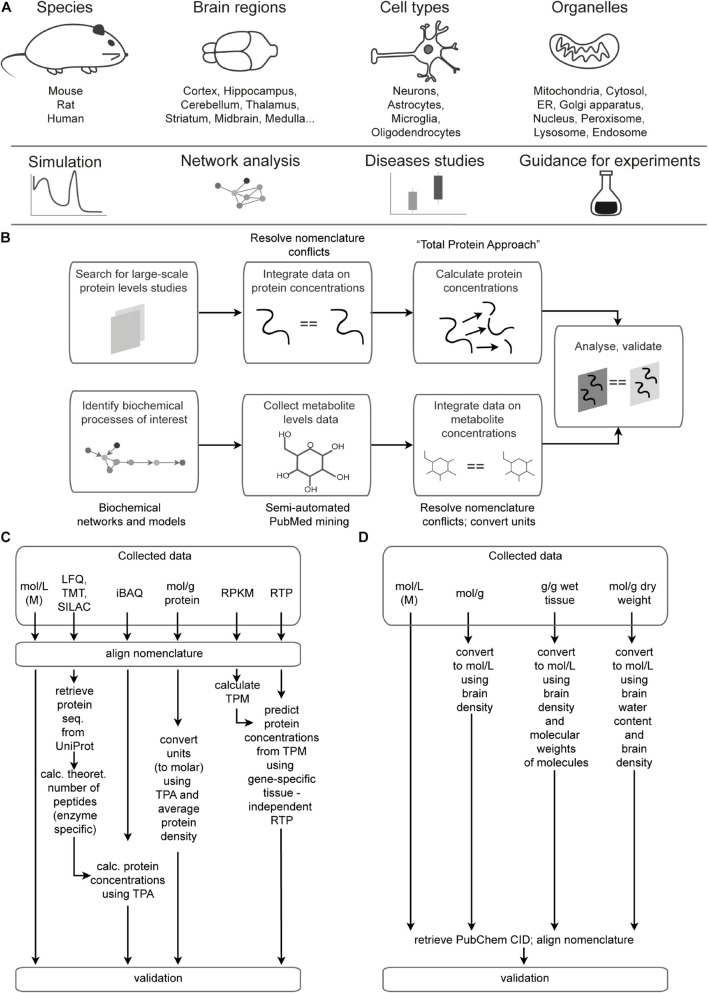
Data scope overview and integration pipeline. **(A)** Scope and potential applications of data integrated in the Brain Molecular Atlas. **(B)** General pipeline for the Brain Molecular Atlas data integration. **(C,D)** Detailed pipeline for calculating protein **(C)** and metabolite **(D)** concentrations. Abbreviations: ER = endoplasmic reticulum, LFQ = label-free quantification; TMT = tandem mass tag; SILAC = stable isotope labeling by/with amino acids in cell culture; iBAQ = intensity-based absolute quantification; RPKM = reads per kilobase million; TPM = transcripts per kilobase million; RTP = RNA to protein ratio estimator; CID = compound ID number.

There are two sections of the Brain Molecular Atlas, one corresponding to protein and the other to metabolite concentrations. The scope of the data for the Brain Molecular Atlas, as well as data integration procedures are shown in Figure 1. The data integration pipeline for each section consists of the following phases: data mining, nomenclature alignment, and concentrations estimation, which involve calculations of molar concentrations and subsequent normalization, followed by validation.

Primarily mouse data were supplemented with rat and human data to get higher coverage of different experimental conditions, ages, brain regions and organelles. These species were chosen due to their importance for a wide range of neuroscience studies. Most of the data describe healthy states. Additional data on AD and EAE were collected for evaluation of the discriminative power of the estimated molecular concentrations procedure and can be accessed in the “condition” column in Supplementary Data Sheets 1-4.

The code is available from the repositories https://github.com/BlueBrain/MADIP (for the data processing) and https://github.com/BlueBrain/BrainMolecularAtlas (for generating the figures) to support transparency, reproducibility and analysis of the new data. A detailed description of the data integration pipeline can be found in the Supplementary Presentation.

### Data Mining

The first step of the data integration pipeline is data acquisition. Although there are many initiatives to automate data collection (Breckels et al., 2016), most of them are applicable only for specific domains, types of data (Wang et al., 2012) or particular organisms (Wilhelm et al., 2014). Our strategy for data collection consisted of several steps.

#### Proteins

We manually searched for large-scale mass-spectrometry based studies in PubMed and ProteomeXchange repositories (Deutsch et al., 2019). Decisions on whether to include or exclude brain protein quantification data sources for the selected species of interest (mouse, rat, human) were made based on the following criteria: (1) absolute versus relative quantities reported, where we only chose the former, because the absolute quantification is particularly important for the research on the modeling and simulation of the molecular processes (for instance, this resulted in the exclusion of valuable data from Yu et al. (2020) reporting relative scaled abundances); (2) reported protein levels are LFQ, iBAQ, TMT-based abundances or concentrations, because the desired common unit of molar concentrations could be obtained from these data. This culling process resulted in the inclusion of fewer than 5% of all papers that we initially considered. From the hundreds of studies primarily identified this way, we selected only 22 the most relevant studies resulting in 25 datasets for integration in our database (Geiger et al., 2013; Han et al., 2014; Sharma et al., 2015; Wiśniewski et al., 2015; Jean Beltran et al., 2016; Carlyle et al., 2017; Hosp et al., 2017; Itzhak et al., 2017; Chuang et al., 2018; Duda et al., 2018; Fornasiero et al., 2018; Hamezah et al., 2018, 2019; Krogager et al., 2018; Zhu et al., 2018; Davis et al., 2019; Fecher et al., 2019; Guergues et al., 2019; Hasan et al., 2019; McKetney et al., 2019; Bai et al., 2020; Kjell et al., 2020). Additional information on each of those is summarized in the Supplementary Table 1.

#### Metabolites

Metabolite concentrations had to be collected from many different sources. Due to experimental complications, metabolic studies usually provide data for only subsets of around 10-30 target molecules. Automation of data mining is therefore required to achieve higher coverage of the metabolite landscape of the NGV system. We identified metabolic pathways of key interest as follows: glycolysis, glycogenolysis, pentose phosphate pathway, the tricarboxylic acid (TCA) cycle, the electron transport chain of oxidative phosphorylation, and the glutamate-glutamine cycle. Further, we collected the biochemical reactions, metabolites and enzymes which constitute these pathways. We then generated PubMed queries (the list of main metabolites and energy-metabolism related enzymes combined with the list of cell types, subcellular locations and methods) to get more precise matches for kinetics of processes and concentrations of molecules related to brain and the species of interest (mouse, rat, or human). An example of a query is: “(mouse OR rat OR human) AND (brain OR glia OR astrocyte OR neuron) AND hexokinase AND (quanti^∗^ OR measur^∗^ OR estimat^∗^) AND ((concentration NOT attention) OR level).” We performed automatic PubMed searches and text mining using R package Adjutant (Crisan et al., 2019), which resulted in 5405 hits. We next performed text corpus generation (bag-of-words), dimensionality reduction (tSNE) and unsupervised clustering (HDBSCAN) with the same R package to support navigation of that large number of hits. Representative PubMed mining results are shown in Supplementary Figure 1, where cosine similarity of clusters is a measure for associations of topics. The automated data mining can be reproduced using the code accompanying this paper to obtain the list of initial search hits, which are also available (with corresponding queries) as Supplementary Table 3. Then we manually analyzed papers for the topics of highest interest, and combined this data with the information from the set of large-scale studies, databases and other studies which were found by manual search. This resulted in selection of data from only 41 sources (references are available from the Supplementary Table 1 and Supplementary Data Sheet 5). The low percentage of included hits is mainly due to the fact that the automatically found publications frequently featured quantitative data and concentrations as part of the methods section rather than measured results.

### Nomenclature Alignment

#### Proteins

The multitude of gene and protein identifiers dictates the need for performing nomenclature alignment to resolve naming inconsistencies. Most studies reported both gene names and UniProt accession numbers (The UniProt Consortium, 2017), but a few of the analyzed studies gave only one or the other. In some cases, UniProt identifiers become obsolete and require mapping to the current version. Moreover, it is very typical for proteomics studies to report multiple UniProt numbers per single entry, as provided by proteomic mass-spectrometry annotation algorithms. Synonymous gene names are equally problematic. Orthologous gene names from different species are another challenging problem in data integration. Since nomenclature misalignment complicates the discoverability and comparability of the data, we generated a consistent list of genes and proteins with the mouse gene nomenclature as a reference and queried UniProt (The UniProt Consortium, 2017) and Mouse Genome Database (Bult et al., 2019) to resolve the nomenclature conflicts.

To avoid introducing additional gene and protein identifiers, we sought the most common name for every gene for which we had corresponding protein concentration data (and most common UniProt identifiers). In uncertain situations, we preferred mouse identifiers, because the mouse is the main genus of interest for us. In much of the raw data, multiple synonymous gene names were given per data entry. For example, gene names SPRYD7 and 6330409N04RIK (SPRY domain-containing protein 7) are listed in Geiger et al. (2013), Wiśniewski et al. (2015) and cultured cell data from Sharma et al. (2015), while 11 other datasets report it by the name SPRYD7. Interestingly, the isolated cell data from Sharma et al. (2015) is among the latest. This can be explained by automated annotation procedures used in proteomics pipelines. Moreover, in one of the most recent studies (Hasan et al., 2019) only UniProt IDs are given, and the protein of corresponding gene is reported under the UniProt identificator Q3TFQ1.

We used synonymous gene names to build a graph in which gene names are the vertices. Gene names that are listed together for the same entry are connected by edges. When two or more data entries share one or a few common gene names and possibly some other gene names, and there are no other entries that have any of these gene names, these two subgraphs will form a connected component of the graph. We consider nodes of every connected component as potential synonyms. In the same way, the graph of synonyms was built using the UniProt database entries for mouse, rat and human, listing multiple gene names and UniProt protein accession identifiers. For every connected component in the names graph, we assigned the most frequent gene name. This produced a dictionary with gene name mapping. To identify mis-mapping due to the observed ambiguities in gene names given in the source data, we cross validated the original gene names and their matches, with the corresponding UniProt accession numbers. We identified several cases of non-synonymous gene names reported for the same entry. UniProt-derived synonyms were used for this step. Some nomenclature conflicts found by manual checks were resolved with the use of the Mouse Genome Database and UniProt, the most common gene names were kept as final identifiers in the integrated Molecular Atlas. In a similar manner, we performed the nomenclature alignment for UniProt accession identifiers reported in the raw data. More details can be found in Supplementary Presentation and the commented source code.

For studies which contained only genes or only UniProt identifiers, we queried the UniProt database to acquire missing information. We consider gene names as the main identifiers of the Brain Molecular Atlas, even though this leads to the merging of information associated with different protein isoforms. UniProt accession identifiers are available for reference and transparency in Supplementary Data Sheets 1-4.

#### Metabolites

We utilized the PubChem compound Identifier (Kim et al., 2019) to resolve nomenclature inconsistencies. In some cases, we also had to do manual data curation to resolve ambiguity in names of molecules.

### Concentration Estimations

#### Proteins

We applied experimental data- and unit-dependent processing procedures as outlined in Figures 1B-D and as detailed below. In addition to concentrations reported in mol/g protein and molar units, we included protein concentration estimates which rely on the recent high-throughput labeling [tandem mass tag (TMT) and stable isotope labeling by amino acids in cell culture (SILAC)] or label-free mass-spectrometry based proteomics studies. These strategies are untargeted, involving both identification and quantification of proteins. Depending on the methods used by each study, protein levels are reported as TMT-, SILAC-, LFQ-intensities, intensity-based absolute quantification (iBAQ) values, concentrations in mol/g protein and in molar units. TMT is a chemical labeling approach (Thompson et al., 2003), that has high sensitivity and allows detection of proteins, which are present at the low abundances. SILAC is an efficient metabolic labeling technology (Ong et al., 2002) involving the use of heavy isotopes of amino acids being incorporated into cell proteins. The techniques which do not require labeling steps are referred as label-free (LFQ) (Chelius and Bondarenko, 2002; Bantscheff et al., 2007; Ning et al., 2012; Cox et al., 2014; Ankney et al., 2018). They are widely used, rapid and relatively inexpensive. On the downside, LFQ experiments show high risk of bias and require tight control. Various algorithms are available to analyze the data from label-free studies and quantify protein levels. One of the protein abundance measures is called iBAQ, and it is calculated as summed intensities of peptides of a particular protein, divided by the number of peptides that theoretically can be produced from this protein (Schwanhäusser et al., 2011).

Protein concentrations can be estimated from the mass-spectrometry data even if it does not feature spike-in standards with the widely used “proteomic ruler” approach or total protein mass approach (Wiśniewski et al., 2014) in cases when there is not enough data on histone levels. While there are some software solutions to determine protein concentrations in this regard (Tyanova et al., 2016), we implemented in Python the main principles and formulas of the total protein mass approach in order to combine concentration calculations with other analyses in the same pipeline. We adapted this approach to calculate concentrations using mass-spectrometry based data mostly with normalization based on the number of theoretical peptides calculated from UniProt protein sequences by enzyme specificity for particular amino acids. Molecular weights of proteins were taken from the original data or queried from UniProt in cases when they were not available from the source data. UniProt protein sequences and molecular weights were queried with the use of methods reported in the literature (Cokelaer et al., 2013; Tange, 2020). Molecular weights of proteins were used as part of scaling calculated protein concentrations. For LFQ, TMT, SILAC data, we used the number of theoretical peptides as an additional correction. Within iBAQ data, signals were already scaled to the number of peptides. The data from Wiśniewski et al. (2015), Duda et al. (2018) were reported in the units of concentrations, which were calculated by the sources analogously with adaptation of the total protein approach.

For concentration estimations based on the data from Geiger et al. (2013), Han et al. (2014), Sharma et al. (2015), Carlyle et al. (2017), Hamezah et al. (2018), Hamezah et al. (2019), Krogager et al. (2018), Zhu et al. (2018), Fecher et al. (2019), Guergues et al. (2019), McKetney et al. (2019), Kjell et al. (2020), we programmatically obtained numbers of theoretical peptides by enzyme specificity for particular amino acids for every experiment using protein sequences from UniProt. Peptide counts were used as detectability scaling. Concentrations were also estimated for the data from Jean Beltran et al. (2016), Hosp et al. (2017), Chuang et al. (2018), Fornasiero et al. (2018), Davis et al. (2019). The procedure here was very similar to the LFQ data cases, but we did not do the scaling by number of theoretical peptides working with iBAQ data which by definition has this type of normalization.

Formulas summarizing the calculation of concentrations are based on Wiśniewski et al. (2014) and given by eqs. (1-4). First, there are several protein-specific factors which affect protein detectability by mass-spectrometry based methods. One of these factors is the number of peptides which can be formed by any given protein cleaved by the enzyme or enzymes used in proteomic experiments. For tryptic peptides, we split sequences by arginine and lysine into peptides and counted those that had a length from 6 to 29 amino acids. The same logic was applied to count theoretical peptides in experiments with the lysC enzyme. Another factor which explains variable accuracy in protein detectability is the molecular weight of the protein. Next, according to the same reference (Wiśniewski et al., 2014), total cellular protein concentration was considered to be 200 g/L, and protein amount per cell was taken as 200 pg. Even though these are commonly used estimates, the analysis would benefit by replacing them with more cell type and tissue specific numbers. However, this data is not always available. Further, protein copy number can be estimated using mass-spectrometry signals, the Avogadro constant and parameters described above. Likewise, total cell volume and protein molar concentration can be further derived as shown by eqs. (3, 4).


(1) $$\begin{array}{c} mwWeightNormSumIntens=\\ sum\left(\frac{LFQ}{detectabilityFactorTheorPep}*molWeight\right) \end{array}$$

(2) $$\begin{array}{c} copyNumber=\frac{LFQ}{detectabilityFactorTheorPep}*\\ \left(protPerCell*Avogadro/mwWeightNormSumIntens\right) \end{array}$$

(3) $$totalVolume=\left(\frac{sum\left(copyNumber*\frac{molWeight}{Avogadro}\right)}{totalCellProtConc}\right)$$

(4) $$concentration=\frac{copyNumber}{totalVolume}/Avogadro$$

where the variables are as follows: *Avogadro* - Avogadro constant; *concentration* - molar protein concentration; *copyNumber* - protein copy number; *detectabilityFactorTheorPep* - experimental detectability number (number of theoretical peptides); *LFQ* - value from label-free quantification; *molWeight* - protein molecular weight; *mwWeightNormSumIntens* - weighted for detectability normalized by molecular weight summed LFQ values (see the formula above); *protPerCell* - protein amount per cell; *sum* - summation; *totalCellProtConc* - total cellular protein concentration; *totalVolume* - total cell volume.

We then added data from Itzhak et al. (2017), which is provided in molar units, so we did not need to make estimates. Concentrations were scaled to μM and median normalized using housekeeping proteins data as described in Section “Results.”

The next step of the data processing pipeline was to normalize estimated protein concentrations by the median concentrations of housekeeping proteins from healthy young to middle-aged mice (and mice cell lines). We did not include in our calculation the reference median value for normalization data from Fecher et al. (2019), because it reported only concentrations in mitochondria. However, this data was further normalized using the reference median value to make it comparable with other data. The list of housekeeping protein identifiers was obtained from the Housekeeping Transcript Atlas (Hounkpe et al., 2021). This approach allowed us to decrease the effect of factors that cannot be easily controlled in the experiments, such as sample preparation bias.

#### Metabolites

The second part of the Brain Molecular Atlas is composed of metabolite concentrations, which were semi-automatically collected from a variety of resources (Kauffman et al., 1969; Tsuboi et al., 1969; Gibson and Blass, 1976; Sølling, 1979; Anderson and Wright, 1980; Sabate et al., 1995; Pouwels and Frahm, 1998; Lust et al., 2003; Patel et al., 2004; Cruz et al., 2005; Cudalbu et al., 2005; Nakayama et al., 2005; Mogilevskaya et al., 2006; Shestov et al., 2007; Wishart et al., 2007, 2009, 2012, 2018; Metelkin et al., 2009; Kulak et al., 2010; Choi and Gruetter, 2012; Neves et al., 2012; Sugimoto et al., 2012; Zheng et al., 2012, 2016; Duarte and Gruetter, 2013; Palm et al., 2013; Kim et al., 2014; Lee et al., 2014; Wiebenga et al., 2014; Berndt et al., 2015; Jolivet et al., 2015; Chen J. et al., 2016; Chen W.W. et al., 2016; Robinson and Jackson, 2016; Schwarz and Blower, 2016; Tretter et al., 2016; Hertz and Rothman, 2017; McBean, 2017; Calvetti et al., 2018; De Feyter et al., 2018; Flanagan et al., 2018; Liu et al., 2018; Ronowska et al., 2018) that are listed with the metabolite levels in the Supplementary Data Sheet 5. Depending on the initial data type, appropriate transformations were applied to get molar concentrations (Figure 1D). For instance, we used a rat brain density value of 1.04 g/mL (DiResta et al., 1991) and molecular weights of metabolites when dealing with ‘ng/g wet tissue’ units. Brain water content was considered to be 80% for approximations (Keep et al., 2012) when working with data of ‘nmol/mg dry weight’. The calculations and data analyses were performed using Python and R programming languages as described in detail in the Supplementary Presentation.

### Validation Strategy

The search for validation data is particularly demanding. While it would be ideal to compare calculated concentrations to an independent set of studies measuring the concentrations of the large number of molecules by some other experimental techniques, to our knowledge such data is missing. Moreover, separating the datasets for validation would mean not using them for the database itself, decreasing its coverage and, subsequently, the statistical power of the analyses done using the database. For these reasons, we had to come up with a set of evaluations (strictly speaking, evaluations should not be called validations) which address the correctness of the different aspects of the database, such as:

(1) comparison of absolute levels for signaling protein concentrations to the study not used in our atlas (Milo et al., 2010), and evaluated total protein numbers per cell to the literature level (Milo, 2013);
(2) similarity and difference between various groups of proteins from different pairs of studies, brain regions, cell types; proteins of different functions and proteins from different locations;
(3) PubMed co-mentions of gene names with cell types in which the concentration of related protein was found as higher than compared to all other cell types (assuming that proteins with higher cell-type specificity measure are expected to have more co-mentions of their names with those cell types in PubMed search);
(4) functional analysis of overrepresented proteins across brain regions and cell;
(5) testing the discriminatory abilities of the calculated concentrations in the approach analogous to the differential protein expression.

### Comparison of Estimated Protein Concentrations to Literature

The aim of the first step in the assessment of generated concentrations and copy numbers was to compare them to the literature values. Estimated protein concentrations were compared to the publicly available data from Itzhak et al. (2017), which was also included in the Molecular Atlas and partially used for the normalization (see more details in Results). Since this study applied a very similar approach to evaluate molar concentrations, given comparisons can only control for possible problems in our adaptation of the total protein approach (Wiśniewski et al., 2014).

Total protein copy numbers per cell were compared to one different study (Milo, 2013). Concentrations of signaling proteins were compared to literature data from Harvard BioNumbers (Milo et al., 2010). Statistical analysis was performed in Python with the use of Scipy (Virtanen et al., 2020) and Scikit-posthocs (Terpilowski, 2019).

### Statistical Analysis for Multiple Comparisons of Data Sets

The normalization procedure relied on the concentrations of the housekeeping genes, so we next assessed equality of medians of the full data sets to see if they were in agreement. To compare normalized protein and peptide concentrations from different studies we calculated Holm adjusted *p*-Values from the Conover *post hoc* test applied after the Kruskal-Wallis Test rejection. We have chosen the Kruskal-Wallis Test to examine the equality of medians among multiple independent samples of different sizes, because it is a distribution-free test, for which the normality assumption does not need to be satisfied. This test is sometimes referred to as a non-parametric ANOVA. The Conover *post hoc* test has been chosen for having higher power compared to Nemenyi and Dunn tests. Both healthy and diseased states data were used for this analysis.

### Correlation Analysis

We calculated Pearson correlation coefficients of the protein concentrations across different data sets, brain regions and cell types, as well as numbers of common proteins with known concentrations across pairs of data sets, brain regions and cell types. We also calculated numbers of common proteins measured across all combinations of data sets, brain regions, and cell types.

### Factors That Explain Biological Variability of Concentrations

We were concerned that applied transformations could potentially ‘‘overnormalize’’ the data, eliminating natural biological differences. We performed a series of statistical analyses to assess whether subcellular location, functional category or cell type contribute the most to the remaining variability of protein concentrations. We assigned functional categories to proteins using Gene Ontology (MGI-GO slims^1^). We started with a subset of the data from neurons to reduce possible systematic errors due to differences in cell types. Using this data as a case study, we compared concentrations of stress-response related proteins of oxidative stress and DNA repair. These proteins are mostly attributed to different organelles, primarily mitochondria and nuclei. Next, we compared the oxidative stress response and oxidative phosphorylation (mostly mitochondrial proteins). We then examined the variability of all available protein levels in neurons compared to astrocytes. For this analysis, we chose a subset of oxidative stress response proteins. We used only mouse data to perform the comparison of neurons with astrocytes, since the rat and human studies predominantly contain neuron and not astrocyte data. For the subcellular location and functional category analysis, data from mouse, human, rat were used as they were well balanced across compared groups.

We used a series of statistical measures to perform rigorous analysis of protein concentration distributions (both μM and natural log transformed data). We started with the distance between the median to overall visible spread ratio (DBM/OVS) calculation for the first evaluation of whether there is a difference between compared groups (Wild et al., 2011). Then we aimed to examine whether compared groups are likely taken from the same distribution by using the Wilcoxon-Mann-Whitney *U*-test (two-sided) and Kolmogorov-Smirnov test (two-sided). Even though these tests are relatively similar, the Kolmogorov-Smirnov test is sensitive to any differences in distributions (shape, median, spread), while the Wilcoxon-Mann-Whitney test is mostly sensitive to differences in medians. Next we tested for equality of variances using the Brown-Forsythe (modified Levene test to use medians as a centers of compared groups) test (Levene, 1960; Brown and Forsythe, 1974); and the Fligner-Killeen (non-parametric) test (Fligner and Killeen, 1976; Conover et al., 1981), both of which are applicable when data is non-normally distributed. The first tolerates relatively small deviations from normality, and the second is better suited for non-normally distributed data and the data with outliers. Homogeneity of variance is an important assumption of most of the parametric statistical tests. Due to the possible effect of sample sizes, we performed a permutation procedure with 1000 times random sampling (*N* = 100) and repeated comparisons in sampled data. We used sampling from combined data as control. Only healthy-state data was included.

### Comparison With PubMed Mentions

We defined the brain region and cell type specific proteins as those with concentrations in the top 1% of overall protein levels in different brain regions and cell types correspondingly. Next, we defined protein specificity index as a difference in natural logarithms of concentrations for proteins in relation to brain regions and cell types where they are measured compared to their concentrations in other brain regions and cell types correspondingly. We queried PubMed for co-mentions of gene names with cell types in which the concentration of related protein was measured. Then, we compared specificity indices of these proteins with their association (co-mentions) with those brain regions and cell types in the literature obtained by automated PubMed mining using the R programming language (library RISmed^2^). Only healthy state data were used for this analysis. Possible biases in this analysis come from synonyms as well as a tendency to cite influential papers. This analysis should be considered as one of many evaluation steps.

### Functional Network Analysis

As was done for the PubMed mentions analysis, we selected proteins in every cell type (neurons, astrocytes, microglia, oligodendrocytes) and brain region of interest (cerebellum, cortex, hippocampus, striatum, brainstem, thalamus, amygdala) with concentrations above 99% of overall protein levels across cell types and brain regions, correspondingly. Using Cytoscape version 3.7.1 (Shannon, 2003) with STRING plugin (Doncheva et al., 2019; Szklarczyk et al., 2019), we analyzed networks of these proteins in different cell types and brain regions, using the Markov Cluster Algorithm (inflation parameter of 5) and subsequent functional enrichment on clusters using the default parameters to retrieve it with the Cytoscape-STRING plugin. Only healthy state data were used for this analysis.

### Preservation of Differential Protein Expression Patterns

We used concentrations estimated from Hasan et al. (2019) data to examine protein level changes in EAE compared to healthy controls. Only one study was selected for this analysis to diminish possible biases. The data was median-normalized. First, we used principal component analysis (PCA) for dimensionality reduction to visualize the samples. Next, we performed basic differential expression analysis as shown in Supplementary Presentation and the source code. We used the same criteria as in Hasan et al. (2019) to select significantly upregulated (fold change ≥ 1.15; *p* < 0.05) and downregulated (fold change ≤ 0.87; *p* < 0.05) proteins. Centering and scaling with the base R language scale function were performed for the heatmap. We set the “row_km” parameter in ComplexHeatmap (Gu et al., 2016) to two for easier interpretation of clusters. Functional annotation of clusters was obtained using Gene Ontology resource (Ashburner et al., 2000; Mi et al., 2017; The Gene Ontology Consortium, 2019).

#### Comparison of Protein Concentrations Between Species

Using the gene names alignment, we selected common proteins from healthy-state mouse and human samples. For between-species comparison we selected measurements from cortex, striatum, cerebellum, brainstem, hippocampus, thalamus, amygdala based on (Carlyle et al., 2017; Hasan et al., 2019; McKetney et al., 2019; Bai et al., 2020). Only mouse data from Bai et al. (2020) is used in this analysis, because human data from the same study does not reflect a healthy state. The mean concentrations across repetitions of the same species, studies and age categories were calculated, combining data from different brain regions. The aggregated data resulted in concentration entries for 3990 genes for 8 combined samples of different species, studies and age categories. Median normalization was performed to prepare data for PCA and heatmap in Supplementary Figures 7A,B correspondingly. Additional centering and scaling with the base R language scale function were performed for the heatmap. The row_km parameter in ComplexHeatmap (Gu et al., 2016) was set to two for easier interpretation of clusters. We selected only significantly upregulated (log2 fold change ≥ 2; *p* < 0.05) and downregulated (log2 fold change ≤−2; *p* < 0.05) proteins for the heatmap.

#### Comparison of Protein Concentrations Between Cell Types

We applied the same methods to compare protein concentrations between cell types with healthy-state mouse and rat samples based on (Han et al., 2014; Sharma et al., 2015; Chuang et al., 2018; Krogager et al., 2018). The mean concentrations across repetitions of the same studies and age categories were calculated. Median normalization was performed to prepare data for PCA and heatmap plots in Supplementary Figures 8A,B, correspondingly. Additional centering and scaling with the base R scale function were performed for the heatmap in Supplementary Figure 8B, with the same row_km parameter and log2 regulation range as for between species. The list of proteins with these differential concentrations in neurons compared to astrocytes is given on the right in Supplementary Figure 8B, and features some of the known proteins of particular importance in the brain.

#### Case Study of Protein Concentrations in Alzheimer’s Mouse Cortex

Data from two studies (Hamezah et al., 2019; Bai et al., 2020), both of which measured healthy and AD samples, were integrated for this analysis. Median normalization was performed to prepare data for PCA and heatmap plots in Supplementary Figures 9A,B, correspondingly. Centering and scaling were performed for the heatmap Supplementary Figure 9 as in previous analyses. The same row_km parameter was set for easier interpretability of clusters on the heatmap. We used the same criteria as in Hasan et al. (2019) and in our comparison of EAE to healthy state concentrations to select significantly upregulated (fold change ≥ 1.15; *p* < 0.05) and downregulated (fold change ≤ 0.87; *p* < 0.05) proteins in disease compared to healthy state.

### Expansion of the Integrated Data Using RNA-to-Protein Level Predictions

Multi-omics studies, for instance, Sharma et al. (2015) measured both the transcriptomes and proteomes for different brain cell types. We used these data on RNA and protein levels to determine whether we can estimate how protein concentrations in different cell types reflect the differences in mRNA levels. For the initial estimation, we assume that these mechanisms are similar in different brain cell types, ignoring cell-specific regulatory processes. Therefore, we can use reference protein concentrations with reference gene expression from the same study to calculate RNA-to-Protein (RTP) conversion ratios for all the available genes and proteins. Next, we applied the conversion ratios to obtain protein levels from the RNA levels of more specifically separated cell types.

### Metabolite Concentrations at Different Scales

As described in Results, we calculated tissue level signal for the metabolite concentrations based on cellular level concentrations of metabolites and compared it with that measured at the tissue level from other experiments. We used the PubChem compound identifier to resolve synonymic names of molecules. We recalculated reported values per gram of wet or dry tissue to molar concentrations to compare MRS with mass-spectrometry data. We manually validated concentrations of characteristic metabolites against various literature data, by comparing whether there are any values in the Atlas which are no more than twice higher or lower that the other literature data, including glucose (Erecińska and Silver, 1994; Byrne et al., 2014; Barros et al., 2017), ATP (Köhler et al., 2020), lactate (Muraleedharan et al., 2020), pyruvate (Byrne et al., 2014), glutathione (Koga et al., 2011). This approach is very limited and more data on metabolite concentrations is needed for a more complete validation.

### The Molecular Atlas Application in Flux Variability Analysis

We tested the integrated database by applying it to a simulation of metabolism. One common method for simulation of large scale metabolic networks is called flux balance analysis (FBA). We aimed to evaluate whether protein concentrations used as constraints will result in meaningful relative maximum capacities of reactions in the neuron and astrocyte. We provided a detailed description for this part in the Supplementary Presentation (McKenna et al., 2006; Çakir et al., 2007; Lewis et al., 2010; Orth et al., 2010; Sigurdsson et al., 2010; Schellenberger et al., 2011; Ebrahim et al., 2013; Desouki et al., 2015; Gavai et al., 2015; King et al., 2015; O’Brien and Palsson, 2015; Noor et al., 2016; DiNuzzo et al., 2017; Martín-Jiménez et al., 2017; Sánchez et al., 2017; Heckmann et al., 2018; Supandi and van Beek, 2018; Tian and Reed, 2018; Lularevic et al., 2019; Pandey et al., 2019; Anand et al., 2020; Gurobi Optimization, 2021).

### Quantification and Statistical Analysis Summary

Concentration estimations and statistical analysis were performed using Python and R scripts (available from https://github.com/BlueBrain/MADIP; https://github.com/BlueBrain/BrainMolecularAtlas) with commonly used packages (Hunter, 2007; Hagberg et al., 2008; Krijthe, 2015; Silge and Robinson, 2016; Wickham, 2016, 2019; VanderPlas et al., 2018; Terpilowski, 2019; Dowle and Srinivasan, 2020; Harris et al., 2020; Schauberger and Walker, 2020; Virtanen et al., 2020; Reback et al., 2021; Waskom et al., 2021; Wickham et al., 2021). The details can be found in the “Results” and “Materials and Methods” Sections. The chosen statistical tests tolerate deviations from normality. We have chosen to use two-sided tests. Equality of variances was analyzed with Brown-Forsythe (Levene, 1960; Brown and Forsythe, 1974) and Fligner-Killeen tests (Fligner and Killeen, 1976; Conover et al., 1981). Summary on the statistical methods applied in this study is available in the “Materials and Methods” Section and Supplementary Table 2. We did not include data at the subcellular level of detail in the analysis of cellular concentrations, however, this data is available through Supplementary Data Sheets, and the accompanying website https://portal.bluebrain.epfl.ch/resources/models/brain-molecular-atlas.

## Results

### Protein Concentrations Estimation

We need absolute molar concentrations or absolute protein copy numbers for modeling purposes or as one of the possible references. These units are easily interconvertible and more biologically relevant than raw mass-spectrometry intensities.

Therefore, we applied a data integration pipeline (see section “Materials and Methods”) resulting in the Adjusted Molecular Concentration (AMC) database, containing 2,131,244 concentration entries for proteins produced by 14,700 genes (Supplementary Data Sheets 1-4).

**FIGURE 2:**
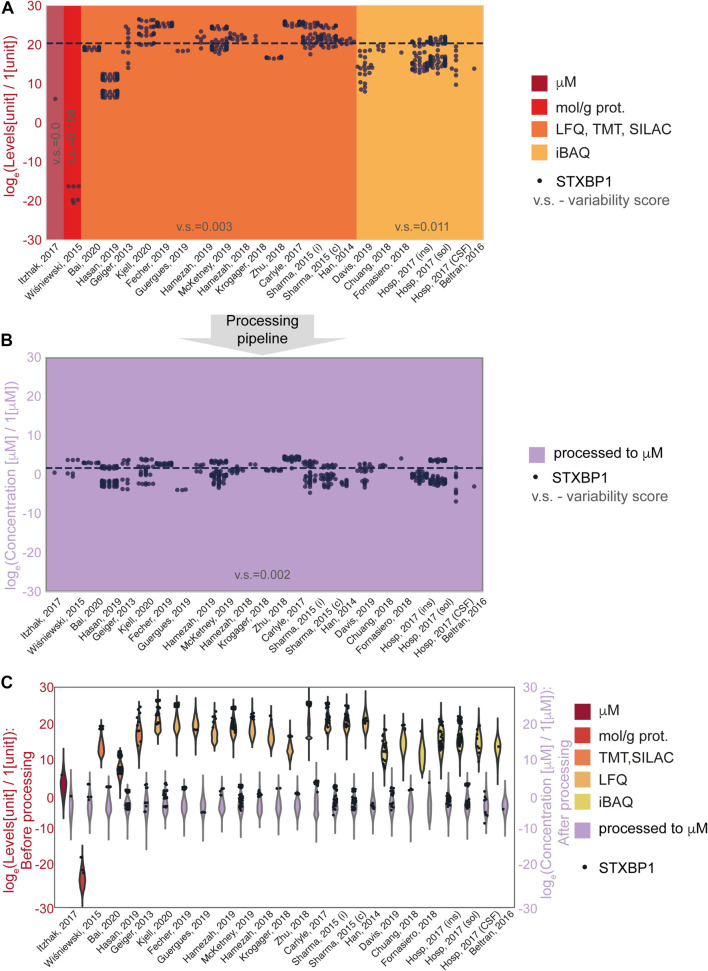
An example of data before and after normalization for the experimental methods used to obtain the data. **(A,B)** STXBP1 protein (Syntaxin-binding protein 1) before **(A)** and after **(B)** methodological normalization. Horizontal line corresponds to the median value and is drawn at the level of 20.3 **(A)** and 1.57 a.u. **(B)**. STXBP1 was chosen for demonstration as the protein with the largest number of available measurements in the collected data among the proteins that are present in the largest number of data sets (590 entries from 24 data sets). Variability score is defined as an absolute value of the coefficient of variation of the data (no log transformation) scaled to the number of measurements of each protein in every data type. The data after methodological normalization is considered as one group for calculation of the variability score in panel **(B)**. **(C)** Protein levels in different data sets before and after normalization. Abbreviations: log_*e*_ = natural logarithm, LFQ = label-free quantification; TMT = tandem mass tag; SILAC = stable isotope labeling by/with amino acids in cell culture; iBAQ = intensity-based absolute quantification. Sample sizes per data set are available from the Supplementary Presentation.

**FIGURE 3:**
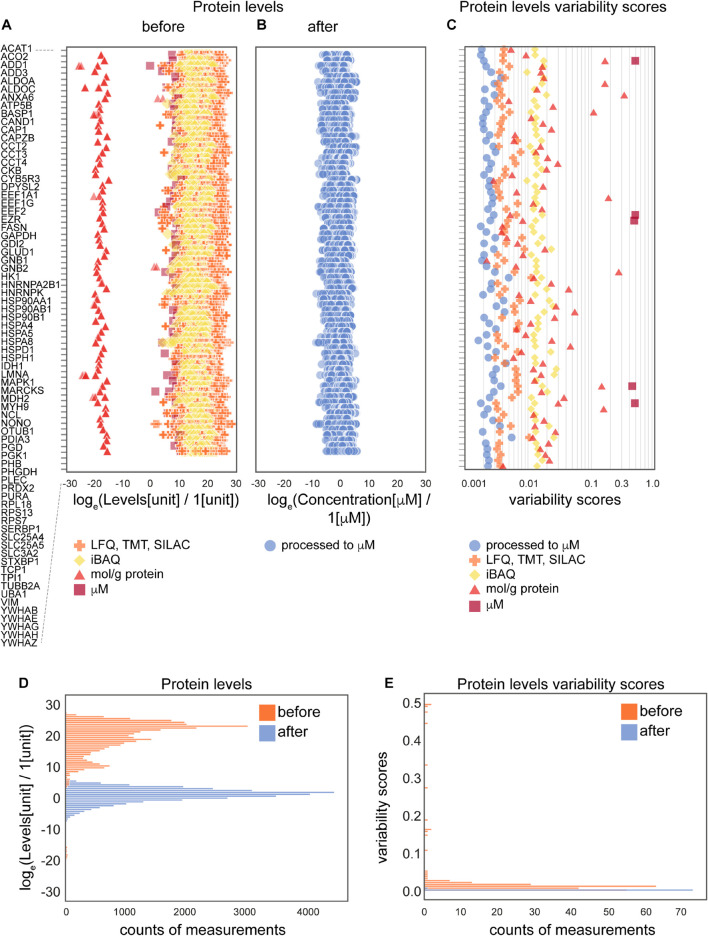
The effects of the data processing pipeline on protein levels. **(A,B)** Protein levels before **(A)** and after **(B)** normalization; and levels variability scores **(C)** of 74 proteins that are present in the largest number of collected data sets (24 of 25 data sets). **(D,E)** Histograms of protein levels **(D)** and variability scores **(E)** for the same set of 74 proteins as in panels **(A–C)**. Abbreviations: log_*e*_ = natural logarithm, LFQ = label-free quantification; TMT = tandem mass tag; SILAC = stable isotope labeling by/with amino acids in cell culture; iBAQ = intensity-based absolute quantification. Sample sizes are available from the Supplementary Presentation.

The effect of the processing pipeline on protein concentrations is shown in Figures 2, 3 with examples of the most represented proteins by the number of measurements in different data sets. We have chosen Syntaxin-binding protein 1 (STXBP1) for demonstration of the effect of concentration estimation and normalization, as the protein with the largest number of available measurements in the collected data among the proteins that are present in the largest number of data sets (590 entries from 24 data sets). Figure 2A shows levels of STXBP1 protein in original data. Normalized concentrations of this protein are reflected in Figure 2B. As expected, between-data set variation is reduced as a result of unit unification and normalization. Due to the importance of relative concentrations of the protein in comparison with other measured proteins, we show levels of STXBP1 along with distribution of levels of other proteins before and after processing (Figure 2C). We can see that STXBP1 tends to be among highly expressed proteins, which is in line with the fact that it was detected in the largest number of collected samples. In summary, our processing pipeline brings together non-homogeneous quantitative data on protein levels reported in different units, and produces widely used molar concentrations.

### Protein Variability Across Data Types

To further assess the effect of the applied data transformation, we compared the original (Figures 3A,D) and processed concentration levels (Figures 3B,D) of 74 most represented proteins by the number of measurements in different data sets. Since individual proteins in the original data are reported in different units in non-equal numbers of biological settings (brain regions, ages, cell types and parameters) of non-equal sample sizes of different types of experiments, we defined variability score as an absolute value of the coefficient of variation of the data (no log transformation) scaled to the number of measurements of each protein in every data type. A zero-variability score means that the coefficient of variation of the protein level data for a given type of protein is zero. These entries are omitted from visualization in Figures 3C,E to improve the figure’s readability. From the comparison of unitless variability scores calculated for original and processed data, we observed a decrease of variability upon processing (Figures 3C,E). With this analysis, we demonstrated that molar concentration calculations with subsequent normalization makes the data from different types of sources more comparable and prepares them for use in further studies. The difference in variability addressed in this analysis mostly comes from the difference of original units, most of which are not comparable without translation to a common unit system (such as molar concentrations or protein copy numbers).

### Validation

We further assessed and validated the integrated Molecular Atlas data in a series of analyses as described below.

#### Comparison of Absolute Values of Protein Concentrations to Published Data

The aim of the next integrated data assessment step was to evaluate the absolute scale of estimated molar concentrations. Both healthy and diseased states data were used for this analysis. Even though absolute levels, such as concentrations or copy numbers are essential, the gold standard is scarcely available for the large number of proteins in mammals. As an initial quality check, we evaluated total protein count per cell from the calculated copy numbers based on the normalized concentrations data and initially estimated volume (see formulas in section “Materials and Methods”), and compared those numbers to the literature evaluations (Milo, 2013), as shown in Supplementary Figure 2A. We compared the concentration of signaling proteins from our data with the characteristic range of signaling protein concentrations 0.01-1.0 μM from literature (Milo et al., 2010). Using the Mouse Genome Database (Bult et al., 2019) we obtained a list of 6,087 signal transduction genes (GO:0007165). We used gene symbols as a key to a subset of the Brain Molecular Atlas for signal transduction genes, and we found 3,349 relevant gene names in the collected data. The median concentration of signal proteins in the Brain Molecular Atlas is 0.087 μM after the normalization procedure, which is in the range of literature values (Milo et al., 2010). The signaling protein concentrations distribution is shown in Supplementary Figure 2B. We conclude that estimated molar concentrations are in the range of biologically plausible values at the absolute scale.

#### Consistency Check of Predicted Concentrations From Data Set Comparisons

As only housekeeping genes were used in the normalization procedure, we next decided to statistically evaluate the equality of medians from full data sets to see how comparable they are. Holm adjusted *p*-Values from the Conover *post hoc* test applied after Kruskal-Wallis Test (*H* = 1034.55, *p*-Value = 4.07e-203) for comparison of normalized concentrations (on natural log scale) from different studies are shown in Supplementary Figure 2D. Due to the particular importance of peptides for neuroscience research (Hökfelt et al., 2000; Borbély et al., 2013) we examined the consistency of their concentration distributions across different data sets by the same approach (Supplementary Figures 2C,E). According to the test results, medians of estimated concentrations are largely in agreement across studies, however, one can see that distributions of concentrations in some pairs of studies still have significant differences. This can be explained by the different sets of brain regions from where the data were obtained, cultured or isolated cells, different developmental stages, sexes, presence of both control and disorder state data in some data sets, and other biological parameters for which no control was introduced in this analysis, as well as potential limitations of the analysis itself. However, this result is confirmatory in a sense that we do not expect to precisely match all distributions, because this would discard the natural variability of protein levels in different biological settings.

#### Correlation Analysis

**FIGURE 4:**
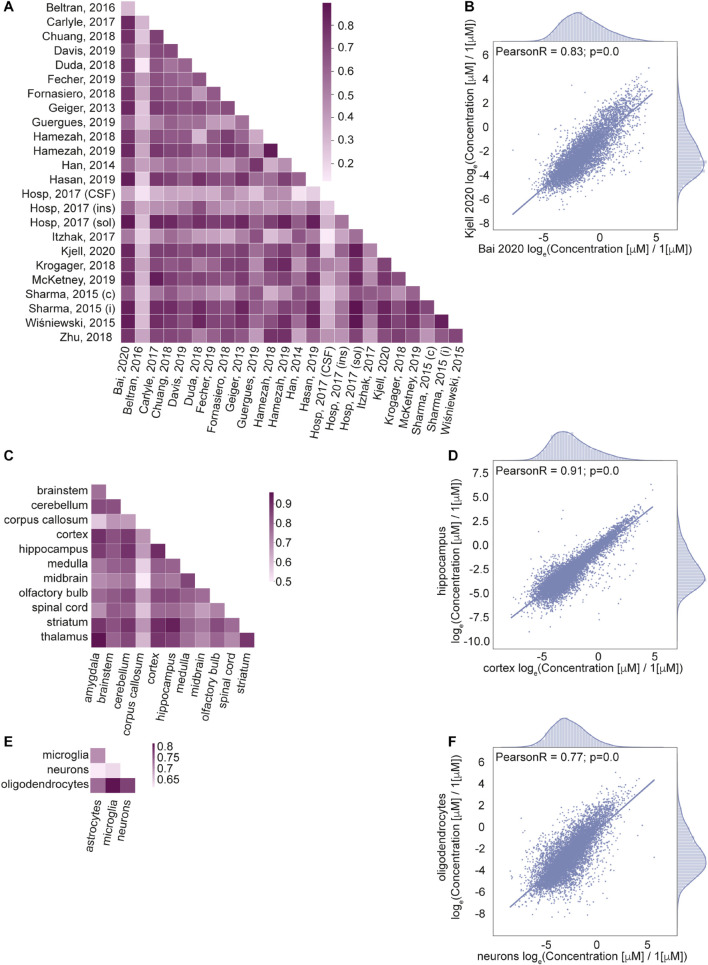
Correlation of protein concentrations confirms good agreement of the integrated data. **(A,C,E)** Pearson coefficient of correlation for protein concentrations from different studies **(A)**, brain regions **(C)**, cell types **(E)** after normalization. **(B,D,F)** Representative examples for the comparison of protein concentrations data from two studies **(B)**, two brain regions **(D)**, and two cell types **(F)**. Natural logarithm for μM concentrations is used in all panels.

Next, we wanted to know how well correlated the estimated protein concentrations are across different data sets (Figures 4A,B), brain regions (Figures 4C,D) and cell types (Figures 4E,F). We calculated Pearson correlation coefficients of the protein concentrations as well as the number of common proteins with known concentrations across pairs of data sets (Supplementary Figure 3A), brain regions (Supplementary Figure 3C), and cell types (Supplementary Figure 3E); see Section “Materials and Methods” for the details.

The number of common proteins (i.e. sample size) in compared data sets is important for the interpretation of the correlation analysis results. It would be ideal to use the same set of proteins in every pair of samples in correlation analysis. The analysis indicates that the number of common proteins decreases with the number of different samples taken together (Supplementary Figures 3B,D,F), as the coverage of measured concentrations in every sample does not correspond to a full proteome. So, we cannot choose a set of proteins measured among all data sets which would permit correlation analysis on the same list of proteins for comparisons of all pairs of the data sets, brain regions, cell types. For this reason, the correlation coefficient is calculated independently for every pair of samples (data sets, brain regions, cell types) using the list of proteins that are measured in common in the two samples of every comparison. Information on the sample sizes of every pair is given in Supplementary Figures 3A,C,E.

The correlation analysis shows high correlation for most of the pairs of samples across studies, brain regions and cell types. Lower correlations of the data based on Jean Beltran et al. (2016) compared to other sources are due to the cell type (primary human fibroblast) used in the study. However, this data was included in our integrated database because of the importance of organellar scale concentrations, which are rarely found in genome scale studies to date. To summarize, the high level of correlation among the data sets that are expected to produce similar cellular protein portraits and lower level of correlation between data sets that come from very different biological settings further validate applied data transformation.

#### Factors That Explain Biological Variability of Concentrations

Our pipeline aimed to reduce experimental biases in the data, but we were concerned that the approach could lead to the elimination of the natural biological variation of concentrations. Accordingly, we sought to address the factors which explain the remaining variability of protein concentrations after we applied the processing pipeline. For instance, concentrations for some proteins fall into a wide range of values in a healthy state, potentially elucidating adaptation mechanisms of cellular homeostasis and stress response pathways. We applied several statistical tests to analyze the contributions of those factors in a case study of different subcellular locations, functional categories and cell types (see section “Materials and Methods”).

**FIGURE 5:**
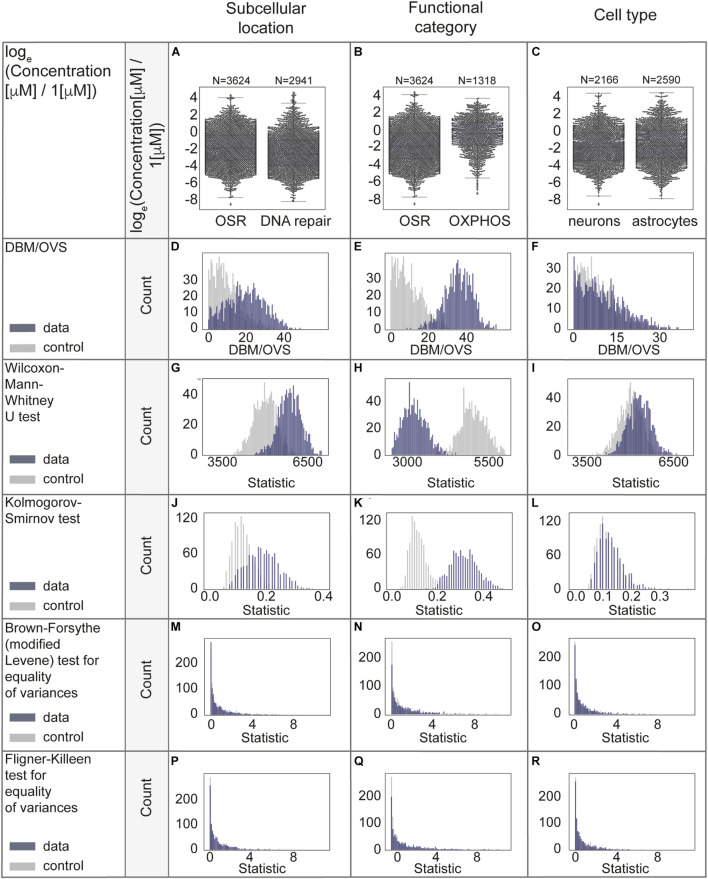
Statistical evaluation of factors with a potential to explain biological variability of protein concentrations. **(A–C)** Comparison of protein concentrations in different groups of proteins. Boxplots characteristics: **(A)** OSR (oxidative stress response): center line, median: −1.86; upper and lower quartiles: Q1: −3.31, Q3: −0.32; whiskers, 1.5x interquartile range: −7.77, 4.14; outliers: −8.57, 4.39, 4.18. DNA repair: center line, median: −2.56; upper and lower quartiles: Q1: −3.92, Q3: −0.95; whiskers, 1.5x interquartile range: −8.24, 3.48; outliers: 3.74, 3.36, 3.76, 3.58, 3.62, 4.40, 4.57, 4.30, 3.71, 4.36, 3.94, 3.87, 4.45, 4.70, 4.15. **(B)** OSR (oxidative stress response): center line, median: −1.86; upper and lower quartiles: Q1: −3.31, Q3: −0.32; whiskers, 1.5x interquartile range: −7.77, 4.14; outliers: −8.57, 4.39, 4.18. OXPHOS (oxidative phosphorylation): center line, median: −0.35; upper and lower quartiles: Q1: −1.74, Q3: 0.90; whiskers, 1.5x interquartile range: −5.56, 3.72; outliers: −7.34, −5.87, −6.35, −6.15, −6.59, −6.87. **(C)** Neurons: center line, median: center line, median: −1.96; upper and lower quartiles: Q1: −3.44, Q3: −0.26; whiskers, 1.5x interquartile range: −7.63, 4.39; outliers: −8.57. Astrocytes: center line, median: −1.74; upper and lower quartiles: Q1: −3.17, Q3: 0.00; whiskers, 1.5x interquartile range: −7.93, 4.43; no outliers. **(D–R)** Statistical analyses in permutations with multiple (1000) resampling with the sample sizes of 100. Types of analyses are named in the left panel of each row.

The case study results for comparison of distributions of protein concentrations of different subcellular locations, functional categories and cell types are represented in Figures 5A-R and Supplementary Table 2, additional results (without log-transformation) are in Supplementary Figures 4A-O. From the analyses above we conclude that the functional category is the factor which best explains the remaining protein concentration variability.

#### Literature Associations and Cell Type Specificity of Proteins

The next step in the evaluation of generated data aimed to evaluate relative levels of protein concentrations across brain regions and cell types. We queried PubMed for co-mentions of gene names with cell types in which the concentration of related protein was measured. We defined a protein specificity index (in section “Materials and Methods”) as a measure of the protein concentration in a particular location (cell type, brain region) as related to other locations of the same level of detail (cell types or brain regions, correspondingly). We observed a weak association between a protein specificity index in a particular cell type with the number of co-mentions of the corresponding gene and that cell type (Supplementary Figures 5A–D).

There was no relation of brain region protein specificity with PubMed co-mentions of the proteins with corresponding brain regions. Different noise factors, such as synonyms to protein names, contribute to the imprecision of this association analysis. It should be considered only as one of the evaluation steps, which requires a more detailed approach when studying potential protein markers. We conclude from this analysis that more knowledge is available in the literature on the cell-type specific protein/gene expression for the proteins having concentration highly specific to particular cell types in our data, than on the analogous comparison of the brain region specificity.

#### Functional Analysis of Protein Networks

**FIGURE 6:**
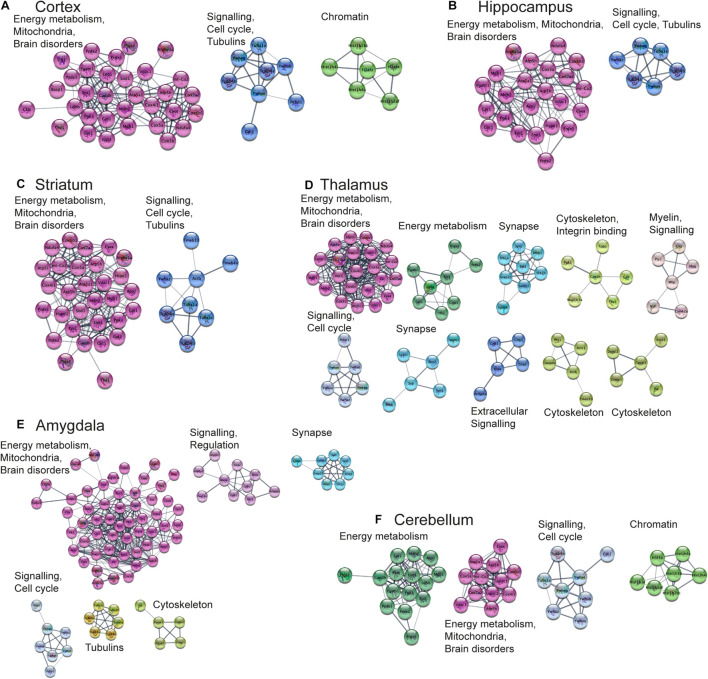
Functional analysis of the most expressed proteins highlights shared functions in different brain regions. Networks of the most expressed proteins in different brain regions. **(A–F)** Nodes represent proteins. Edges correspond to all known relations between proteins based on STRING-Cytoscape (Shannon, 2003; Doncheva et al., 2019; Szklarczyk et al., 2019). Only clusters with more than 4 nodes are shown. The version of this figure with labels shown using bigger font size is available from the Supplementary Presentation for better readability of the labels.

**FIGURE 7:**
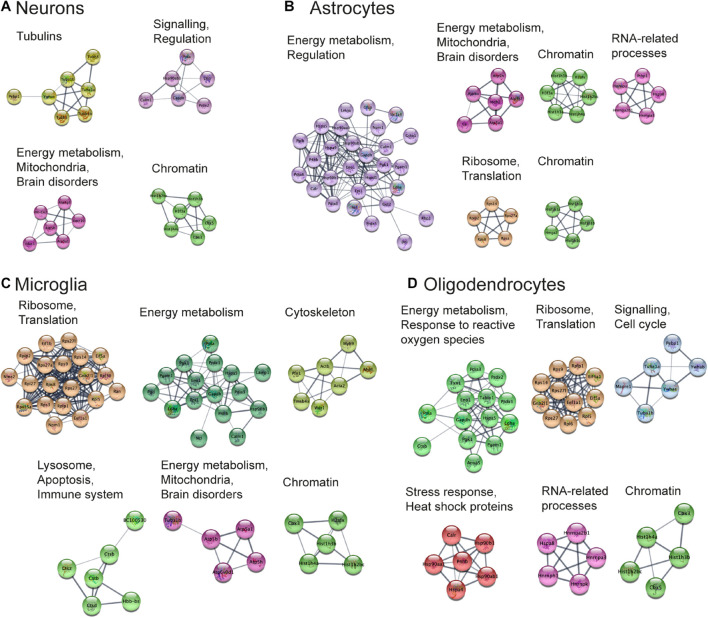
Functional analysis of the most expressed proteins highlights shared functions in different cell types. Networks of the most expressed proteins in different cell types. **(A–D)** Nodes represent proteins. Edges correspond to all known relations between proteins based on STRING-Cytoscape (Shannon, 2003; Doncheva et al., 2019; Szklarczyk et al., 2019). Only clusters with more than four nodes are shown. The version of this figure with labels shown using bigger font size is available from the Supplementary Presentation for better readability of the labels.

Next, we aimed to perform functional analysis of overrepresented proteins across brain regions and cell types and compare the results to the literature. This was performed using the Cytoscape software version 3.7.1 (Shannon, 2003) with STRING plugin (Doncheva et al., 2019; Szklarczyk et al., 2019) as described in “Materials and Methods” Section. Networks of these proteins for selected brain regions and cell types are shown in Figures 6, 7, clusters of less than four nodes are omitted in the visualization. More detailed results on functional annotation are in Supplementary Data Sheet 6. Different brain regions and cell types share many annotations, such as energy metabolism and mitochondria, brain disorders, signaling, chromatin, and others, that are enriched in the overrepresented proteins. Indeed, energy metabolism possesses significant cell-type specific properties (Magistretti and Allaman, 2015) and shows brain-region dependent differences (Kleinridders et al., 2018). Individual variations are also represented, for instance, the immune properties of microglia, which is in agreement with literature (Lenz and Nelson, 2018). We found that stress response and heat shock proteins are enriched in oligodendrocytes, and the literature evidence confirms this observation (Goldbaum and Richter-Landsberg, 2001).

#### Preservation of Differential Protein Expression Patterns

**FIGURE 8:**
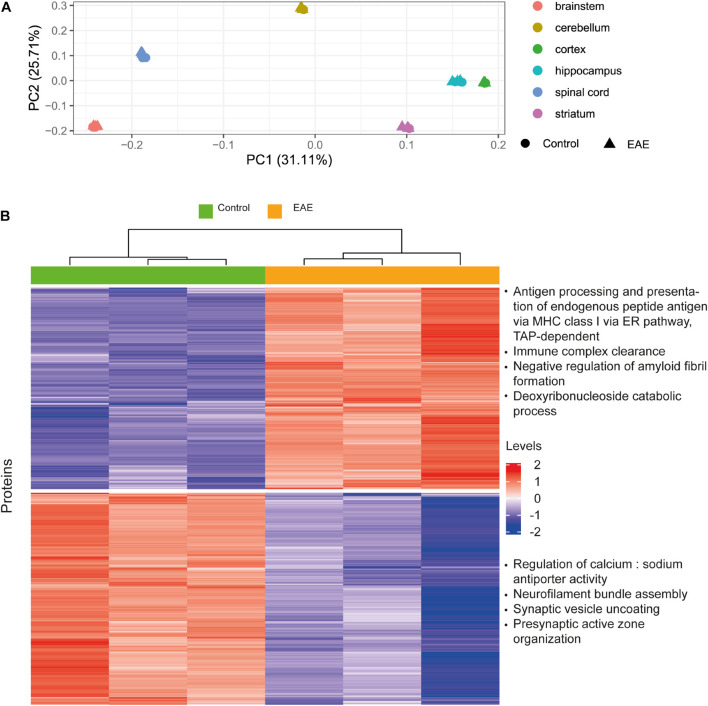
Case study for differential protein concentrations analysis across brain regions and states. **(A)** Principal component analysis performed on the Molecular Atlas protein concentrations estimated from Hasan et al. (2019) data. **(B)** Hierarchical clustering of proteins with differential concentrations in EAE spinal cord samples compared to healthy spinal cord (based on 3128 proteins). Top four enriched GO biological process terms are shown on the right of the corresponding cluster. Levels refer to row-scaled centered median-normalized Molecular Atlas concentrations.

The aim of this analysis was to assess the reliability of estimated protein concentrations in preserving differential expression patterns. We performed differential expression analysis on the basis of molar concentrations for a subset of proteins from Hasan et al. (2019) which was already included as a data source in our pipeline, and compared the results to the original report. Our idea was to analyze whether the processing pipeline disturbs data in a way that differential expression patterns observed from the mass-spectrometry protein abundances will not be observed when using estimated concentrations. We observed better separation for brain regions rather than healthy and diseased states in principal component analysis (Figure 8A). On this basis, we analyzed differentially expressed genes (Robinson et al., 2010; Ritchie et al., 2015) in distinct brain regions, not combining the data from different brain regions (Figure 8B and Supplementary Figures 6A-F). The top four enriched Gene Ontology biological processes terms are shown on the right of the corresponding cluster in Figure 8B. The resulting enriched biological processes are in good agreement with those reported in Hasan et al. (2019), in particular, immune mechanisms are upregulated and synaptic processes are downregulated in EAE spinal cord samples, as found by both our analysis using concentrations and the Hasan dataset. We conclude that estimated concentrations preserve differential protein expression patterns in the comparison of EAE samples to healthy controls; but more variance in the protein concentrations is explained by the brain regions of origin, rather than diseased-state versus control.

#### Comparison of Protein Concentrations Between Species

To further assess whether estimated concentrations preserve differential protein expression, we focused on species-specific differences. We applied the same approach as in the previous analysis (Preservation of differential protein expression patterns) to find proteins with differential concentrations between mouse and human. There was a clear separation between mouse and human brain samples after the PCA was applied for the dimensionality reduction (Supplementary Figure 7A). We also found a set of proteins with differential concentration levels in the mouse and human brain, so we conclude that the integration pipeline preserved between-species biological variation in the protein concentrations (Supplementary Figure 7B). More detailed analyses, which are out of the scope of this study, need to be carried out to compare differential protein concentrations to the differential expression analysis performed using proteomics data without transformation to concentrations.

#### Comparison of Protein Concentrations Between Cell Types

Similarly to cross-species comparison, we assessed whether the differences in protein concentrations are preserved in different cell types. Specifically, we compared concentrations in astrocytes and neurons on the basis of multiple studies (Supplementary Figure 8). The methods (described in the corresponding section) are analogous to the previous section on species. We can see that it is possible to separate neuron from astrocyte samples using integrated data on protein concentrations, where one of the data sources provides the information on both neurons and astrocytes, and other resources report the data for only one of those.

#### Case Study of Protein Concentrations in Alzheimer’s Mouse Cortex

As protein concentrations might represent more biologically relevant units compared to mass-spectrometry intensities, we aimed to use the generated data to compare healthy-state with AD using the same methods as in the previous section (see “Materials and Methods” for the details). From this analysis, we found a list of proteins that are present at differential concentrations in healthy and AD states (Supplementary Figure 9). Among the proteins of that list are Amyloid Beta Precursor Protein (APP), Annexin A3 (ANXA3), Lysosomal Associated Membrane Protein 2 (LAMP2), Late Endosomal/Lysosomal Adaptor MAPK And MTOR Activator 2 (LAMTOR2) that are known for the involvement in the AD pathology according to the literature data (Sjödin et al., 2016; Castillo et al., 2017; Navarro et al., 2020).

Differential protein concentration analyses in Figure 8 and Supplementary Figures 6-9 further confirm that concentrations in the Molecular Atlas preserve within-individual biological variation of concentrations, which permits the observation that some molecules have different concentrations across brain regions and cell types, as well as in different states, and others are more uniform in varying locations and conditions. Lists of differentially expressed proteins are given in Supplementary Data Sheet 7. However, more attention is needed for the analysis of potential confounding variables when the data are applied to the search of potential disease, species, cell types, and brain regions markers.

#### Expansion of the Integrated Data Using RNA-to-Protein Level Predictions

The aim of the next analysis was to explore potential use of RNA sequencing data for prediction of protein concentrations. Even with a high overall coverage of quantitative data for protein levels in the brain, there is a lack of cell-type-specific resolution (e.g., for different morpho-electrical types of neurons) for protein concentrations, and not all brain regions are covered by protein level data, which are needed for simulations. However, RNA sequencing, and especially single-cell RNA sequencing, features high resolution and coverage of various morpho-electrical types of neurons. For this reason, we can calculate approximations for the differences of protein concentrations in various cell types and brain regions using gene expression data.

Regulatory mechanisms of protein turnover can distort the correlation between RNA and protein levels. But even though the dependence of protein levels on RNA levels is an unresolved question, there is significant evidence that levels of at least some groups of proteins can be predicted from their RNA levels (Vogel et al., 2010; Schwanhäusser et al., 2011; Edfors et al., 2016; Silva and Vogel, 2016; Li et al., 2017; Mandad et al., 2018; Eraslan et al., 2019).

Multi-omics studies allow us to investigate relations between levels of transcripts, proteins, and metabolites. Using (Sharma et al., 2015) data, we calculated RNA-to-Protein (RTP) conversion ratios for all the available genes and proteins data. Next, we applied the conversion ratios to obtain protein levels from the RNA levels. Indeed, protein concentrations independently calculated from transcriptomics RPKM data and proteomics LFQ data among astrocytes and among neurons show a high Pearson correlation (Supplementary Figure 10A). However, correlation between different cell types is lower, and that observation is different from what is expected based on reports on RTP being independent of the tissue (Edfors et al., 2016).

Surprisingly, observed correlations are higher than those reported for comparisons of “raw” mass-spectrometry LFQ levels of proteins and RPKM from transcriptomics (Supplementary Figure 10B). Therefore, we conclude that the transcriptomics data could potentially be used to augment the Brain Molecular Atlas for specific cases such as brain disorders, even though transcriptomics data should be taken cautiously due to the reasons described above.

### Data Integration for Metabolite Concentrations

We aimed to supplement our protein concentrations atlas with data on metabolite concentrations to enable more complete quantitative portraits of the brain cells and regions. The metabolite concentration part of the Molecular Atlas is less comprehensive than the protein part since there are only a few recent studies that quantitatively measured large numbers of metabolites in the brain cells of rats, mice, or humans (Sugimoto et al., 2012; Chen W.W. et al., 2016; Zheng et al., 2016). Two dominant experimental methods are based on either mass-spectrometry (MS) or magnetic resonance spectroscopy (MRS) measurements. Mass-spectrometry studies provide data at different scales of resolution varying from tissue (Kim et al., 2014) to organelle level (Chen W.W. et al., 2016). Spectroscopy experiments usually just report tissue signals. The main experimental data was augmented by commonly known concentrations from review papers and estimations.

**FIGURE 9:**
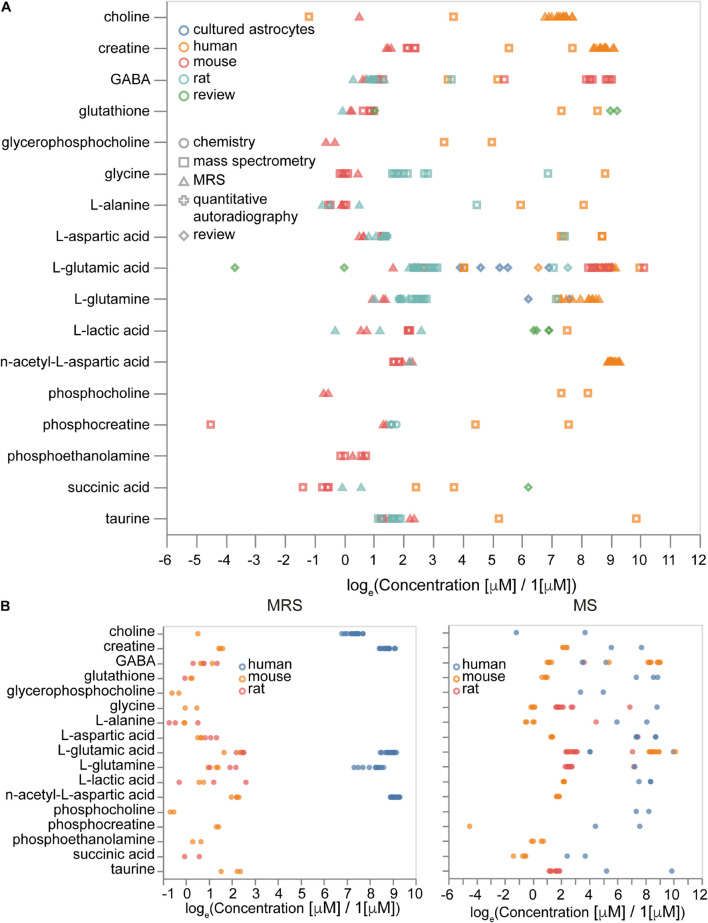
Sources of variability in metabolite concentrations. **(A)** Effects of measurement methods and species on the concentrations of metabolites. **(B)** Concentrations of molecules measured by magnetic resonance spectroscopy (MRS, left) and by mass-spectrometry (MS, right) with the same set of molecules measured by both MRS and MS. Sample sizes are available from the Supplementary Presentation.

Overall, we collected 3,279 concentration entries for 441 unique metabolites. Some of them are annotated at the tissue scale, others are described in particular cell types and subcellular compartments. We analyzed how differences in experimental procedures and organisms affect metabolic concentrations. We observed that the type of experiment (MRS or MS) contributed mostly to variance, rather than the absolute values of concentrations (Figure 9A), while metabolites data from different organisms often show differences in concentrations themselves (Figure 9B). Therefore, it is important to be organism-specific and try to correct organismal differences in metabolic levels when we use data from sources other than the target organism. Moreover, some differences in concentrations often can be explained by variations in experimental protocols.

We calculated brain concentrations from compositions of neuronal, astrocytic, blood and extracellular space concentrations and compared them with brain tissue concentrations for both organism-corrected and initial values of concentrations to validate organismal bias. For this analysis we used the following volume fractions approximated from various literature sources: extracellular space is 0.19 (Hrabetova et al., 2018), vasculature is 0.03 (Santuy et al., 2018), neuronal somas are 0.10 (Santuy et al., 2018), axons and dendrites are 0.60 and glia is 0.08. The volume fractions of axons and dendrites and glia are estimates to separate neuronal and glial components of neuropil. We approximated missing concentrations in astrocytes with neuronal concentrations (otherwise there were only five common molecules with no missing data in all types of volume fractions). Concentrations (without normalization) give a Spearman coefficient of correlation of 0.59 (Pearson coefficient of correlation is 0.13). However, the number of molecules with known concentrations in all volume fractions was too small (23 molecules) for statistical significance. To summarize, integrated metabolite concentrations from different types of experiments can be combined when there is no data for all molecules of interest from the same experiment, but more attention and potentially additional normalization is required if there is a need to work with evolutionary distant species, such as human versus mouse and rat.

### The Molecular Atlas Application in Constraint-Based Modeling

Our next aim was to demonstrate one of many possible applications of the Brain Molecular Atlas by using it for constraint-based modeling of metabolism. Detailed results are available from the Supplementary Presentation. By performing this exercise, we have shown that estimated protein concentrations can be used as flux constraints in metabolism modeling, and the difference in flux capacities reflects expected differences between neuron and astrocyte metabolism. Including experimental data on measured fluxes will further narrow down mathematical solutions to those that are more biologically plausible, potentially improving existing models, but it is out of the scope of the current study. Similar approaches might be taken to compare reaction capacities in healthy and diseased states, bringing more aspects for interpretation of the molecular profiles.

### Brain Molecular Atlas Web Application

While all the generated data, as detailed by different ages, species, locations and conditions with their provenance and meta-information, are available from Supplementary Data Sheets and should be used for any formal evaluation, we also provide an online resource for quick exploration and visualization of the median normalized protein concentrations in different brain regions, as well as neurons and astrocytes with their subcellular locations. The integrated protein data in our Brain Molecular Atlas is publicly accessible through the Blue Brain Cell Atlas (Erö et al., 2018) for protein concentrations in different brain regions, and Blue Brain Protein Atlas for different organelles and whole cell protein concentrations data in neurons and astrocytes. Both can be accessed from the webpage: https://portal.bluebrain.epfl.ch/resources/models/brain-molecular-atlas.

## Discussion

The study of cellular biomolecular networks is required for a more advanced understanding of brain function and disease, for molecular systems simulations, meta-analysis of molecular networks, and as guidance for future experiments. Knowledge of biologically plausible ranges of concentrations is essential for building relevant models. Concentrations of molecules not only define the presence of particular reactions in cells, but also contribute to the rate of reactions and transport between compartments.

Computational representations of the quantitative aspects of cellular biochemical networks have been hampered by discrepancies in experimental methods, data analysis and modeling of molecular species expression and concentration. To address these issues, we performed a meta-analysis that implements data integration and normalization procedures for reported protein and metabolite concentrations from a wide range of sources for mouse, rat and human brain studies. This permitted calculation of Adjusted Molecular Concentrations (AMCs) that formed the basis of the Brain Molecular Atlas. Integrated resources allow multi-aspect analysis of the data and inform experimental design (Fernandes and Husi, 2017; Ho et al., 2018).

We applied a variety of evaluation techniques (see Validation in Results) to assess different aspects of the integrated data, such as biological plausibility of the range of estimated molar concentrations at the absolute scale, correlation analysis, preservation of natural biological variability and factors which explain it, literature associations, functional enrichment of protein networks and discriminative power in differential expression analysis. We showed that the biases introduced by differences in experimental protocols and data processing can be compensated by our pipeline, while preserving biological variability. The cross-study AMCs further revealed the reproducibility of many proteins, suggesting their tight regulation. The remaining biological variability and the dynamic nature of the levels of molecules in organelles, cell types and brain regions determine the kinetics of all biochemical processes (Lundberg and Borner, 2019).

Use of multiple data sets helps to overcome limitations of individual studies and leads to a more complete understanding of molecular systems. For example, new brain cell-type signatures can be found through data integration (McKenzie et al., 2018). Also, there are fewer possible sources of bias when experiments of different types are performed together in multi-omics studies (Angelidis et al., 2019). Even though the number of factors contributing to statistical error increases with the number of divergent data sources, the substantial amount of data required for systems biology modeling are often only available from multiple studies. Integrated data help build these large-scale models that are cell-type and brain region specific. Normalization for methods used to experimentally generate data is essential before combining the information and considering confounding variables when working with data from different studies. The AMC calculation is one such solution and the resulting Brain Molecular Atlas is designed to be expandable and adaptable to new experimental data.

### Insights

We highlighted the importance of critical data assessment, nomenclature alignment, data processing and normalization to the reproducibility of molecular concentrations across studies. Molecular concentrations measured by different protocols can differ by orders of magnitude. For metabolites, this can be related not only to biological variability, but also to the low chemical stability of metabolites and the delay between sampling and inactivation of metabolism (Tillack et al., 2012). Differences in experimental protocols in proteomics studies can lead to systematic errors and discrepancies when comparing data from diverse studies. Moreover, when measuring tissue level signals, extracellular space and different cell types contribute to the cumulative signal, even though the distribution of molecules in different components of the tissue can be non-uniform.

By processing data with respect to the experimental source and normalizing the resulting concentrations to the combination of the most relevant available data as an anchor for normalization, we can significantly decrease experimental methods’ biases. We utilized concentration data integrated from samples of healthy young- to middle-aged mice and mouse cell lines for the list of mouse housekeeping genes (Hounkpe et al., 2021) as reference for median normalization, under the assumption that the concentrations of these proteins are the most conservative. The reliability of such adjusted molecular concentrations (AMCs) demonstrates that the available literature data is sufficient to obtain approximate quantitative molecular characterizations for brain regions and cell types.

Furthermore, studying the biological ranges of healthy-state concentrations can help us understand homeostatic maintenance and regulatory processes of cell metabolism, as well as transitions to disease-states. Notably, different cell types do not exhibit significant differences in bulk protein concentration distributions. But interestingly, categories of proteins related to specific functions show concentration variability, including those that take place in the same organelles. This can be related to particular adaptation roles of some biological processes and an evolved ability to adapt to changing conditions.

### Limitations

The first limitation of this study is the use of the data from both *in vitro* and *in vivo* conditions, which can lead to potential biases in the integrated data. Another tradeoff is supplementation of sparse mouse data with rat and human data. Our goal is to make the best possible estimate for concentrations in different brain regions, cell types, subcellular locations and ages, primarily focusing on the mouse brain. From the validations above, we decided that the benefits of combining sources to obtain more complete coverage outweigh the disadvantages.

Meta-analysis research encounters challenges of nomenclature discrepancy, even though there are several commonly used gene and protein identifiers including UniProt (The UniProt Consortium, 2017) accession, gene symbol and name (Wain et al., 2002; Sundberg and Schofield, 2010; Braschi et al., 2019), Entrez gene (Maglott et al., 2011) and many others. While mapping between different types of identifiers became a routine task, the evolving nature of the nomenclature within even one namespace poses a problem of correspondence between obsolete and current identifiers. One possible solution to the nomenclature problem is to analyze all needed data starting with the raw data. However, the resources and expertise needed to implement this strategy every time some reference data is required for a new experiment or modeling study are often out of scope. Multi-species nomenclature mapping adds another degree of complexity. More universal nomenclature alignment challenges will benefit from the ontological approach and linked data, which will keep the history of different gene and protein identifiers and their mapping to corresponding entries in other namespaces, as well as orthologous relations.

Concentration estimation using the total protein mass approach (Wiśniewski et al., 2014) is based on the number of common assumptions as given in the “Materials and Methods” Section (total cellular protein concentration, protein amount per cell). This could be improved by using cell-type and tissue specific numbers for those parameters. Furthermore, use of the total protein mass approach (Wiśniewski et al., 2014) requires significant coverage (corresponding to thousands of genes) of the proteome in the data sets for the assumptions of the method to be satisfied, which excludes the studies with a small number of proteins reported. This could potentially affect the precision of our concentration estimation for the CSF data set from the study of Hosp et al. (2017).

Furthermore, our normalization is based on the assumption of conservative levels of expression of housekeeping proteins across the integrated data, which potentially may be violated in some cases (for instance, disease states). This can also have flaws when some housekeeping proteins are not measured in some of the data sets, which further impose a high coverage of the proteome requirement for the data sets.

Metabolite concentrations data are still very sparse. Large scale modeling studies would benefit from the data generated with sensitivity at the level of targeted metabolomics, but at the coverage level of untargeted metabolomics. Currently, many studies report levels of molecules known to play an important role in some biological functions, but many other metabolites lack precision on the quantitative characterization of their concentrations.

More independent data sets on the molecular concentrations would be needed for more definitive validation. All estimations as well as all experimental measurements should be critically assessed prior to further use.

### Alternatives

The first alternative to the entire adjusted molecular concentrations pipeline described here would be to experimentally measure all the data in a large series of experiments. This approach is very demanding and any other research method is not guaranteed to be free from the potential errors and standardization challenges that motivated our approach.

The next alternative to the protein concentrations evaluations part would be to start from the raw mass-spectrometry data and perform identification, annotation and quantification of proteins using the same methods for all the data sets. This approach would be beneficial for the nomenclature discrepancies question and for the potential biases introduced by the variations in the mass-spectrometry data analysis.

Targeted proteomics data could be considered, but it would require even more effort in bias correction as a larger number of datasets will be needed to achieve similar data coverage due to the smaller data set sizes usually produced by targeted approaches.

Next, as described above, the assumptions on the total cellular protein concentration and protein amount per cell estimation of concentration could be eliminated in favor of more cell-type and brain-region specific data. This would increase the precision of the estimates.

Another alternative, which has common reasoning with the methods of the current study, would be to evaluate relative protein levels and then use some scaling factor to translate the relative levels into concentrations.

Various alternatives for the normalization step of the pipeline were considered, including but not limited to median normalization using all data entries from particular selections of proteins whose concentrations are expected to be conserved among the tested samples, quantile normalization, more complex statistical models for normalization, and blind normalization (Ohse et al., 2019). But these alternatives were not selected as a final choice since the assumptions of these methods are not met in the protein levels data integration. There are many other methods for the normalization and batch correction in proteomics studies, but they usually require more information about every experiment than is available for the significant selection of divergent studies.

### Applications

By integrating quantitative data, the Brain Molecular Atlas provides a valuable resource for simulations of brain metabolism, analysis of biochemical networks of brain cells, control data for the study of brain disorders and guidance for future experiments. For any particular protein, one can quickly assess its concentration profile in multiple locations in the brain under different conditions using the Brain Molecular Atlas Supplementary Data Sheets. The Molecular Atlas is the first step to providing a resource for the detailed data-driven reconstruction and simulation of the molecular processes in the brain. As more data becomes available, the Atlas will be refined and expanded.

Many previous models of brain metabolism have been simulated within the oligocellular complex known as the neuro-glia-vasculature ensemble, or NGV (Aubert and Costalat, 2005; Cloutier et al., 2009; Jolivet et al., 2015; Calvetti et al., 2018; Coggan et al., 2018) with a significant number of parameters which still undergo numerical optimization and are not purely data-driven. Most current models of brain metabolism merely expand previous models with some extra reactions. This strategy can lead to the propagation of inaccuracies or inadequate representations. However, these flaws can be significantly reduced with a data-driven bottom-up approach in modeling and simulation studies.

It is known that many diseases include variations of molecular levels (DeBerardinis and Thompson, 2012). The Brain Molecular Atlas can help identify novel marker proteins and metabolites for various brain regions and cell types, as knowledge of biologically plausible levels of molecules is an important control in disease biomarkers research. Notably, the Molecular Atlas includes some data on AD and EAE along with corresponding healthy controls data. However, as this data is still limited, the Molecular Atlas should be considered as a prototype which will be further refined to mitigate possible confounding factors described above.

By combining AMCs with biochemical networks one can better study any aspect of their function, including the optimality of pathways, effective enzyme activity and inhibition by metabolites (Alam et al., 2017). In this way, AMC-based models will increase the power of biochemical simulations and provide the foundation for a leap forward in our understanding of metabolic networks and their roles in brain function.

## Data Availability Statement

The original contributions presented in the study are included in the article/Supplementary Material, further inquiries can be directed to the corresponding author/s.

## Author Contributions

PS performed the analyses. JC, HM, and DK provided scientific guidance. All authors contributed to study design and writing the manuscript.

## Conflict of Interest

The authors declare that the research was conducted in the absence of any commercial or financial relationships that could be construed as a potential conflict of interest.

## Publisher’s Note

All claims expressed in this article are solely those of the authors and do not necessarily represent those of their affiliated organizations, or those of the publisher, the editors and the reviewers. Any product that may be evaluated in this article, or claim that may be made by its manufacturer, is not guaranteed or endorsed by the publisher.

## Footnotes

1
http://www.informatics.jax.org/vocab/gene_ontology/

2
https://cran.r-project.org/web/packages/RISmed/RISmed.pdf

## References

[B1] Alam M. T. Olin-Sandoval V. Stincone A. Keller M. A. Zelezniak A. Luisi B. F. (2017). The self-inhibitory nature of metabolic networks and its alleviation through compartmentalization. *Nat. Commun.* 8:16018. 10.1038/ncomms16018 28691704 PMC5508129

[B2] Anand S. Mukherjee K. Padmanabhan P. (2020). An insight to flux-balance analysis for biochemical networks. *Biotechnol. Genet. Eng. Rev.* 36 32–55. 10.1080/02648725.2020.1847440 33292061

[B3] Anderson P. J. Wright B. E. (1980). Kinetic models of glycogen metabolism in normal rat liver, morris Hepatom 7787 and host liver. *Int. J. Biochem.* 12 361–369. 10.1016/0020-711X(80)90115-9 6774901

[B4] Angelidis I. Simon L. M. Fernandez I. E. Strunz M. Mayr C. H. Greiffo F. R. (2019). An atlas of the aging lung mapped by single cell transcriptomics and deep tissue proteomics. *Nat. Commun.* 10:963. 10.1038/s41467-019-08831-9 30814501 PMC6393476

[B5] Ankney J. A. Muneer A. Chen X. (2018). Relative and absolute quantitation in mass spectrometry–based proteomics. *Ann. Rev. Anal. Chem.* 11 49–77. 10.1146/annurev-anchem-061516-045357 29894226

[B6] Ashburner M. Ball C. A. Blake J. A. Botstein D. Butler H. Cherry J. M. (2000). Gene ontology: tool for the unification of biology. *Nat. Genet.* 25 25–29. 10.1038/75556 10802651 PMC3037419

[B7] Aubert A. Costalat R. (2005). Interaction between astrocytes and neurons studied using a mathematical model of compartmentalized energy metabolism. *J. Cereb. Blood Flow Metab.* 25 1476–1490. 10.1038/sj.jcbfm.9600144 15931164

[B8] Baeza-Lehnert F. Saab A. S. Gutiérrez R. Larenas V. Díaz E. Horn M. (2019). Non-canonical control of neuronal energy status by the Na+ pump. *Cell Metab.* 29 668–680e4. 10.1016/j.cmet.2018.11.005 30527744

[B9] Bai B. Wang X. Li Y. Chen P.-C. Yu K. Dey K. K. (2020). Deep multilayer brain proteomics identifies molecular networks in Alzheimer’s disease progression. *Neuron* 105 975–991.e7. 10.1016/j.neuron.2019.12.015 31926610 PMC7318843

[B10] Bantscheff M. Schirle M. Sweetman G. Rick J. Kuster B. (2007). Quantitative mass spectrometry in proteomics: a critical review. *Anal. Bioanal. Chem.* 389 1017–1031. 10.1007/s00216-007-1486-6 17668192

[B11] Barros L. F. San Martín A. Ruminot I. Sandoval P. Y. Fernández-Moncada I. Baeza-Lehnert F. (2017). Near-critical GLUT1 and neurodegeneration: glucose transport and neurodegeneration. *J. Neurosci. Res.* 95, 2267–2274. 10.1002/jnr.23998 28150866

[B12] Berndt N. Kann O. Holzhütter H.-G. (2015). Physiology-based kinetic modeling of neuronal energy metabolism unravels the molecular basis of NAD(P)H fluorescence transients. *J. Cereb. Blood Flow Metab.* 35 1494–1506. 10.1038/jcbfm.2015.70 25899300 PMC4640339

[B13] Borbély É Scheich B. Helyes Z. (2013). Neuropeptides in learning and memory. *Neuropeptides* 47 439–450. 10.1016/j.npep.2013.10.012 24210137

[B14] Braschi B. Denny P. Gray K. Jones T. Seal R. Tweedie S. (2019). Genenames.org: the HGNC and VGNC resources in 2019. *Nucleic Acids Res.* 47 D786–D792. 10.1093/nar/gky930 30304474 PMC6324057

[B15] Breckels L. M. Holden S. B. Wojnar D. Mulvey C. M. Christoforou A. Groen A. (2016). Learning from heterogeneous data sources: an application in spatial proteomics. *PLoS Comput. Biol.* 12:e1004920. 10.1371/journal.pcbi.1004920 27175778 PMC4866734

[B16] Brown M. B. Forsythe A. B. (1974). Robust tests for the equality of variances. *J. Am. Statist. Assoc.* 69 364–367. 10.1080/01621459.1974.10482955

[B17] Bult C. J. Blake J. A. Smith C. L. Kadin J. A. Richardson J. E. The Mouse Genome Database Group (2019). Mouse genome database (MGD) 2019. *Nucleic Acids Res.* 47 D801–D806. 10.1093/nar/gky1056 30407599 PMC6323923

[B18] Byrne J. H. Heidelberger R. Waxham M. N. (2014). *From Molecules to Networks: An Introduction to Cellular and Molecular Neuroscience*, 3rd Edn. Amsterdam: Elsevier/AP.

[B19] Cahoy J. D. Emery B. Kaushal A. Foo L. C. Zamanian J. L. Christopherson K. S. (2008). A transcriptome database for astrocytes, neurons, and oligodendrocytes: a new resource for understanding brain development and function. *J. Neurosci.* 28 264–278. 10.1523/JNEUROSCI.4178-07.2008 18171944 PMC6671143

[B20] Çakir T. Alsan S. Saybaşili H. Akin A. Ülgen K. Ö (2007). Reconstruction and flux analysis of coupling between metabolic pathways of astrocytes and neurons: application to cerebral hypoxia. *Theor. Biol. Med. Model* 4 48. 10.1186/1742-4682-4-48 18070347 PMC2246127

[B21] Calvetti D. Capo Rangel G. Gerardo Giorda L. Somersalo E. (2018). A computational model integrating brain electrophysiology and metabolism highlights the key role of extracellular potassium and oxygen. *J. Theor. Biol.* 446 238–258. 10.1016/j.jtbi.2018.02.029 29530764

[B22] Carlyle B. C. Kitchen R. R. Kanyo J. E. Voss E. Z. Pletikos M. Sousa A. M. M. (2017). A multiregional proteomic survey of the postnatal human brain. *Nat. Neurosci.* 20 1787–1795. 10.1038/s41593-017-0011-2 29184206 PMC5894337

[B23] Castillo E. Leon J. Mazzei G. Abolhassani N. Haruyama N. Saito T. (2017). Comparative profiling of cortical gene expression in Alzheimer’s disease patients and mouse models demonstrates a link between amyloidosis and neuroinflammation. *Sci. Rep.* 7:17762. 10.1038/s41598-017-17999-3 29259249 PMC5736730

[B24] Chelius D. Bondarenko P. V. (2002). Quantitative profiling of proteins in complex mixtures using liquid chromatography and mass spectrometry. *J. Proteome Res.* 1 317–323. 10.1021/pr025517j 12645887

[B25] Chen J. Hou W. Han B. Liu G. Gong J. Li Y. (2016). Target-based metabolomics for the quantitative measurement of 37 pathway metabolites in rat brain and serum using hydrophilic interaction ultra-high-performance liquid chromatography–tandem mass spectrometry. *Anal. Bioanal. Chem.* 408 2527–2542. 10.1007/s00216-016-9352-z 26873199

[B26] Chen W. W. Freinkman E. Wang T. Birsoy K. Sabatini D. M. (2016). Absolute quantification of matrix metabolites reveals the dynamics of mitochondrial metabolism. *Cell* 166 1324–1337.e11. 10.1016/j.cell.2016.07.040 27565352 PMC5030821

[B27] Choi I.-Y. Gruetter R. (2012). *Neural Metabolism In Vivo.* Boston, MA: Springer, 10.1007/978-1-4614-1788-0

[B28] Chuang C.-F. King C.-E. Ho B.-W. Chien K.-Y. Chang Y.-C. (2018). Unbiased proteomic study of the axons of cultured rat cortical neurons. *J. Proteome Res.* 17 1953–1966. 10.1021/acs.jproteome.8b00069 29634903

[B29] Cloutier M. Bolger F. B. Lowry J. P. Wellstead P. (2009). An integrative dynamic model of brain energy metabolism using in vivo neurochemical measurements. *J. Comput. Neurosci.* 27 391–414. 10.1007/s10827-009-0152-8 19396534

[B30] Coggan J. S. Keller D. Calì C. Lehväslaiho H. Markram H. Schürmann F. (2018). Norepinephrine stimulates glycogenolysis in astrocytes to fuel neurons with lactate. *PLoS Comput. Biol.* 14:e1006392. 10.1371/journal.pcbi.1006392 30161133 PMC6160207

[B31] Cokelaer T. Pultz D. Harder L. M. Serra-Musach J. Saez-Rodriguez J. (2013). BioServices: a common Python package to access biological web services programmatically. *Bioinformatics* 29 3241–3242. 10.1093/bioinformatics/btt547 24064416 PMC3842755

[B32] Conover W. J. Johnson M. E. Johnson M. M. (1981). A comparative study of tests for homogeneity of variances, with applications to the outer continental shelf bidding data. *Technometrics* 23 351–361. 10.1080/00401706.1981.10487680

[B33] Cox J. Hein M. Y. Luber C. A. Paron I. Nagaraj N. Mann M. (2014). Accurate proteome-wide label-free quantification by delayed normalization and maximal peptide ratio extraction, termed MaxLFQ. *Mol. Cell. Proteomics* 13 2513–2526. 10.1074/mcp.M113.031591 24942700 PMC4159666

[B34] Crisan A. Munzner T. Gardy J. L. (2019). Adjutant: an R-based tool to support topic discovery for systematic and literature reviews. *Bioinformatics* 35 1070–1072. 10.1093/bioinformatics/bty722 30875428

[B35] Cruz N. F. Lasater A. Zielke H. R. Dienel G. A. (2005). Activation of astrocytes in brain of conscious rats during acoustic stimulation: acetate utilization in working brain. *J. Neurochem* 92 934–947. 10.1111/j.1471-4159.2004.02935.x 15686496

[B36] Cudalbu C. Cavassila S. Ratiney H. Beuf O. Briguet A. Graveron-Demilly D. (2005). “Metabolite concentrations of healthy mouse brain by magnetic resonance spectroscopy at 7 tesla,” in *Proceeding of the 2005 IEEE Engineering in Medicine and Biology 27th Annual Conference*, (Shanghai: IEEE), 1392–1395. 10.1109/IEMBS.2005.1616689 17282458

[B37] Davis S. Scott C. Ansorge O. Fischer R. (2019). Development of a sensitive, scalable method for spatial, cell-type-resolved proteomics of the human brain. *J. Proteome Res.* 18 1787–1795. 10.1021/acs.jproteome.8b00981 30768908 PMC6456870

[B38] De Feyter H. M. Behar K. L. Corbin Z. A. Fulbright R. K. Brown P. B. McIntyre S. (2018). Deuterium metabolic imaging (DMI) for MRI-based 3D mapping of metabolism in vivo. *Sci. Adv.* 4:eaat7314. 10.1126/sciadv.aat7314 30140744 PMC6105304

[B39] DeBerardinis R. J. Thompson C. B. (2012). Cellular metabolism and disease: what do metabolic outliers teach us? *Cell* 148 1132–1144. 10.1016/j.cell.2012.02.032 22424225 PMC3337773

[B40] Desouki A. A. Jarre F. Gelius-Dietrich G. Lercher M. J. (2015). Cycle free flux: efficient removal of thermodynamically infeasible loops from flux distributions. *Bioinformatics* 31 2159–2165. 10.1093/bioinformatics/btv096 25701569

[B41] Deutsch E. W. Bandeira N. Sharma V. Perez-Riverol Y. Carver J. J. Kundu D. J. (2019). The ProteomeXchange consortium in 2020: enabling ‘big data’ approaches in proteomics. *Nucleic Acids Res.* 48, D1145–D1152. 10.1093/nar/gkz984 31686107 PMC7145525

[B42] DiNuzzo M. Giove F. Maraviglia B. Mangia S. (2017). Computational flux balance analysis predicts that stimulation of energy metabolism in astrocytes and their metabolic interactions with neurons depend on uptake of K+ rather than glutamate. *Neurochem. Res.* 42 202–216. 10.1007/s11064-016-2048-0 27628293 PMC5283516

[B43] DiResta G. R. Lee J. Arbit E. (1991). Measurement of brain tissue specific gravity using pycnometry. *J. Neurosci. Methods* 39 245–251. 10.1016/0165-0270(91)90103-7 1787744

[B44] Doncheva N. T. Morris J. H. Gorodkin J. Jensen L. J. (2019). Cytoscape string app: network analysis and visualization of proteomics data. *J. Proteome Res.* 18 623–632. 10.1021/acs.jproteome.8b00702 30450911 PMC6800166

[B45] Dowle M. Srinivasan A. (2020). *Data.Table**: Extension of ‘Data.Frame‘.* Available online at: https://CRAN.R-project.org/package=data.table (accessed December 30, 2020).

[B46] Duarte J. M. N. Gruetter R. (2013). Glutamatergic and GABAergic energy metabolism measured in the rat brain by 13 C NMR spectroscopy at 14.1 T. *J. Neurochem.* 126 579–590. 10.1111/jnc.12333 23745684

[B47] Duda P. Wójcicka O. Wiśniewski J. R. Rakus D. (2018). Global quantitative TPA-based proteomics of mouse brain structures reveals significant alterations in expression of proteins involved in neuronal plasticity during aging. *Aging* 10 1682–1697. 10.18632/aging.101501 30026405 PMC6075443

[B48] Ebrahim A. Lerman J. A. Palsson B. O. Hyduke D. R. (2013). COBRApy: COnstraints-based reconstruction and analysis for python. *BMC Syst. Biol.* 7:74. 10.1186/1752-0509-7-74 23927696 PMC3751080

[B49] Edfors F. Danielsson F. Hallström B. M. Käll L. Lundberg E. Pontén F. (2016). Gene-specific correlation of RNA and protein levels in human cells and tissues. *Mol. Syst. Biol.* 12:883. 10.15252/msb.20167144 27951527 PMC5081484

[B50] Eraslan B. Wang D. Gusic M. Prokisch H. Hallström B. M. Uhlén M. (2019). Quantification and discovery of sequence determinants of protein-per-mRNA amount in 29 human tissues. *Mol. Syst. Biol.* 15:513. 10.15252/msb.20188513 30777893 PMC6379048

[B51] Erecińska M. Silver I. A. (1994). Ions and energy in mammalian brain. *Prog. Neurobiol.* 43, 37–71. 10.1016/0301-0082(94)90015-9 7972852

[B52] Erö C. Gewaltig M.-O. Keller D. Markram H. (2018). A cell atlas for the mouse brain. *Front. Neuroinform.* 12:84. 10.3389/fninf.2018.00084 30546301 PMC6280067

[B53] Fecher C. Trovò L. Müller S. A. Snaidero N. Wettmarshausen J. Heink S. (2019). Cell-type-specific profiling of brain mitochondria reveals functional and molecular diversity. *Nat. Neurosci.* 22 1731–1742. 10.1038/s41593-019-0479-z 31501572

[B54] Fernandes M. Husi H. (2017). Establishment of a integrative multi-omics expression database CKDdb in the context of chronic kidney disease (CKD). *Sci. Rep.* 7:40367. 10.1038/srep40367 28079125 PMC5227717

[B55] Flanagan B. McDaid L. Wade J. Wong-Lin K. Harkin J. (2018). A computational study of astrocytic glutamate influence on post-synaptic neuronal excitability. *PLoS Comput. Biol.* 14:e1006040. 10.1371/journal.pcbi.1006040 29659572 PMC5919689

[B56] Fligner M. A. Killeen T. J. (1976). Distribution-free two-sample tests for scale. *J. Am. Statist. Assoc.* 71 210–213. 10.1080/01621459.1976.10481517

[B57] Fornasiero E. F. Mandad S. Wildhagen H. Alevra M. Rammner B. Keihani S. (2018). Precisely measured protein lifetimes in the mouse brain reveal differences across tissues and subcellular fractions. *Nat. Commun.* 9:4230. 10.1038/s41467-018-06519-0 30315172 PMC6185916

[B58] Gavai A. K. Supandi F. Hettling H. Murrell P. Leunissen J. A. M. van Beek J. H. G. M. (2015). Using bioconductor package BiGGR for metabolic flux estimation based on gene expression changes in brain. *PLoS One* 10:e0119016. 10.1371/journal.pone.0119016 25806817 PMC4373785

[B59] Geiger T. Velic A. Macek B. Lundberg E. Kampf C. Nagaraj N. (2013). Initial quantitative proteomic map of 28 mouse tissues using the SILAC mouse. *Mol. Cell. Proteomics* 12 1709–1722. 10.1074/mcp.M112.024919 23436904 PMC3675825

[B60] Gerkau N. J. Lerchundi R. Nelson J. S. E. Lantermann M. Meyer J. Hirrlinger J. (2019). Relation between activity-induced intracellular sodium transients and ATP dynamics in mouse hippocampal neurons. *J. Physiol.* 597 5687–5705. 10.1113/JP278658 31549401

[B61] Gibson G. Blass J. P. (1976). A relation between (NAD+)/(NADH) potentials and glucose utilization in rat brain slices. *J. Biol. Chem.* 251 4127–4130. 180017

[B62] Goldbaum O. Richter-Landsberg C. (2001). Stress proteins in oligodendrocytes: differential effects of heat shock and oxidative stress: stress responses in oligodendrocytes. *J. Neurochem.* 78, 1233–1242. 10.1046/j.1471-4159.2001.00507.x 11579132

[B63] Gu Z. Eils R. Schlesner M. (2016). Complex heatmaps reveal patterns and correlations in multidimensional genomic data. *Bioinformatics* 32 2847–2849. 10.1093/bioinformatics/btw313 27207943

[B64] Guergues J. Zhang P. Liu B. Stevens S. M. (2019). Improved methodology for sensitive and rapid quantitative proteomic analysis of adult-derived mouse microglia: application to a novel in vitro mouse microglial cell model. *Proteomics* 19:1800469. 10.1002/pmic.201800469 30980500 PMC7004412

[B65] Gurobi Optimization L. L. C. (2021). *Gurobi Optimizer Reference Manual.* Available online at: https://www.gurobi.com (accessed January 24, 2021).

[B66] Hagberg A. A. Schult D. A. Swart P. J. (2008). “exploring network structure, dynamics, and function using NetworkX,” in *Proceedings of the 7th Python in Science Conference*, eds Varoquaux G. Vaught T. Millman J. (Pasadena, CA), 11–15.

[B67] Hamezah H. S. Durani L. W. Yanagisawa D. Ibrahim N. F. Aizat W. M. Bellier J. P. (2018). Proteome profiling in the hippocampus, medial prefrontal cortex, and striatum of aging rat. *Exp. Gerontol.* 111 53–64. 10.1016/j.exger.2018.07.002 29981398

[B68] Hamezah H. S. Durani L. W. Yanagisawa D. Ibrahim N. F. Aizat W. M. Makpol S. (2019). Modulation of proteome profile in AβPP/PS1 mice hippocampus, medial prefrontal cortex, and striatum by palm oil derived tocotrienol-rich fraction. *JAD* 72 229–246. 10.3233/JAD-181171 31594216 PMC6839455

[B69] Han D. Jin J. Woo J. Min H. Kim Y. (2014). Proteomic analysis of mouse astrocytes and their secretome by a combination of FASP and StageTip-based, high pH, reversed-phase fractionation. *Proteomics* 14 1604–1609. 10.1002/pmic.201300495 24753479

[B70] Harris C. R. Millman K. J. van der Walt S. J. Gommers R. Virtanen P. Cournapeau D. (2020). Array programming with NumPy. *Nature* 585 357–362. 10.1038/s41586-020-2649-2 32939066 PMC7759461

[B71] Hasan M. Min H. Rahaman K. A. Muresan A. R. Kim H. Han D. (2019). Quantitative proteome analysis of brain sub-regions and spinal cord from experimental autoimmune encephalomyelitis mice by TMT-based mass spectrometry. *Proteomics* 19:1800355. 10.1002/pmic.201800355 30724464

[B72] Heckmann D. Lloyd C. J. Mih N. Ha Y. Zielinski D. C. Haiman Z. B. (2018). Machine learning applied to enzyme turnover numbers reveals protein structural correlates and improves metabolic models. *Nat. Commun.* 9:5252. 10.1038/s41467-018-07652-6 30531987 PMC6286351

[B73] Hertz L. Rothman D. (2017). Glutamine-glutamate cycle flux is similar in cultured astrocytes and brain and both glutamate production and oxidation are mainly catalyzed by aspartate aminotransferase. *Biology* 6:17. 10.3390/biology6010017 28245547 PMC5372010

[B74] Ho B. Baryshnikova A. Brown G. W. (2018). Unification of protein abundance datasets yields a quantitative saccharomyces cerevisiae proteome. *Cell Syst.* 6 192–205.e3. 10.1016/j.cels.2017.12.004 29361465

[B75] Hökfelt T. Broberger C. Xu Z.-Q. D. Sergeyev V. Ubink R. Diez M. (2000). Neuropeptidesan overview. *Neuropharmacology* 39 1337–1356. 10.1016/S0028-3908(00)00010-1 10818251

[B76] Hosp F. Gutiérrez-Ángel S. Schaefer M. H. Cox J. Meissner F. Hipp M. S. (2017). Spatiotemporal proteomic profiling of Huntington’s disease inclusions reveals widespread loss of protein function. *Cell Rep.* 21 2291–2303. 10.1016/j.celrep.2017.10.097 29166617 PMC5714591

[B77] Hounkpe B. W. Chenou F. de Lima F. De Paula E. V. (2021). HRT Atlas v1.0 database: redefining human and mouse housekeeping genes and candidate reference transcripts by mining massive RNA-seq datasets. *Nucleic Acids Res.* 49 D947–D955. 10.1093/nar/gkaa609 32663312 PMC7778946

[B78] Hrabetova S. Cognet L. Rusakov D. A. Nägerl U. V. (2018). Unveiling the extracellular space of the brain: from super-resolved microstructure to in vivo function. *J. Neurosci.* 38 9355–9363. 10.1523/JNEUROSCI.1664-18.2018 30381427 PMC6706003

[B79] Hunter J. D. (2007). Matplotlib: a 2D graphics environment. *Comput. Sci. Eng.* 9 90–95. 10.1109/MCSE.2007.55

[B80] Itzhak D. N. Davies C. Tyanova S. Mishra A. Williamson J. Antrobus R. (2017). A mass spectrometry-based approach for mapping protein subcellular localization reveals the spatial proteome of mouse primary neurons. *Cell Rep.* 20 2706–2718. 10.1016/j.celrep.2017.08.063 28903049 PMC5775508

[B81] Jean Beltran P. M. Mathias R. A. Cristea I. M. (2016). A portrait of the human organelle proteome in space and time during cytomegalovirus infection. *Cell Syst.* 3 361–373.e6. 10.1016/j.cels.2016.08.012 27641956 PMC5083158

[B82] Jolivet R. Coggan J. S. Allaman I. Magistretti P. J. (2015). Multi-timescale modeling of activity-dependent metabolic coupling in the neuron-glia-vasculature ensemble. *PLoS Comput. Biol.* 11:e1004036. 10.1371/journal.pcbi.1004036 25719367 PMC4342167

[B83] Kauffman F. C. Brown J. G. Passonneau J. V. Lowry O. H. (1969). Effects of changes in brain metabolism on levels of pentose phosphate pathway intermediates. *J. Biol. Chem.* 244 3647–3653. 5794230

[B84] Keep R. F. Hua Y. Xi G. (2012). Brain water content: a misunderstood measurement? *Transl. Stroke Res.* 3 263–265. 10.1007/s12975-012-0152-2 22888371 PMC3413327

[B85] Kim S. Chen J. Cheng T. Gindulyte A. He J. He S. (2019). PubChem 2019 update: improved access to chemical data. *Nucleic Acids Res.* 47 D1102–D1109. 10.1093/nar/gky1033 30371825 PMC6324075

[B86] Kim T.-H. Choi J. Kim H.-G. Kim H. R. (2014). Quantification of neurotransmitters in mouse brain tissue by using liquid chromatography coupled electrospray tandem mass spectrometry. *J. Anal. Methods Chem.* 2014 1–11. 10.1155/2014/506870 25258696 PMC4166658

[B87] King Z. A. Dräger A. Ebrahim A. Sonnenschein N. Lewis N. E. Palsson B. O. (2015). Escher: a web application for building, sharing, and embedding data-rich visualizations of biological pathways. *PLoS Comput. Biol.* 11:e1004321. 10.1371/journal.pcbi.1004321 26313928 PMC4552468

[B88] Kjell J. Fischer-Sternjak J. Thompson A. J. Friess C. Sticco M. J. Salinas F. (2020). Defining the adult neural stem cell niche proteome identifies key regulators of adult neurogenesis. *Cell Stem. Cell.* 26 277–293.e8. 10.1016/j.stem.2020.01.002 32032526 PMC7005820

[B89] Kleinridders A. Ferris H. A. Reyzer M. L. Rath M. Soto M. Manier M. L. (2018). Regional differences in brain glucose metabolism determined by imaging mass spectrometry. *Mol. Metab.* 12, 113–121. 10.1016/j.molmet.2018.03.013 29681509 PMC6001904

[B90] Köhler S. Schmidt H. Fülle P. Hirrlinger J. Winkler U. (2020). A dual nanosensor approach to determine the cytosolic concentration of ATP in astrocytes. *Front. Cell. Neurosci.* 14:565921. 10.3389/fncel.2020.565921 33192312 PMC7530325

[B91] Krijthe J. H. (2015). *Rtsne: T-Distributed Stochastic Neighbor Embedding using Barnes-Hut Implementation.* Available online at: https://github.com/jkrijthe/Rtsne. (accessed November 10, 2018).

[B92] Krogager T. P. Ernst R. J. Elliott T. S. Calo L. Beránek V. Ciabatti E. (2018). Labeling and identifying cell-specific proteomes in the mouse brain. *Nat. Biotechnol.* 36 156–159. 10.1038/nbt.4056 29251727 PMC6010150

[B93] Kulak A. Duarte J. M. N. Do K. Q. Gruetter R. (2010). Neurochemical profile of the developing mouse cortex determined by in vivo1H NMR spectroscopy at 14.1 T and the effect of recurrent anaesthesia: development of mouse cortical neurochemical profile. *J. Neurochem.* 115 1466–1477. 10.1111/j.1471-4159.2010.07051.x 20946416

[B94] Lee J. V. Carrer A. Shah S. Snyder N. W. Wei S. Venneti S. (2014). Akt-dependent metabolic reprogramming regulates tumor cell histone acetylation. *Cell Metab.* 20 306–319. 10.1016/j.cmet.2014.06.004 24998913 PMC4151270

[B95] Lenz K. M. Nelson L. H. (2018). Microglia and beyond: innate immune cells as regulators of brain development and behavioral function. *Front. Immunol.* 9:698. 10.3389/fimmu.2018.00698 29706957 PMC5908908

[B96] Levene H. (1960). “Robust tests for equality of variances,” in *Contributions to Probability and Statistic: Essays in Honor of Harold Hotelling*, eds Olkin I. Ghurye S. G. Hoeffding W. Madow W. G. Mann H. B. (Chicago, IL: Stanford University Press), 278–292.

[B97] Lewis N. E. Schramm G. Bordbar A. Schellenberger J. Andersen M. P. Cheng J. K. (2010). Large-scale in silico modeling of metabolic interactions between cell types in the human brain. *Nat. Biotechnol.* 28 1279–1285. 10.1038/nbt.1711 21102456 PMC3140076

[B98] Li J. J. Chew G.-L. Biggin M. D. (2017). Quantitating translational control: mRNA abundance-dependent and independent contributions and the mRNA sequences that specify them. *Nucleic Acids Res.* 45 11821–11836. 10.1093/nar/gkx898 29040683 PMC5714229

[B99] Liu X. Cooper D. E. Cluntun A. A. Warmoes M. O. Zhao S. Reid M. A. (2018). Acetate production from glucose and coupling to mitochondrial metabolism in mammals. *Cell* 175 502–513.e13. 10.1016/j.cell.2018.08.040 30245009 PMC6173642

[B100] Lularevic M. Racher A. J. Jaques C. Kiparissides A. (2019). Improving the accuracy of flux balance analysis through the implementation of carbon availability constraints for intracellular reactions. *Biotechnol. Bio.* 116 2339–2352. 10.1002/bit.27025 31112296

[B101] Lundberg E. Borner G. H. H. (2019). Spatial proteomics: a powerful discovery tool for cell biology. *Nat. Rev. Mol. Cell. Biol.* 20 285–302. 10.1038/s41580-018-0094-y 30659282

[B102] Lust W. D. Pundik S. Zechel J. Zhou Y. Buczek M. Selman W. R. (2003). Changing metabolic and energy profiles in fetal, neonatal, and adult rat brain. *Metab. Brain Dis.* 18 195–206. 10.1023/a:1025503115837 14567470

[B103] Magistretti P. J. Allaman I. (2015). A cellular perspective on brain energy metabolism and functional imaging. *Neuron* 86, 883–901. 10.1016/j.neuron.2015.03.035 25996133

[B104] Maglott D. Ostell J. Pruitt K. D. Tatusova T. (2011). Entrez gene: gene-centered information at NCBI. *Nucleic Acids Res.* 39 D52–D57. 10.1093/nar/gkq1237 21115458 PMC3013746

[B105] Mandad S. Rahman R.-U. Centeno T. P. Vidal R. O. Wildhagen H. Rammner B. (2018). The codon sequences predict protein lifetimes and other parameters of the protein life cycle in the mouse brain. *Sci. Rep.* 8:16913. 10.1038/s41598-018-35277-8 30443017 PMC6237891

[B106] Martín-Jiménez C. A. Salazar-Barreto D. Barreto G. E. González J. (2017). Genome-scale reconstruction of the human astrocyte metabolic network. *Front. Aging Neurosci.* 9:23. 10.3389/fnagi.2017.00023 28243200 PMC5303712

[B107] McBean G. (2017). Cysteine, glutathione, and thiol redox balance in astrocytes. *Antioxidants* 6:62. 10.3390/antiox6030062 28771170 PMC5618090

[B108] McKenna M. C. Waagepetersen H. S. Schousboe A. Sonnewald U. (2006). Neuronal and astrocytic shuttle mechanisms for cytosolic-mitochondrial transfer of reducing equivalents: current evidence and pharmacological tools. *Biochem. Pharmacol.* 71 399–407. 10.1016/j.bcp.2005.10.011 16368075

[B109] McKenzie A. T. Wang M. Hauberg M. E. Fullard J. F. Kozlenkov A. Keenan A. (2018). brain cell type specific gene expression and co-expression network architectures. *Sci. Rep.* 8:8868. 10.1038/s41598-018-27293-5 29892006 PMC5995803

[B110] McKetney J. Runde R. M. Hebert A. S. Salamat S. Roy S. Coon J. J. (2019). Proteomic atlas of the human brain in Alzheimer’s disease. *J. Proteome Res.* 18 1380–1391. 10.1021/acs.jproteome.9b00004 30735395 PMC6480317

[B111] Metelkin E. Demin O. Kovács Z. Chinopoulos C. (2009). Modeling of ATP-ADP steady-state exchange rate mediated by the adenine nucleotide translocase in isolated mitochondria: modeling of ANT. *FEBS J.* 276 6942–6955. 10.1111/j.1742-4658.2009.07394.x 19860824

[B112] Mi H. Huang X. Muruganujan A. Tang H. Mills C. Kang D. (2017). PANTHER version 11: expanded annotation data from gene ontology and reactome pathways, and data analysis tool enhancements. *Nucleic Acids Res.* 45 D183–D189. 10.1093/nar/gkw1138 27899595 PMC5210595

[B113] Milo R. (2013). What is the total number of protein molecules per cell volume? A call to rethink some published values. *BioEssays* 35 1050–1055. 10.1002/bies.201300066 24114984 PMC3910158

[B114] Milo R. Jorgensen P. Moran U. Weber G. Springer M. (2010). BioNumbers–the database of key numbers in molecular and cell biology. *Nucleic Acids Res.* 38 D750–D753. 10.1093/nar/gkp889 19854939 PMC2808940

[B115] Mogilevskaya E. Demin O. Goryanin I. (2006). Kinetic model of mitochondrial krebs cycle: unraveling the mechanism of salicylate hepatotoxic effects. *J. Biol. Phys.* 32 245–271. 10.1007/s10867-006-9015-y 19669466 PMC2651525

[B116] Muraleedharan R. Gawali M. V. Tiwari D. Sukumaran A. Oatman N. Anderson J. (2020). AMPK-regulated astrocytic lactate shuttle plays a non-cell-autonomous role in neuronal survival. *Cell Rep.* 32:108092. 10.1016/j.celrep.2020.108092 32877674 PMC7531170

[B117] Nakayama Y. Kinoshita A. Tomita M. (2005). Dynamic simulation of red blood cell metabolism and its application to the analysis of a pathological condition. *Theor. Biol. Med. Model* 2:18. 10.1186/1742-4682-2-18 15882454 PMC1142344

[B118] Navarro J. F. Croteau D. L. Jurek A. Andrusivova Z. Yang B. Wang Y. (2020). Spatial transcriptomics reveals genes associated with dysregulated mitochondrial functions and stress signaling in Alzheimer disease. *iScience* 23:101556. 10.1016/j.isci.2020.101556 33083725 PMC7522123

[B119] Neves A. Costalat R. Pellerin L. (2012). Determinants of brain cell metabolic phenotypes and energy substrate utilization unraveled with a modeling approach. *PLoS Comput. Biol.* 8:e1002686. 10.1371/journal.pcbi.1002686 23028284 PMC3441424

[B120] Ning K. Fermin D. Nesvizhskii A. I. (2012). Comparative analysis of different label-free mass spectrometry based protein abundance estimates and their correlation with RNA-Seq gene expression data. *J. Proteome Res.* 11 2261–2271. 10.1021/pr201052x 22329341 PMC3744887

[B121] Noor E. Flamholz A. Bar-Even A. Davidi D. Milo R. Liebermeister W. (2016). The protein cost of metabolic fluxes: prediction from enzymatic rate laws and cost minimization. *PLoS Comput. Biol.* 12:e1005167. 10.1371/journal.pcbi.1005167 27812109 PMC5094713

[B122] O’Brien E. J. Palsson B. O. (2015). Computing the functional proteome: recent progress and future prospects for genome-scale models. *Curr. Opin. Biotechnol.* 34 125–134. 10.1016/j.copbio.2014.12.017 25576845 PMC4495013

[B123] Ohse S. Boerries M. Busch H. (2019). Blind normalization of public high-throughput databases. *PeerJ Comput. Sci.* 5:e231. 10.7717/peerj-cs.231 33816884 PMC7924423

[B124] Ong S.-E. Blagoev B. Kratchmarova I. Kristensen D. B. Steen H. Pandey A. (2002). Stable isotope labeling by amino acids in cell culture, SILAC, as a simple and accurate approach to expression proteomics. *Mol. Cell. Proteom.* 1 376–386. 10.1074/mcp.M200025-MCP200 12118079

[B125] Orth J. D. Thiele I. Palsson B. Ø (2010). What is flux balance analysis? *Nat. Biotechnol.* 28 245–248. 10.1038/nbt.1614 20212490 PMC3108565

[B126] Palm D. C. Rohwer J. M. Hofmeyr J.-H. S. (2013). Regulation of glycogen synthase from mammalian skeletal musclea unifying view of allosteric and covalent regulation. *FEBS J.* 280 2–27. 10.1111/febs.12059 23134486

[B127] Pandey V. Hadadi N. Hatzimanikatis V. (2019). Enhanced flux prediction by integrating relative expression and relative metabolite abundance into thermodynamically consistent metabolic models. *PLoS Comput. Biol.* 15:e1007036. 10.1371/journal.pcbi.1007036 31083653 PMC6532942

[B128] Patel A. B. de Graaf R. A. Mason G. F. Kanamatsu T. Rothman D. L. Shulman R. G. (2004). Glutamatergic neurotransmission and neuronal glucose oxidation are coupled during intense neuronal activation. *J. Cereb. Blood Flow Metab.* 24 972–985. 10.1097/01.WCB.0000126234.16188.71 15356418

[B129] Pouwels P. J. Frahm J. (1998). Regional metabolite concentrations in human brain as determined by quantitative localized proton MRS. *Magn. Reson. Med.* 39 53–60. 10.1002/mrm.1910390110 9438437

[B130] Reback J. McKinney W. Jbrockmendel, Van Den Bossche J. Augspurger T. Cloud P. (2021). *Pandas-Dev/**Pandas: Pandas 1.3.3.* Zenodo, 10.5281/ZENODO.3509134

[B131] Remes P. M. Yip P. MacCoss M. J. (2020). Highly multiplex targeted proteomics enabled by real-time chromatographic alignment. *Anal. Chem.* 92 11809–11817. 10.1021/acs.analchem.0c02075 32867497 PMC7757911

[B132] Ritchie M. E. Phipson B. Wu D. Hu Y. Law C. W. Shi W. (2015). limma powers differential expression analyses for RNA-sequencing and microarray studies. *Nucleic Acids Res.* 43 e47. 10.1093/nar/gkv007 25605792 PMC4402510

[B133] Robinson M. B. Jackson J. G. (2016). Astroglial glutamate transporters coordinate excitatory signaling and brain energetics. *Neurochem. Int.* 98 56–71. 10.1016/j.neuint.2016.03.014 27013346 PMC4969184

[B134] Robinson M. D. McCarthy D. J. Smyth G. K. (2010). edgeR: a Bioconductor package for differential expression analysis of digital gene expression data. *Bioinformatics* 26 139–140. 10.1093/bioinformatics/btp616 19910308 PMC2796818

[B135] Ronowska A. Szutowicz A. Bielarczyk H. Gul-Hinc S. Klimaszewska-Łata J. Dyś A. (2018). The regulatory effects of acetyl-CoA distribution in the healthy and diseased brain. *Front. Cell. Neurosci.* 12:169. 10.3389/fncel.2018.00169 30050410 PMC6052899

[B136] Sabate L. Franco R. Canela E. I. Centelles J. J. Cascante M. (1995). A model of the pentose phosphate pathway in rat liver cells. *Mol. Cell Biochem.* 142 9–17. 10.1007/BF00928908 7753046

[B137] Sánchez B. J. Zhang C. Nilsson A. Lahtvee P. Kerkhoven E. J. Nielsen J. (2017). Improving the phenotype predictions of a yeast genome-scale metabolic model by incorporating enzymatic constraints. *Mol. Syst. Biol.* 13:935. 10.15252/msb.20167411 28779005 PMC5572397

[B138] Santuy A. Turégano-López M. Rodríguez J. R. Alonso-Nanclares L. DeFelipe J. Merchán-Pérez A. (2018). A quantitative study on the distribution of mitochondria in the neuropil of the juvenile rat somatosensory cortex. *Cerebral. Cortex* 28 3673–3684. 10.1093/cercor/bhy159 30060007 PMC6132283

[B139] Schauberger P. Walker A. (2020). *Openxlsx: Read, Write and Edit XLSX Files.* Available online at: https://CRAN.R-project.org/package=openxlsx (accessed January 27, 2020).

[B140] Schellenberger J. Lewis N. E. Palsson B. Ø (2011). Elimination of thermodynamically infeasible loops in steady-state metabolic models. *Biophys. J.* 100 544–553. 10.1016/j.bpj.2010.12.3707 21281568 PMC3030201

[B141] Schwanhäusser B. Busse D. Li N. Dittmar G. Schuchhardt J. Wolf J. (2011). Global quantification of mammalian gene expression control. *Nature* 473 337–342. 10.1038/nature10098 21593866

[B142] Schwarz D. S. Blower M. D. (2016). The endoplasmic reticulum: structure, function and response to cellular signaling. *Cell. Mol. Life Sci.* 73 79–94. 10.1007/s00018-015-2052-6 26433683 PMC4700099

[B143] Shannon P. (2003). Cytoscape: a software environment for integrated models of biomolecular interaction networks. *Geno. Res.* 13 2498–2504. 10.1101/gr.1239303 14597658 PMC403769

[B144] Sharma K. Schmitt S. Bergner C. G. Tyanova S. Kannaiyan N. Manrique-Hoyos N. (2015). Cell type– and brain region–resolved mouse brain proteome. *Nat. Neurosci.* 18 1819–1831. 10.1038/nn.4160 26523646 PMC7116867

[B145] Shestov A. A. Valette J. Uğurbil K. Henry P.-G. (2007). On the reliability of13C metabolic modeling with two-compartment neuronal-glial models. *J. Neurosci. Res.* 85 3294–3303. 10.1002/jnr.21269 17393498

[B146] Sigurdsson M. I Jamshidi N. Steingrimsson E. Thiele I. Palsson B. Ø (2010). A detailed genome-wide reconstruction of mouse metabolism based on human recon 1. *BMC Syst. Biol.* 4:140. 10.1186/1752-0509-4-140 20959003 PMC2978158

[B147] Silge J. Robinson D. (2016). tidytext: text mining and analysis using tidy data principles in R. *JOSS* 1:37. 10.21105/joss.00037

[B148] Silva G. M. Vogel C. (2016). Quantifying gene expression: the importance of being subtle. *Mol. Syst. Biol.* 12:885. 10.15252/msb.20167325 27951528 PMC5081482

[B149] Sjödin S. Öhrfelt A. Brinkmalm G. Zetterberg H. Blennow K. Brinkmalm A. (2016). Targeting LAMP2 in human cerebrospinal fluid with a combination of immunopurification and high resolution parallel reaction monitoring mass spectrometry. *Clin. Proteom.* 13:4. 10.1186/s12014-016-9104-2 26924951 PMC4768413

[B150] Sølling H. (1979). Studies on the allosteric properties of glycogen synthase I. *Eur. J. Biochem.* 94 231–242. 10.1111/j.1432-1033.1979.tb12890.x 108102

[B151] Sugimoto M. Ikeda S. Niigata K. Tomita M. Sato H. Soga T. (2012). MMMDB: mouse multiple tissue metabolome database. *Nucleic Acids Res.* 40 D809–D814. 10.1093/nar/gkr1170 22139941 PMC3245187

[B152] Sundberg J. P. Schofield P. N. (2010). Commentary: mouse genetic nomenclature: standardization of strain, gene, and protein symbols. *Vet. Pathol.* 47 1100–1104. 10.1177/0300985810374837 20685919 PMC3039125

[B153] Supandi F. van Beek J. H. G. M. (2018). Computational prediction of changes in brain metabolic fluxes during Parkinson’s disease from mRNA expression. *PLoS One* 13:e0203687. 10.1371/journal.pone.0203687 30208076 PMC6135490

[B154] Szklarczyk D. Gable A. L. Lyon D. Junge A. Wyder S. Huerta-Cepas J. (2019). STRING v11: protein–protein association networks with increased coverage, supporting functional discovery in genome-wide experimental datasets. *Nucleic Acids Res.* 47 D607–D613. 10.1093/nar/gky1131 30476243 PMC6323986

[B155] Tange O. (2020). *GNU Parallel 20200622 (‘Floyd’).* Zenodo, 10.5281/ZENODO.3903853

[B156] Tasic B. Menon V. Nguyen T. N. Kim T. K. Jarsky T. Yao Z. (2016). Adult mouse cortical cell taxonomy revealed by single cell transcriptomics. *Nat. Neurosci.* 19 335–346. 10.1038/nn.4216 26727548 PMC4985242

[B157] Terpilowski M. (2019). scikit-posthocs: pairwise multiple comparison tests in python. *JOSS* 4:1169. 10.21105/joss.01169

[B158] The Gene Ontology Consortium (2019). The gene ontology resource: 20 years and still going strong. *Nucleic Acids Res.* 47 D330–D338. 10.1093/nar/gky1055 30395331 PMC6323945

[B159] The UniProt Consortium (2017). UniProt: the universal protein knowledgebase. *Nucleic Acids Res.* 45 D158–D169. 10.1093/nar/gkw1099 27899622 PMC5210571

[B160] Thompson A. Schäfer J. Kuhn K. Kienle S. Schwarz J. Schmidt G. (2003). Tandem Mass tags: a novel quantification strategy for comparative analysis of complex protein mixtures by MS/MS. *Anal. Chem.* 75 1895–1904. 10.1021/ac0262560 12713048

[B161] Tian M. Reed J. L. (2018). Integrating proteomic or transcriptomic data into metabolic models using linear bound flux balance analysis. *Bioinformatics* 34 3882–3888. 10.1093/bioinformatics/bty445 29878053 PMC6223374

[B162] Tillack J. Paczia N. Nöh K. Wiechert W. Noack S. (2012). Error propagation analysis for quantitative intracellular metabolomics. *Metabolites* 2 1012–1030. 10.3390/metabo2041012 24957773 PMC3901244

[B163] Tretter L. Patocs A. Chinopoulos C. (2016). Succinate, an intermediate in metabolism, signal transduction, ROS, hypoxia, and tumorigenesis. *Biochimica et Biophys. Acta (BBA)Bioenergetics* 1857 1086–1101. 10.1016/j.bbabio.2016.03.012 26971832

[B164] Tsuboi K. K. Fukunaga K. Petricciani J. C. (1969). Purification and specific kinetic properties of erythrocyte uridine diphosphate glucose pyrophosphorylase. *J. Biol. Chem.* 244 1008–1015. 5782905

[B165] Tyanova S. Temu T. Sinitcyn P. Carlson A. Hein M. Y. Geiger T. (2016). The Perseus computational platform for comprehensive analysis of (prote)omics data. *Nat. Methods* 13 731–740. 10.1038/nmeth.3901 27348712

[B166] VanderPlas J. Granger B. Heer J. Moritz D. Wongsuphasawat K. Satyanarayan A. (2018). Altair: interactive statistical visualizations for python. *JOSS* 3:1057. 10.21105/joss.01057

[B167] Virtanen P. Gommers R. Oliphant T. E. Haberland M. Reddy T. Cournapeau D. (2020). SciPy 1.0: fundamental algorithms for scientific computing in Python. *Nat. Methods* 17 261–272. 10.1038/s41592-019-0686-2 32015543 PMC7056644

[B168] Vogel C. de Sousa Abreu R. Ko D. Le S. Shapiro B. A. Burns S. C. (2010). Sequence signatures and mRNA concentration can explain two-thirds of protein abundance variation in a human cell line. *Mol. Syst. Biol.* 6:400. 10.1038/msb.2010.59 20739923 PMC2947365

[B169] Wain H. M. Bruford E. A. Lovering R. C. Lush M. J. Wright M. W. Povey S. (2002). Guidelines for human gene nomenclature. *Genomics* 79 464–470. 10.1006/geno.2002.6748 11944974

[B170] Wang M. Weiss M. Simonovic M. Haertinger G. Schrimpf S. P. Hengartner M. O. (2012). PaxDb, a database of protein abundance averages across all three domains of life. *Mol. Cell. Proteom.* 11 492–500. 10.1074/mcp.O111.014704 22535208 PMC3412977

[B171] Waskom M. Gelbart M. Botvinnik O. Ostblom J. Hobson P. Lukauskas S. (2021). *Mwaskom/Seaborn: v0.11.2.* Zenodo. 10.5281/ZENODO.592845

[B172] Wickham H. (2016). *ggplot2: Elegant Graphics for Data Analysis.* New York: Springer-Verlag.

[B173] Wickham H. (2019). *stringr: Simple, Consistent Wrappers for Common String Operations.* Available online at: https://CRAN.R-project.org/package=stringr (accessed February 10, 2019).

[B174] Wickham H. François R. Henry L. Müller K. (2021). *dplyr: A Grammar of Data Manipulation.* Available online at: https://CRAN.R-project.org/package=dplyr (accessed August 18, 2020).

[B175] Wiebenga O. T. Klauser A. M. Nagtegaal G. J. A. Schoonheim M. M. Barkhof F. Geurts J. J. G. (2014). Longitudinal absolute metabolite quantification of white and gray matter regions in healthy controls using proton MR spectroscopic imaging: longitudinal MRSI in healthy controls. *NMR Biomed.* 27 304–311. 10.1002/nbm.3063 24399803

[B176] Wild C. J. Pfannkuch M. Regan M. Horton N. J. (2011). Towards more accessible conceptions of statistical inferences [with discussion]. *J. R. Stat. Soc. Ser. A Stat. Soc.* 174, 247–295.

[B177] Wilhelm M. Schlegl J. Hahne H. Gholami A. M. Lieberenz M. Savitski M. M. (2014). Mass-spectrometry-based draft of the human proteome. *Nature* 509 582–587. 10.1038/nature13319 24870543

[B178] Wishart D. S. Feunang Y. D. Marcu A. Guo A. C. Liang K. Vázquez-Fresno R. (2018). HMDB 4.0: the human metabolome database for 2018. *Nucleic Acids Res.* 46 D608–D617. 10.1093/nar/gkx1089 29140435 PMC5753273

[B179] Wishart D. S. Jewison T. Guo A. C. Wilson M. Knox C. Liu Y. (2012). HMDB 3.0the human metabolome database in 2013. *Nucleic Acids Res.* 41 D801–D807. 10.1093/nar/gks1065 23161693 PMC3531200

[B180] Wishart D. S. Knox C. Guo A. C. Eisner R. Young N. Gautam B. (2009). HMDB: a knowledgebase for the human metabolome. *Nucleic Acids Res.* 37 D603–D610. 10.1093/nar/gkn810 18953024 PMC2686599

[B181] Wishart D. S. Tzur D. Knox C. Eisner R. Guo A. C. Young N. (2007). HMDB: the human metabolome database. *Nucleic Acids Res.* 35 D521–D526. 10.1093/nar/gkl923 17202168 PMC1899095

[B182] Wiśniewski J. R. Gizak A. Rakus D. (2015). Integrating proteomics and enzyme kinetics reveals tissue-specific types of the glycolytic and gluconeogenic pathways. *J. Proteome Res.* 14 3263–3273. 10.1021/acs.jproteome.5b00276 26080680

[B183] Wiśniewski J. R. Hein M. Y. Cox J. Mann M. (2014). A “proteomic ruler” for protein copy number and concentration estimation without spike-in standards. *Mol. Cell. Proteomics* 13 3497–3506. 10.1074/mcp.M113.037309 25225357 PMC4256500

[B184] Yu Q. Xiao H. Jedrychowski M. P. Schweppe D. K. Navarrete-Perea J. Knott J. (2020). Sample multiplexing for targeted pathway proteomics in aging mice. *Proc. Natl. Acad. Sci. U.S.A.* 117, 9723–9732. 10.1073/pnas.1919410117 32332170 PMC7211924

[B185] Zeisel A. Hochgerner H. Lönnerberg P. Johnsson A. Memic F. van der Zwan J. (2018). Molecular architecture of the mouse nervous system. *Cell* 174 999–1014.e22. 10.1016/j.cell.2018.06.021 30096314 PMC6086934

[B186] Zheng X. Chen T. Zhao A. Wang X. Xie G. Huang F. (2016). The brain metabolome of male rats across the lifespan. *Sci. Rep.* 6:24125. 10.1038/srep24125 27063670 PMC4827083

[B187] Zheng X. Kang A. Dai C. Liang Y. Xie T. Xie L. (2012). Quantitative analysis of neurochemical panel in rat brain and plasma by liquid chromatography–tandem mass spectrometry. *Anal. Chem.* 84 10044–10051. 10.1021/ac3025202 23098234

[B188] Zhu Y. Dou M. Piehowski P. D. Liang Y. Wang F. Chu R. K. (2018). Spatially resolved proteome mapping of laser capture microdissected tissue with automated sample transfer to nanodroplets. *Mol. Cell. Proteomics* 17 1864–1874. 10.1074/mcp.TIR118.000686 29941660 PMC6126383

