# Supplementary material for: A Standardized Brain Molecular Atlas: A Resource for Systems Modeling and Simulation
Source: Front Mol Neurosci. 2021 Nov 10;14:604559. doi: 10.3389/fnmol.2021.604559 (PMC8631404; doi:10.3389/fnmol.2021.604559)
Supplement: Supplementary file 8 [file Presentation_1.pdf]

Supplementary Presentation

1 Supplementary Data Columns Annotation

Data Sheets 1-4. Descriptive column names for Estimated protein concentrations. Column names: “gene_names” - original gene names as given in source data, “Uniprot” - original UniProt identifiers as given in source data, “Study” - reference publication, “Organism” - species (mouse, rat, human), “location” - brain tissue, brain region, cell type or subcellular location where the sample comes from, “Age_cat” - age category, “condition” - conditions which are being reported for given sample, “sample_id” - sample identifier in cases of multiple repetitions, “molecular_weight_kDa” - protein molecular weight (kDa), “raw_data_units” - type of data/units reported in source data set, “gene_id_final” - main aligned gene names, “Uniprot_final” - aligned UniProt identifiers (which should not be considered as main identifiers and only given for additional reference), “conc_uM” - calculated concentrations (before normalization), “log_conc_uM” - natural log transformed calculated concentrations (before normalization), “gene_id_dd” - manually resolved duplicated gene names produced in the gene alignment procedure, “log_conc_uM_medNorm” - normalized calculated concentrations (main data).

Data Sheet 5. Descriptive column names for Estimated metabolite concentrations. Columns: “Methods” - general category of data source, “Organism” - species, “age” - age as given in the source, “cellType” - cell type, “cid_pubchem” - PubChem compound identifier, “comments” - additional information (metadata or comments) for the entry, “level_units” - unified units, “level_units_ini” - units as in source data set, “level_units_iniUniformNames” - scaled units from the source data set (for example, scaled from “milli-” to “micro-”), “level_value_uM” - processed micromolar concentrations, “locLevelOfDetails” - level of details and confidence at which the data is reported (organelle, cell, tissue, estimation, review, model:secondary data), “location” - biological location, “methods details” - additional details about methods with which data was obtained, “molecule_name” - molecule name, “organism/cell culture” - organism/cell culture, ”reference” - reference to the data source, “subcellularLoc” - subcellular location (when applicable), “tissue” - brain, blood, cerebrospinal fluid, cell lines.

2 Supplementary Figures

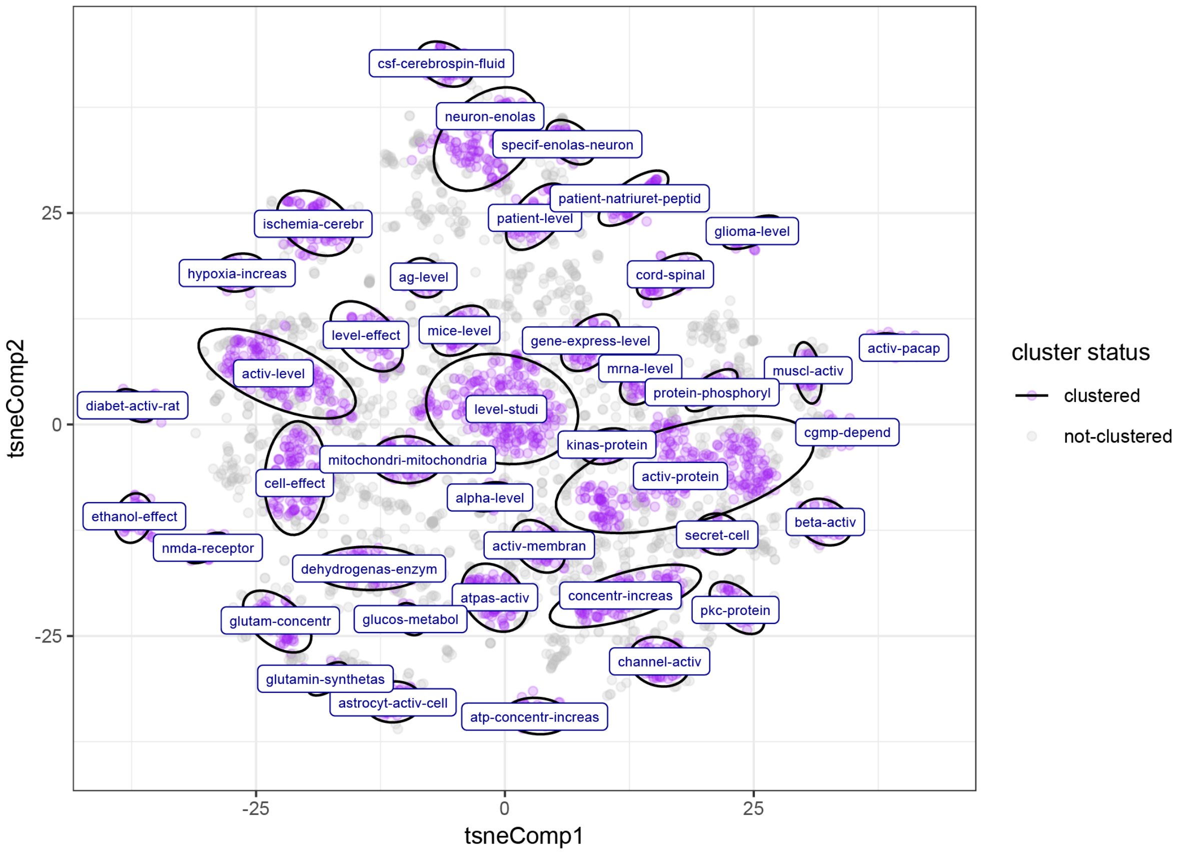

Supplementary Figure 1. Diversity of studies associated with energy metabolism of the brain and the glutamine-glutamate cycle shown through clustering of the papers related to energy metabolism and the glutamine-glutamate cycle.

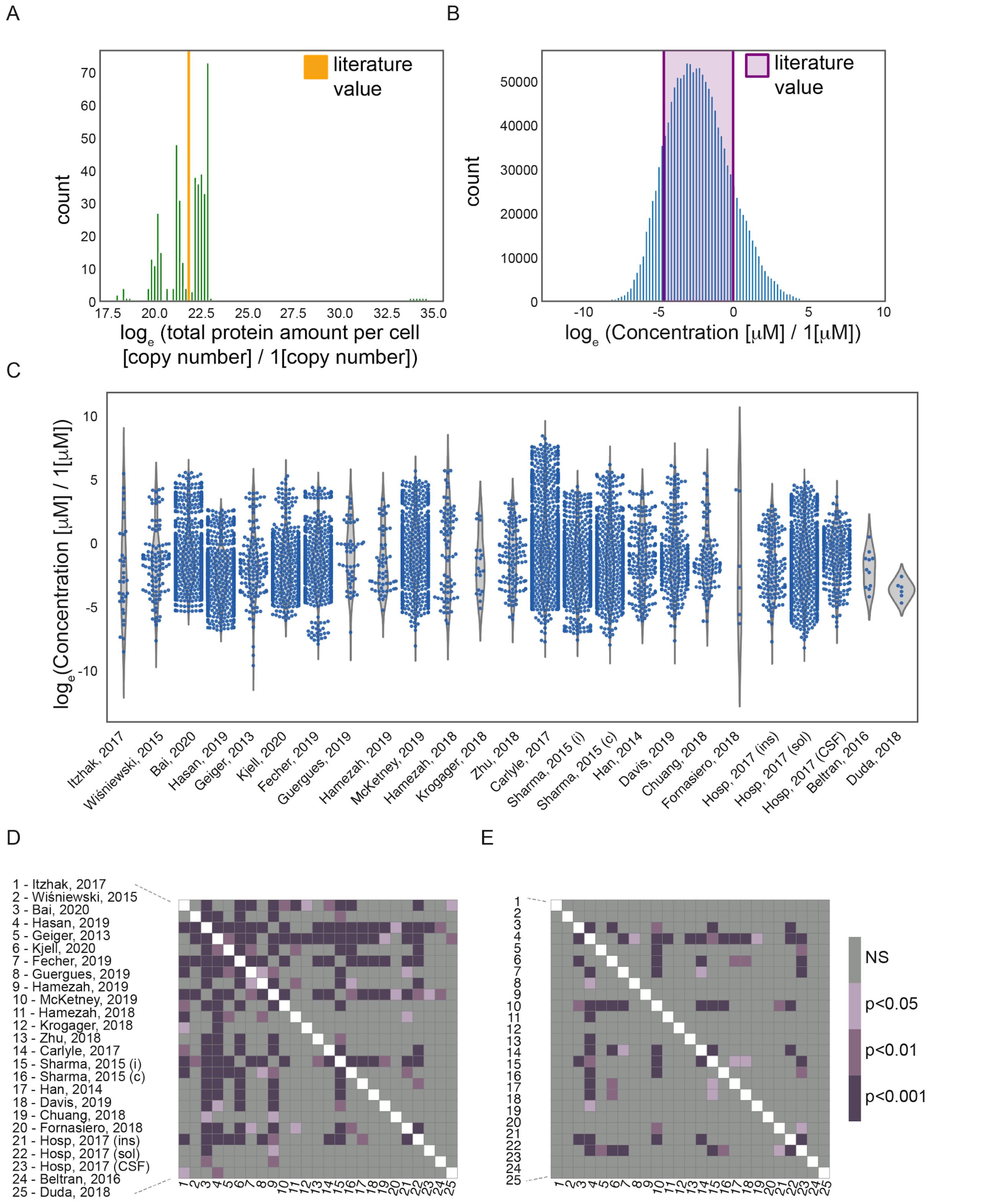

Supplementary Figure 2. Statistical evaluation for the results of protein and peptide level normalization for the experimental methods used. (A) Total protein amount per cell compared to literature. (B) Signaling proteins concentrations compared to literature. (C) Peptide concentration distributions. (D) Holm adjusted p-values from Conover post-hoc test (applied after Kruskal-Wallis Test H = 1034.55, p-value = 4.07e-203) for comparison of normalized protein concentrations from

different studies. (E) Holm adjusted p-values from Conover post-hoc test (applied after Kruskal-Wallis Test H = 442.67, p-value = 1.24e-78) for comparison of normalized concentrations of peptides from different studies. Log_e is a natural logarithm of specified data. CSF stands for cerebrospinal fluid, “ins” is “insoluble”, “sol” is “soluble”, “c” is “cultured”, and “i” is “isolated”.

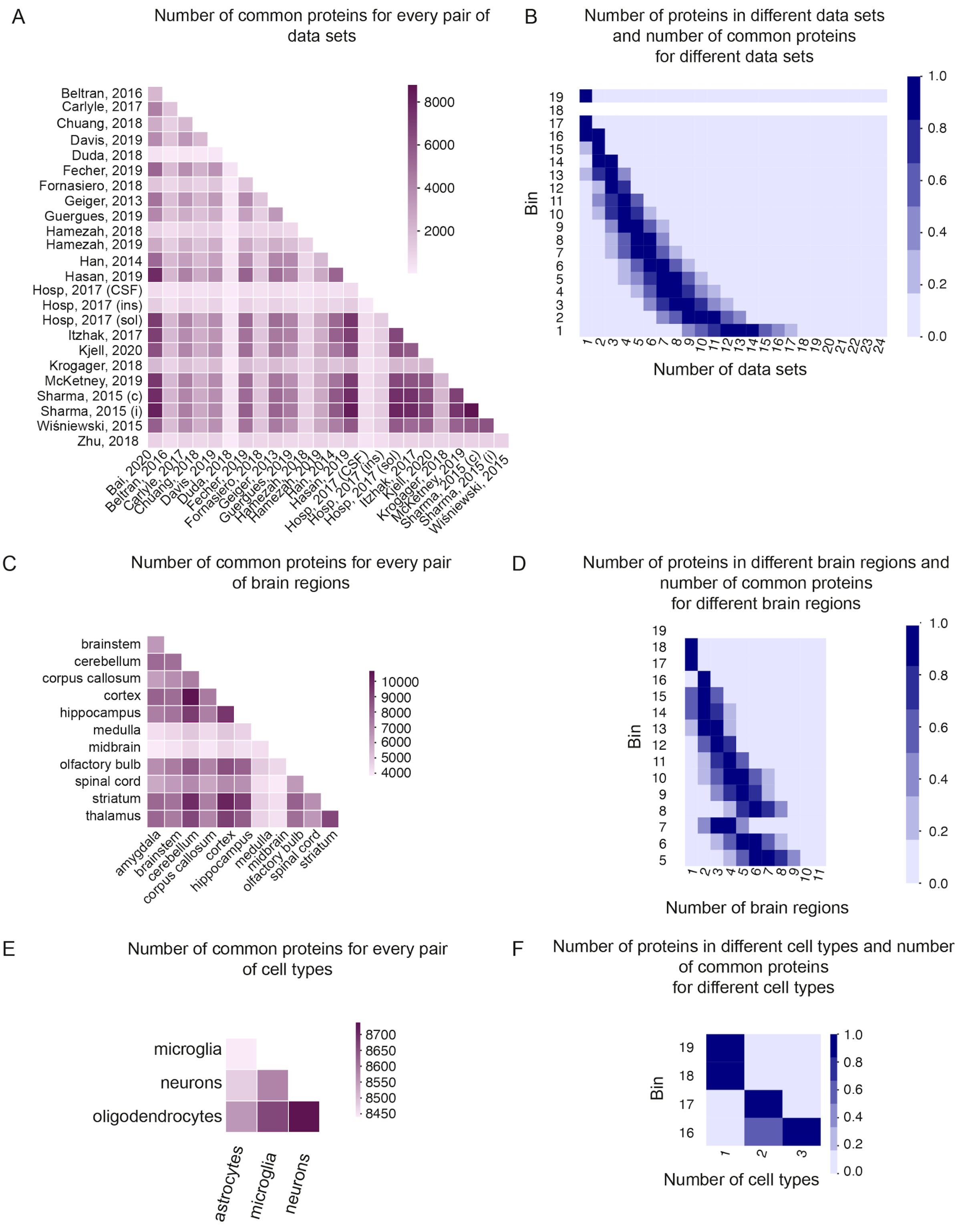

Supplementary Figure 3. Numbers of proteins with known concentrations across data sets, brain regions and cell types. (A) Pairwise combinations of data sets. (B) Number of proteins in different data sets and number of common proteins for different number of data sets combined. Bins from one to 20 correspond to the following intervals: 1) 0-550, 2) 550-1100, 3) 1100-1650, 4) 1650-2200, 5) 2200-2750, 6) 2750-3300, 7) 3300-3850, 8) 3850-4400, 9) 4400-4950, 10) 4950-5500, 11) 5500-6050, 12) 6050-6600, 13) 6600-7150, 14) 7150-7700, 15) 7700-8250, 16) 8250-8800, 17) 8800-

9350, 18) 9350-9900, 19) 9900-10450, 20) 10450-11000 of numbers of common proteins across all combinations of given number of selected data sets. For the visualization purposes a number of common proteins across different numbers of data sets were scaled by dividing by the max value which can be found across any number of data sets for every given bin. Bins with only zero values across all combinations are omitted in visualization. Pairwise combinations of brain regions (C) and cell types (E). (D,F) Number of proteins and number of common proteins for different numbers of brain regions (D) and cell types (F) combined. (D) Bins from one to 20 correspond to the following intervals: 1) 0-650, 2) 650-1300, 3) 1300-1950, 4) 1950-2600, 5) 2600-3250, 6) 3250-3900, 7) 3900-4550, 8) 4550-5200, 9) 5200-5850, 10) 5850-6500, 11) 6500-7150, 12) 7150-7800, 13) 7800-8450, 14) 8450-9100, 15) 9100-9750, 16) 9750-10400, 17) 10400-11050, 18) 11050-11700, 19) 11700-12350, 20) 12350-13000 of numbers of common proteins across all combinations of given number of selected brain regions. For the visualization purposes a number of common proteins across different numbers of brain regions were scaled by dividing by the max value which can be found across any number of brain regions for every given bin. Bins with only zero values across all combinations are omitted in visualization. (F) Bins from one to 20 correspond to the following intervals: 1) 0-500, 2) 500-1000, 3) 1000-1500, 4) 1500-2000, 5) 2000-2500, 6) 2500-3000, 7) 3000-3500, 8) 3500-4000, 9) 4000-4500, 10) 4500-5000, 11) 5000-5500, 12) 5500-6000, 13) 6000-6500, 14) 6500-7000, 15) 7000-7500, 16) 7500-8000, 17) 8000-8500, 18) 8500-9000, 19) 9000-9500, 20) 9500-10000 of numbers of common proteins across all combinations of given numbers of selected cell types. For the visualization purposes a number of common proteins across different numbers of cell types were scaled by dividing by the max value which can be found across any number of cell types for every given bin. Bins with only zero values across all combinations are omitted in visualization.

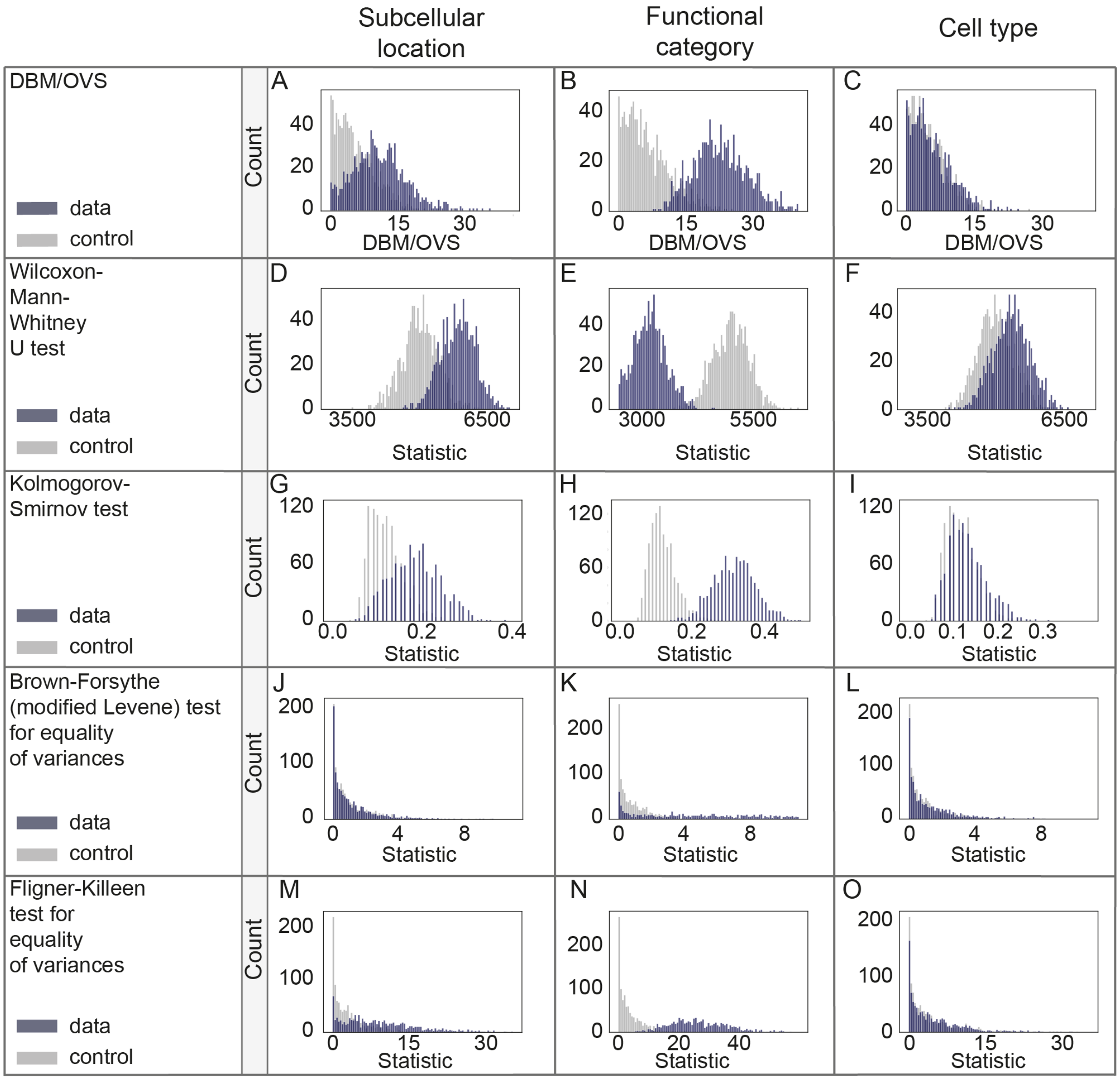

Supplementary Figure 4. Factors explaining variability of protein concentrations. (A-O) Statistical analyses in permutations with multiple (1000) resampling with sample sizes of 100. Types of analyses are named in the left panel of each row. No log-transformation was applied in this case.

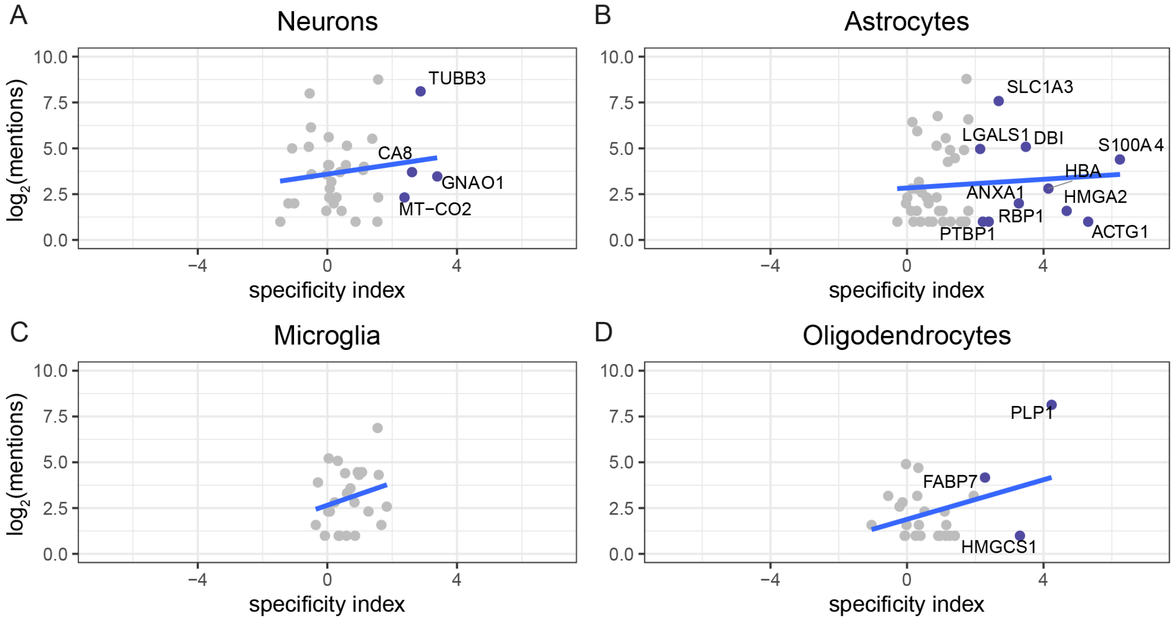

Supplementary Figure 5. Cell type specificity of proteins compared with literature associations. Protein concentrations specificity in cell types compared to association of these proteins with related locations. Genes with natural log fold specificity of their products' concentrations more than two and less than -2 are marked in purple. Difference in levels of protein in one region or cell type compared to all others is referred to as a specificity index.

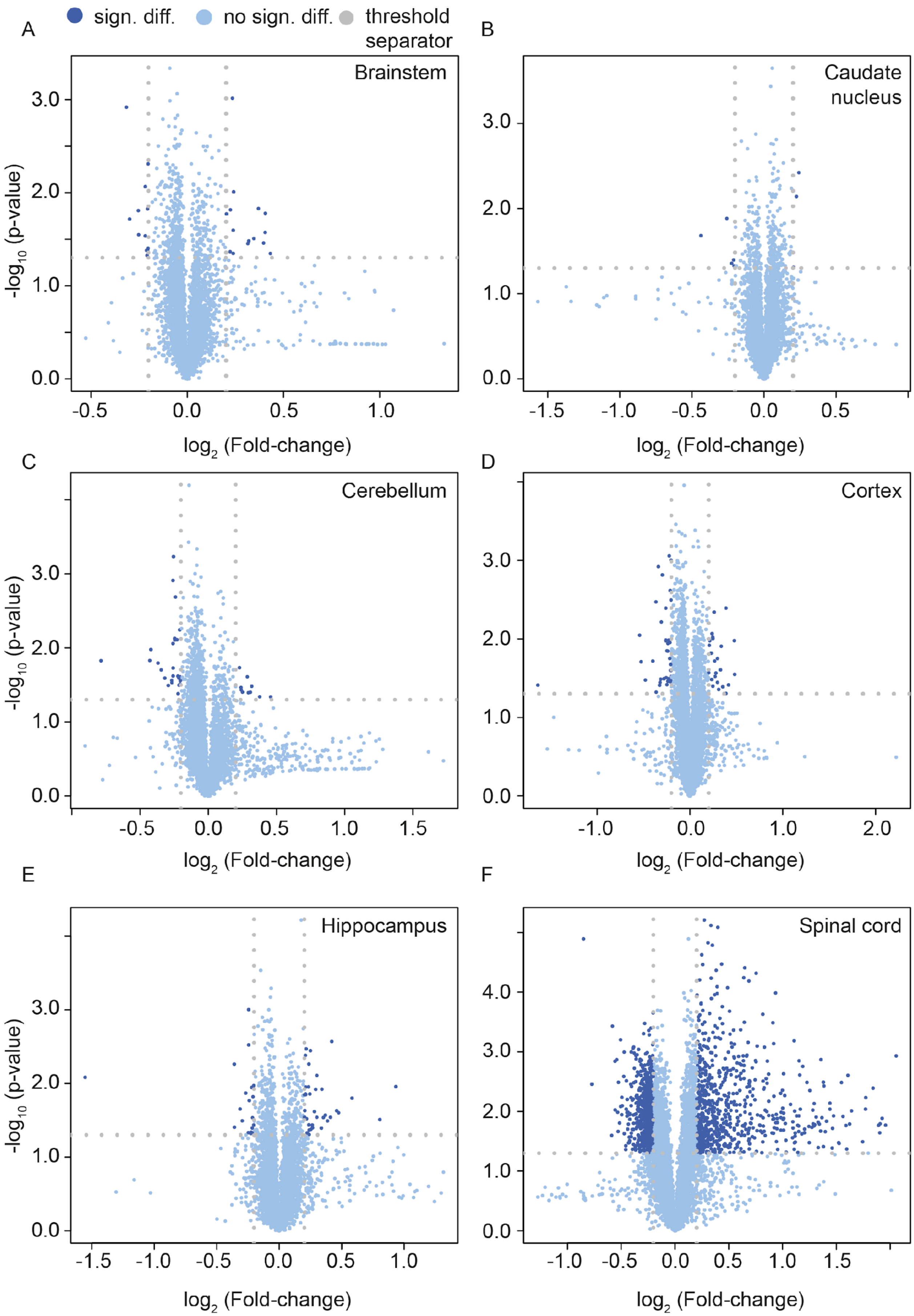

sign. diff.
no sign. diff.
threshold separator
A
Brainstem
B
Caudate nucleus
C
Cerebellum
D
Cortex
E
Hippocampus
F
Spinal cord
-log10 (p-value)
log2 (Fold-change)

Supplementary Figure 6. Volcano plots for differential protein concentrations in EAE compared to healthy states across six brain regions. (A) Brainstem. (B) Caudate nucleus (in other analyses it is combined with different striatum samples). (C) Cerebellum. (D) Cortex. (E) Hippocampus. (F) Spinal cord. Threshold separator splits significantly differentially expressed proteins from other ones following the same criteria as in (Hasan et al. 2019) for significantly upregulated (fold change ≥ 1.15; $p < 0.05$) and downregulated (fold change $\preceq 0.87$; $p < 0.05$) proteins.

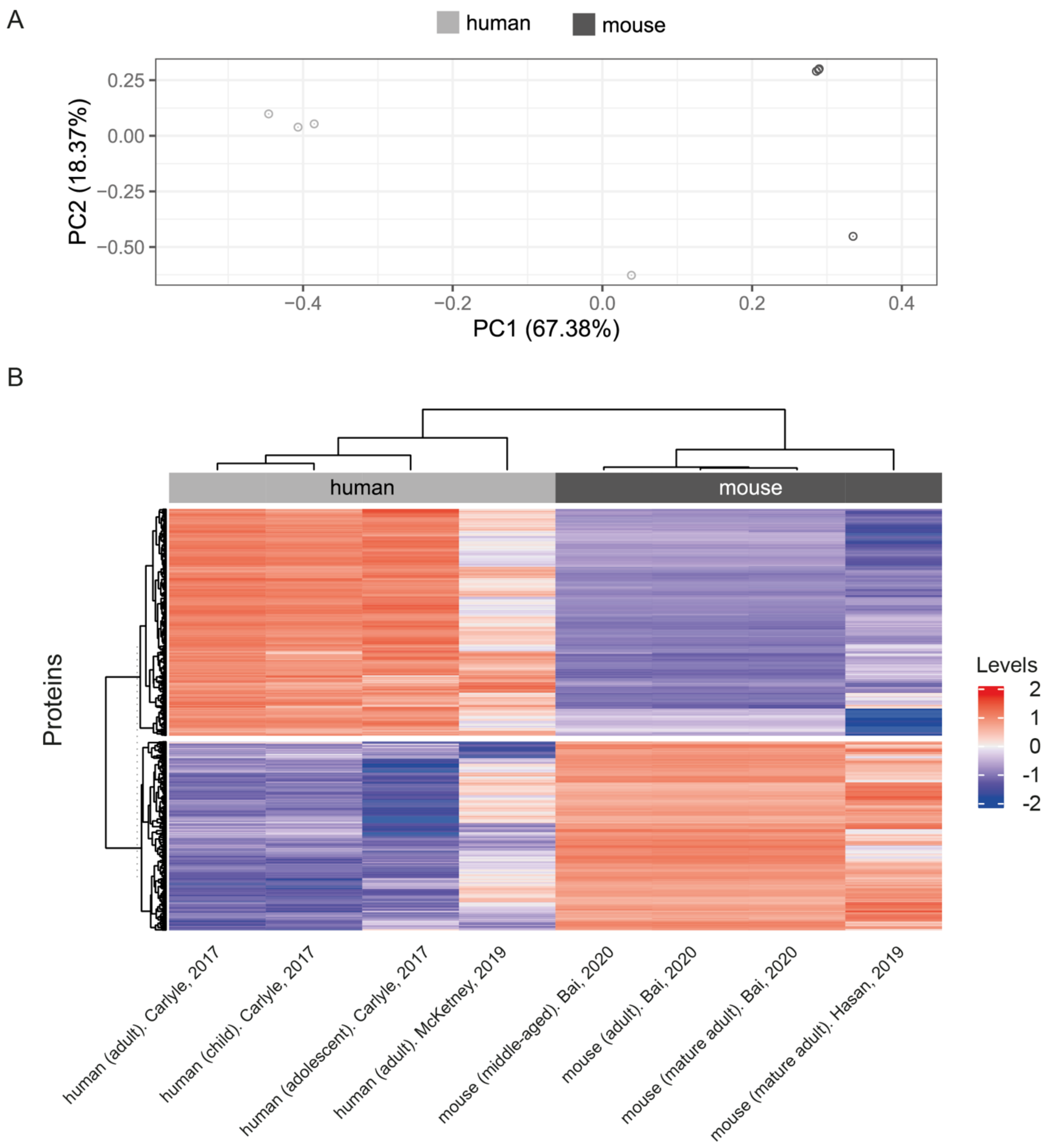

Supplementary Figure 7. Differential concentrations of proteins in the mouse and human brain.

(A) Principal component analysis performed on the subset of data from the Molecular Atlas protein concentrations. Percentages in the axes refer to the percentage of variance explained by each principal component.

(B) Hierarchical clustering of proteins with significantly different concentrations in mouse and human samples. Levels refer to row-scaled centered median-normalized Molecular Atlas concentrations.

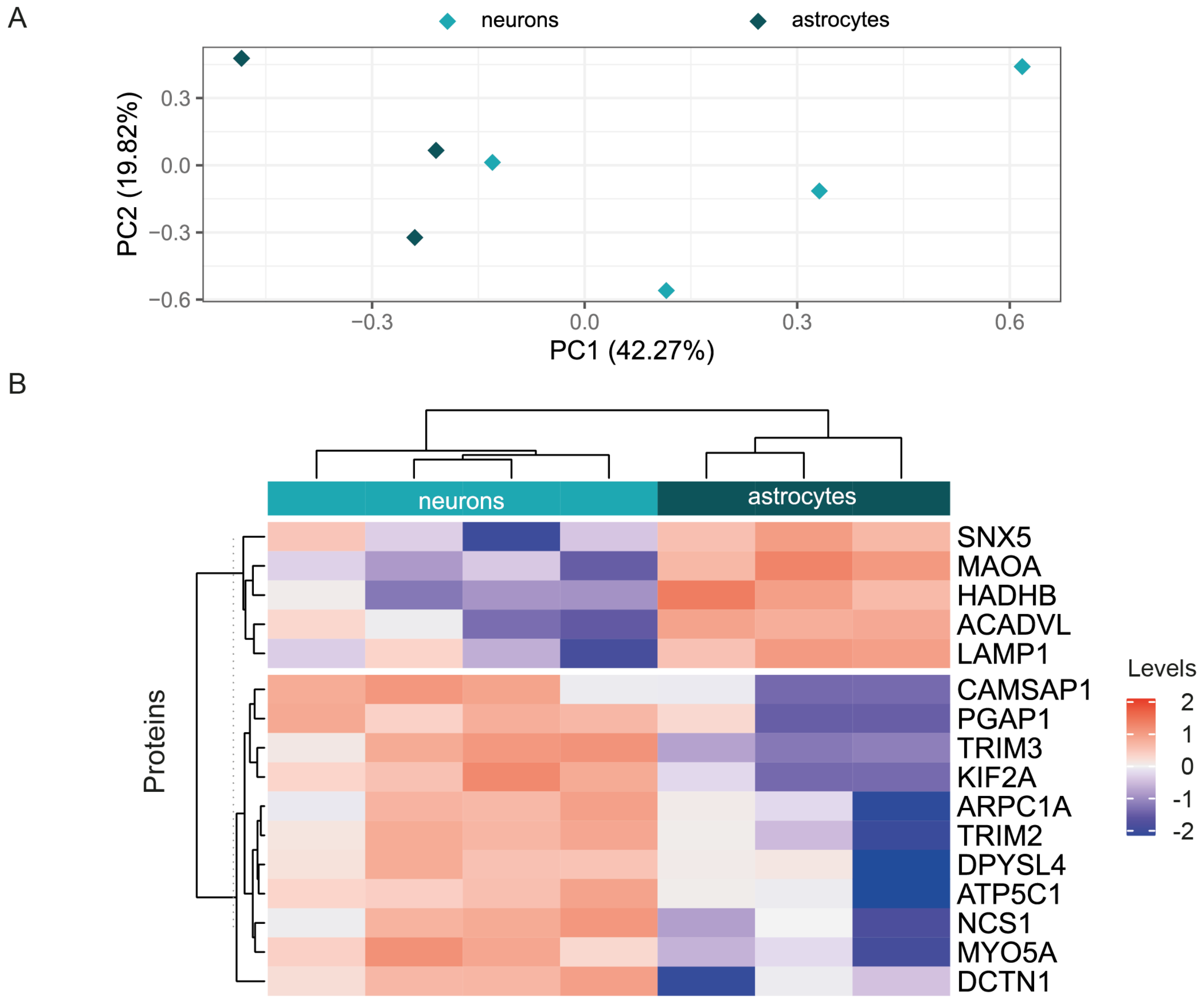

Supplementary Figure 8. Differential concentrations of proteins in neurons and astrocytes. (A) Principal component analysis performed on the subset of data from the Molecular Atlas protein concentrations. Percentages in the axes refer to the percentage of variance explained by each principal component. (B) Hierarchical clustering of proteins with significantly different concentrations in neurons and astrocytes. Levels refer to row-scaled centered median-normalized Molecular Atlas concentrations.

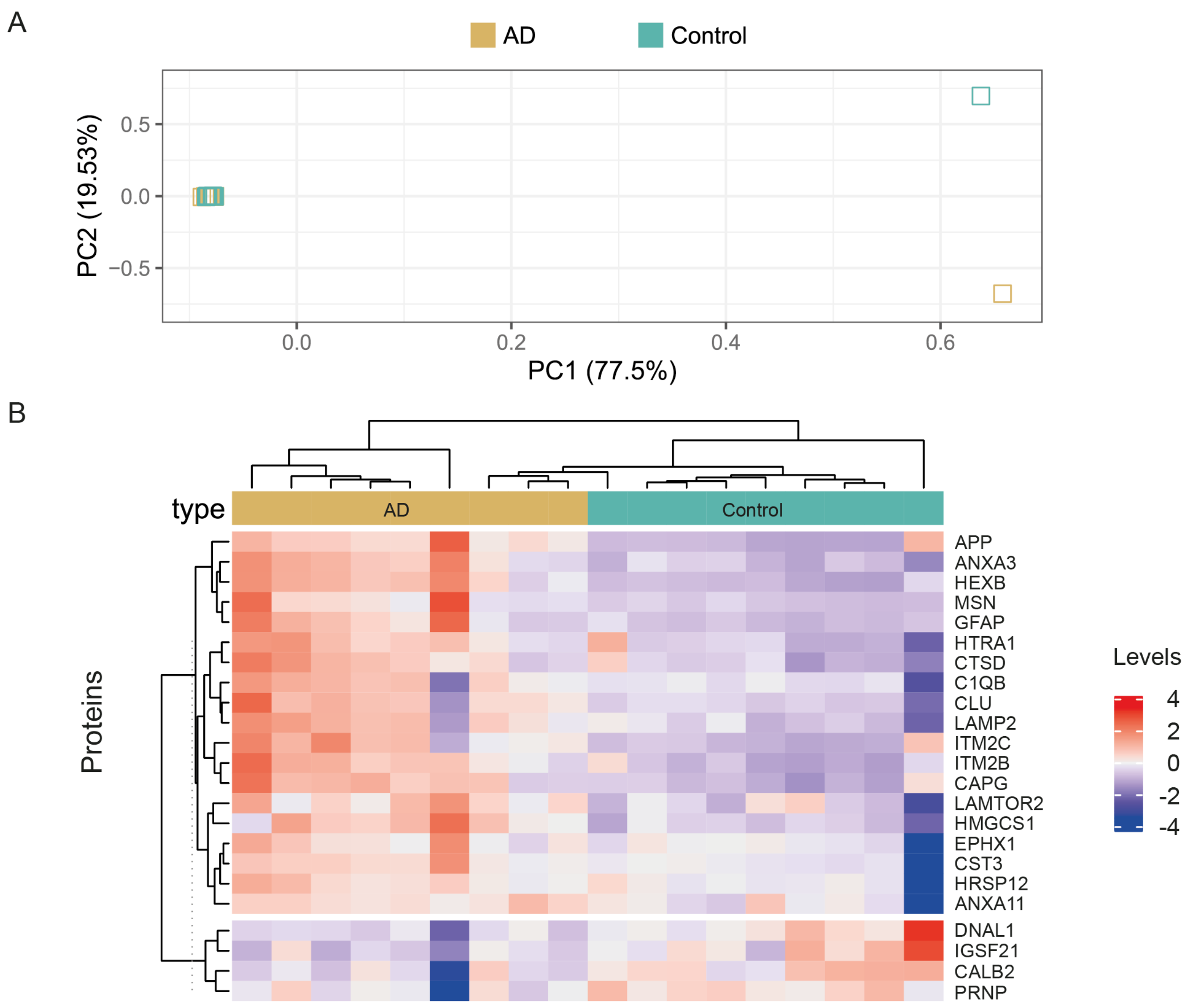

Supplementary Figure 9. Differential concentrations of proteins in the AD and healthy mouse cortex.

(A) Principal component analysis performed on the subset of data from the Molecular Atlas protein concentrations of healthy and AD mouse cortex samples. Percentages in the axes refer to the percentage of variance explained by each principal component.

(B) Hierarchical clustering of proteins with significantly different concentrations in healthy and AD mouse cortex samples. Levels refer to centered row-scaled concentrations.

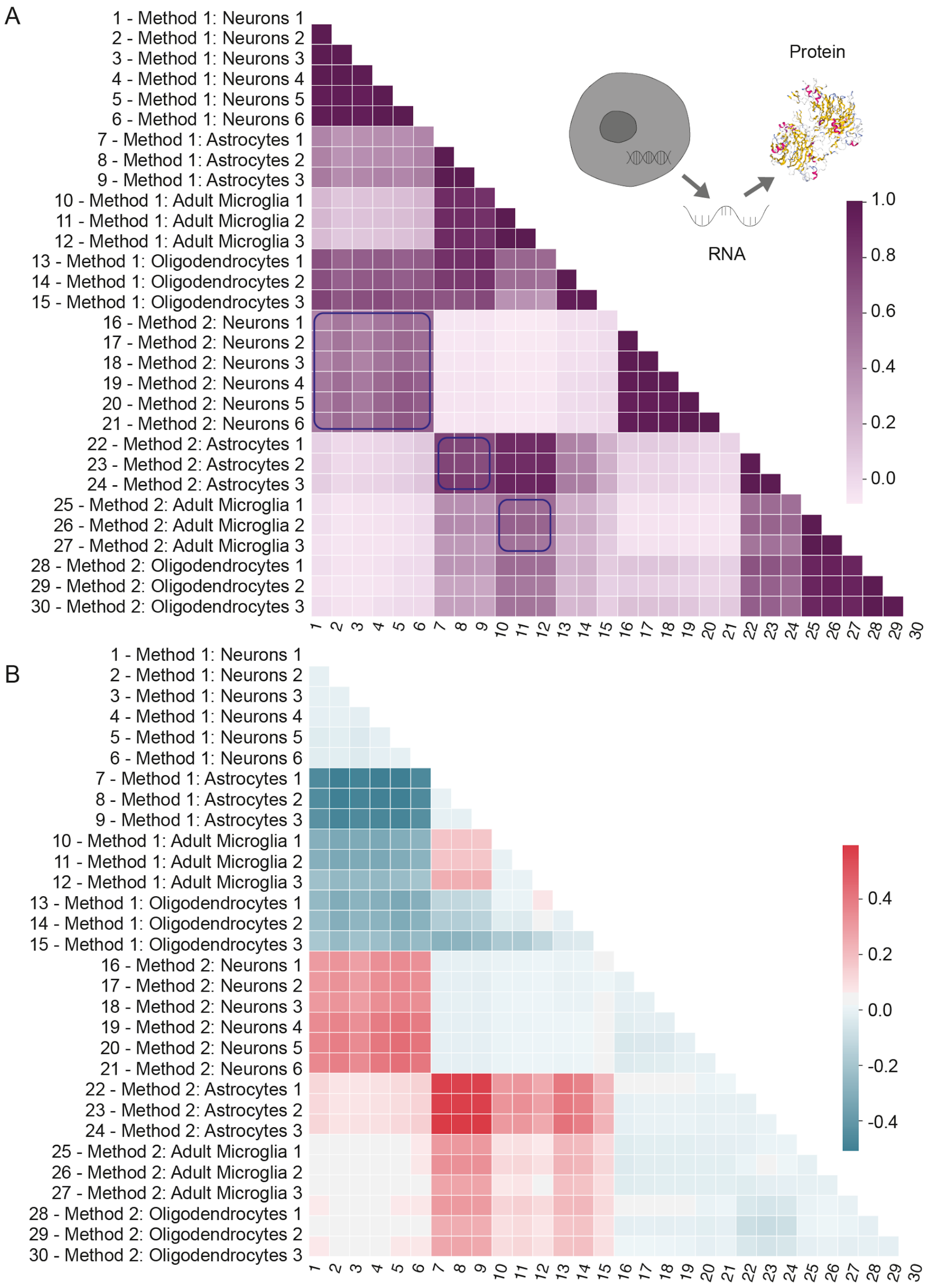

Supplementary Figure 10. Evaluation of the potential to use gene expression data for protein concentration estimates. (A) Comparison of protein concentrations independently calculated from transcriptomics RPKM data (specified as Method 1 in the figure) and proteomics LFQ data (Method

2). Pearson correlation coefficient is shown in color. The most important parts for the comparison of methods are outlined in purple. They show that protein concentrations estimated from transcriptomics and proteomics are well correlated in neurons, astrocytes and microglia.
(B) Difference between Pearson correlation coefficients calculated from concentrations and from "raw" mass spectrometry intensities and RPKM based on the subset of proteins from [30] and [5].

A

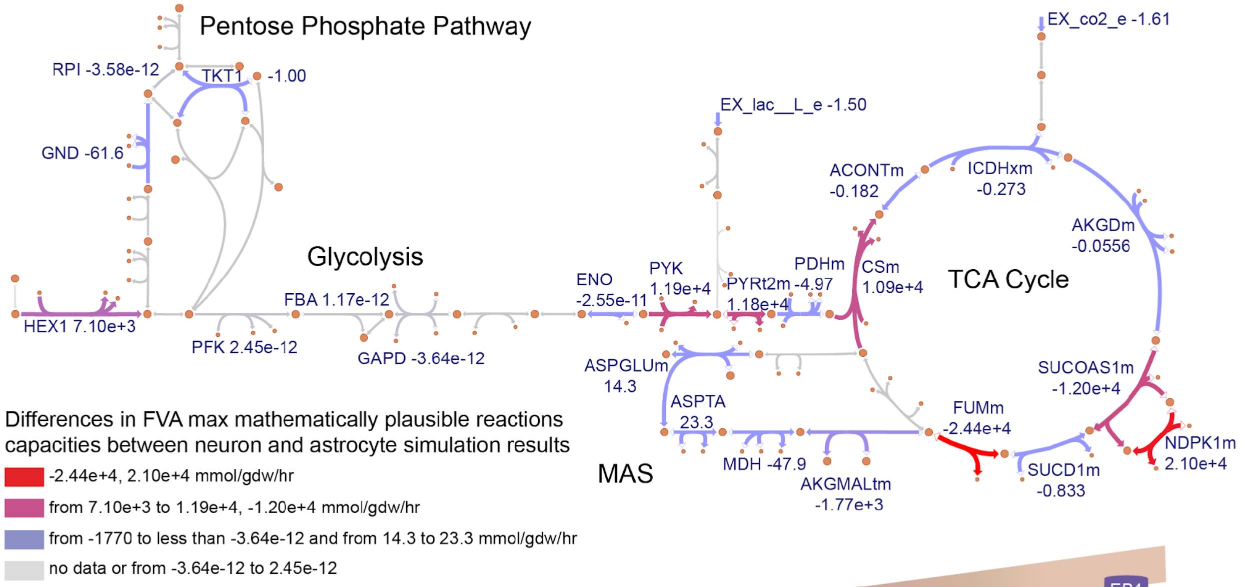

B

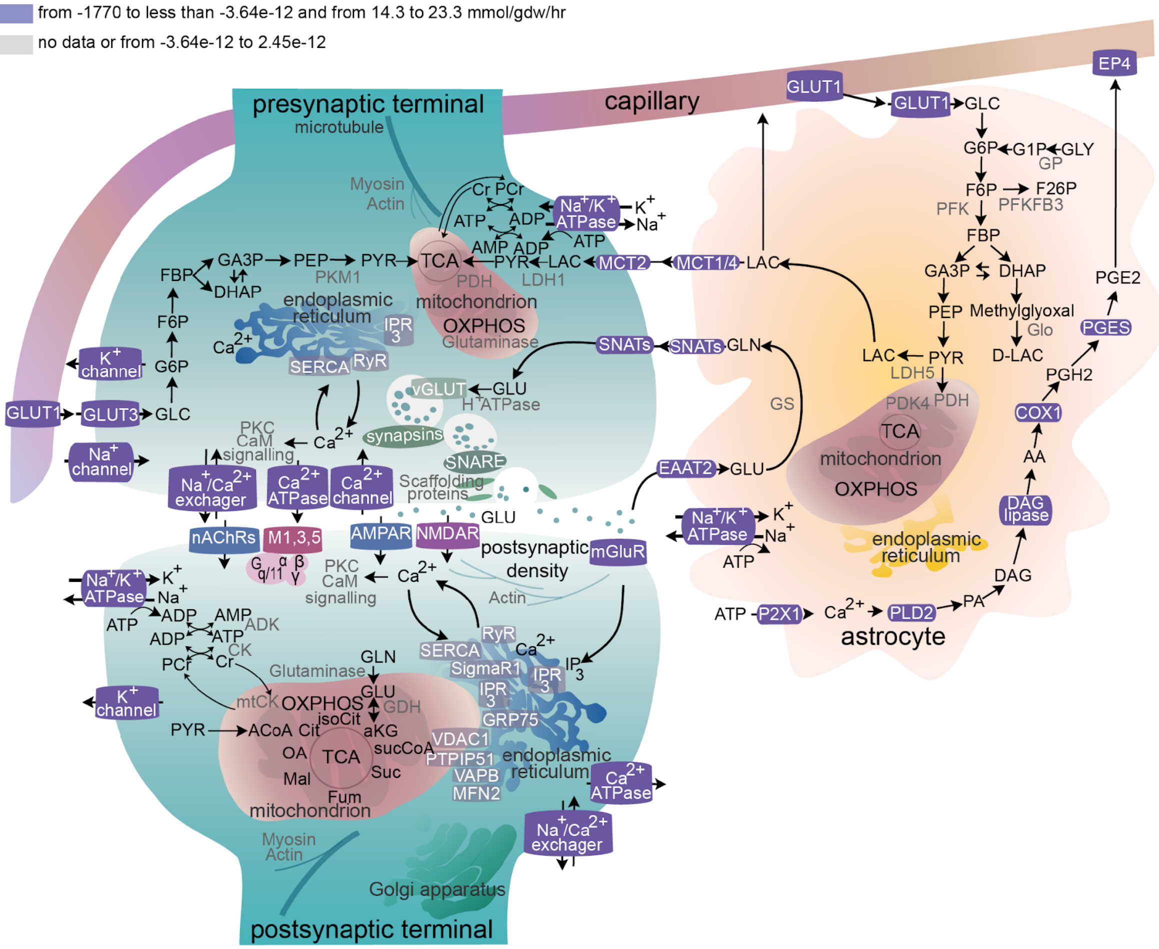

Supplementary Figure 11. Case study of brain metabolism. (A) Differences in neuronal and astrocytic maximal fluxes ($V_{max\ neuron}$ - $V_{max\ astrocyte}$), in units of mmol/gdw/hr. Only the main energy metabolism-related part of the model is shown. Abbreviations: FVA = flux variability analysis, MAS = malate-aspartate shuttle, TCA Cycle = tricarboxylic acid cycle, HEX = hexokinase, FBA = fructose-1,6-bisphosphate aldolase, PFK = phosphofructokinase, GAPD = glyceraldehyde 3-phosphate dehydrogenase, ENO = enolase, PYK = pyruvate kinase, PYRt2m = pyruvate mitochondrial transport via proton symport, PDHm = pyruvate dehydrogenase, EX_lac__L_e = L-lactate exchange (pseudoreaction), RPI = ribose-5-phosphate isomerase, TKT1 = transketolase, GND = phosphogluconate dehydrogenase, CSm = citrate synthase, ACONTm = aconitate hydratase, ICDHxm = isocitrate dehydrogenase NAD, EX_co2_e = CO2 exchange (pseudoreaction), AKGDm = 2-oxoglutarate dehydrogenase, SUCOAS1m = succinyl-CoA ligase (GDP-forming), NDPK1m = mitochondrial nucleoside-diphosphate kinase (ATP:GDP), SUCD1m = Succinate dehydrogenase, FUMm = mitochondrial fumarase, ASPGLUm = aspartate-glutamate mitochondrial shuttle, ASPTA = aspartate transaminase, MDH = malate dehydrogenase, AKGMALtm = mitochondrial dicarboxylate/tricarboxylate carrier (mal:akg). (B) Subset of subcellular processes related to energy metabolism in the NGV ensemble. Only some of the key relevant processes are shown for clarity. Abbreviations: GLC = glucose; GLY = glycogen; G6P = glucose-6P; F6P = fructose-6P; F26P = fructose-2,6P; FBP = fructose-1,6P; LAC = L-lactate; D-LAC = D-lactate; Glo = glyoxalase; PYR = pyruvate; PKM1/PKM2 = pyruvate kinase; PDH = pyruvate dehydrogenase; PDK4 = pyruvate dehydrogenase kinase 4; GLN = glutamine; GLU = glutamate; GDH = glutamate dehydrogenase; GS = glutamine synthetase; SNATs = sodium-coupled neutral amino acid transporters; M1,3,5 = muscarinic acetylcholine receptors types 1,3,5; nAChRs = nicotinic acetylcholine receptors; RyR = ryanodine receptor; SERCA = sarco-endoplasmic reticulum Ca^{2+}-ATPase; IP_3 = inositol triphosphate; IP_3R = IP_3 receptor; SigmaR1 = sigma non-opioid intracellular receptor 1; GRP75 = glucose - regulated protein 75 kDa; VDAC1 = voltage-dependent anion channel 1; PTPIP51 = protein tyrosine phosphatase-interacting protein 51; VAPB = vesicle-associated membrane protein-associated protein B; MFN2 = mitofusin 2; CaM = calmodulin; PKC = protein kinase C; mGluR = metabotropic glutamate receptor.

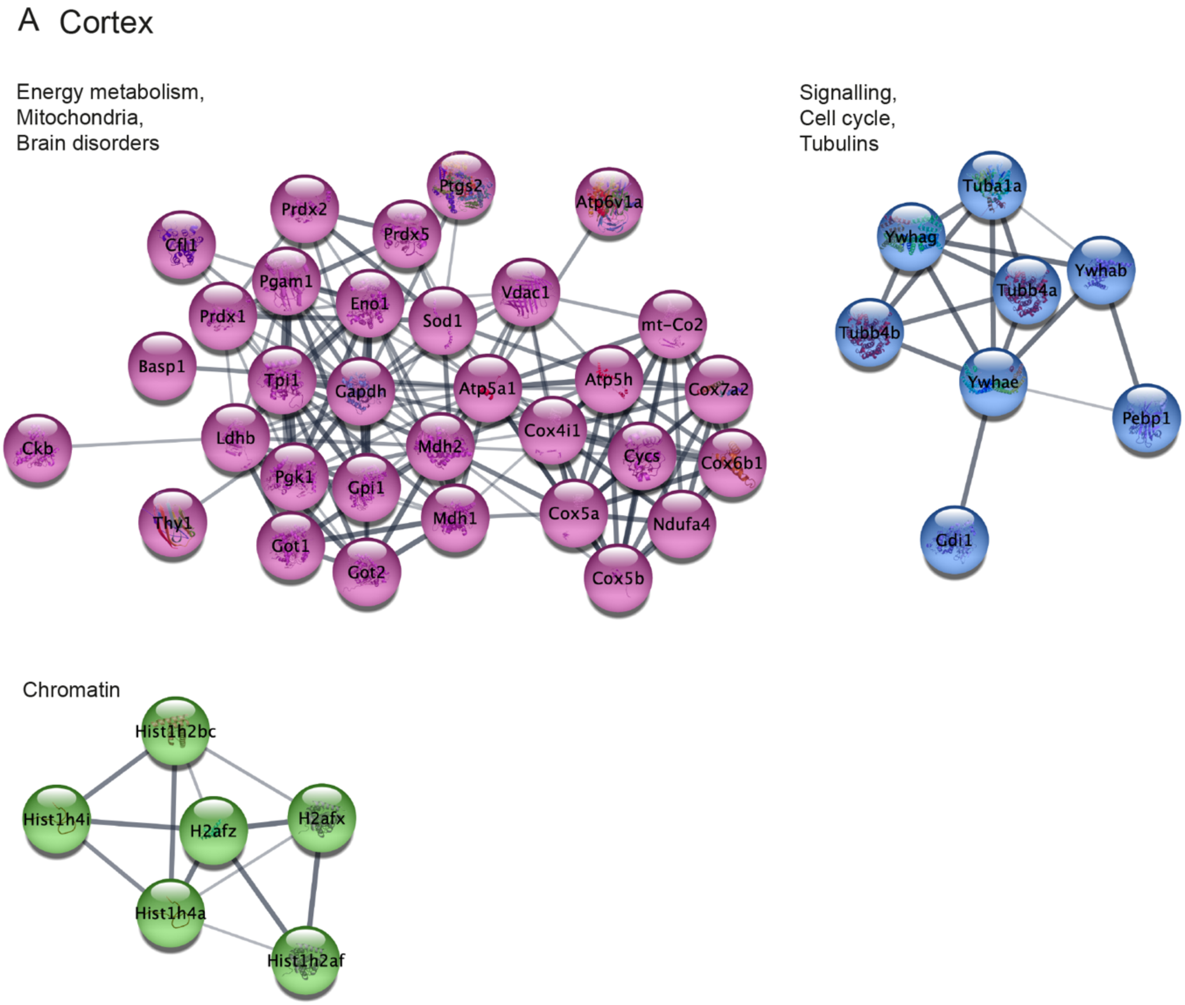

Supplementary Figure 11 (main text Figure 6A). The version of the main text Figure 6 with labels shown using bigger font.

B Hippocampus

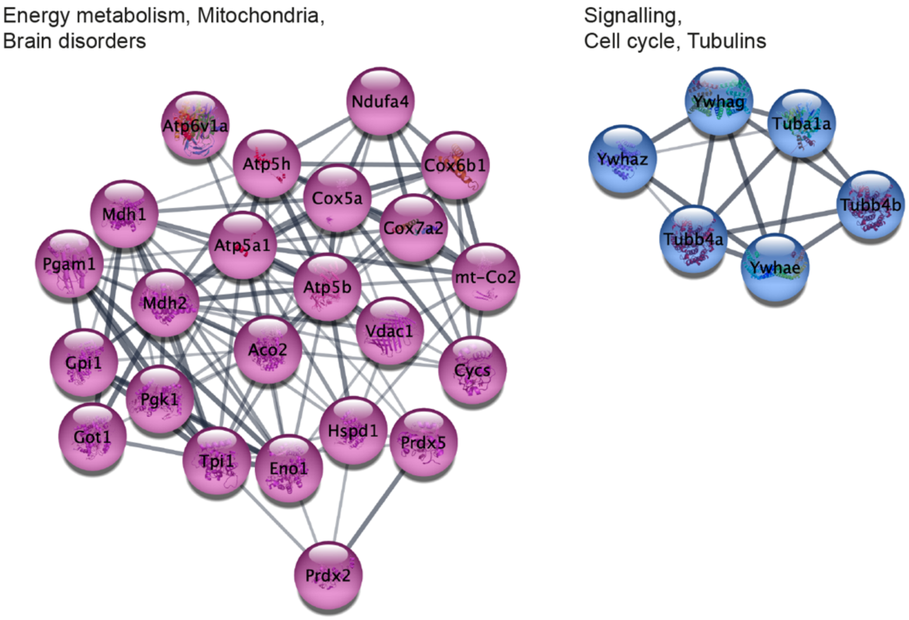

Supplementary Figure 12 (main text Figure 6B). The version of the main text Figure 6 with labels shown using bigger font.

C Striatum

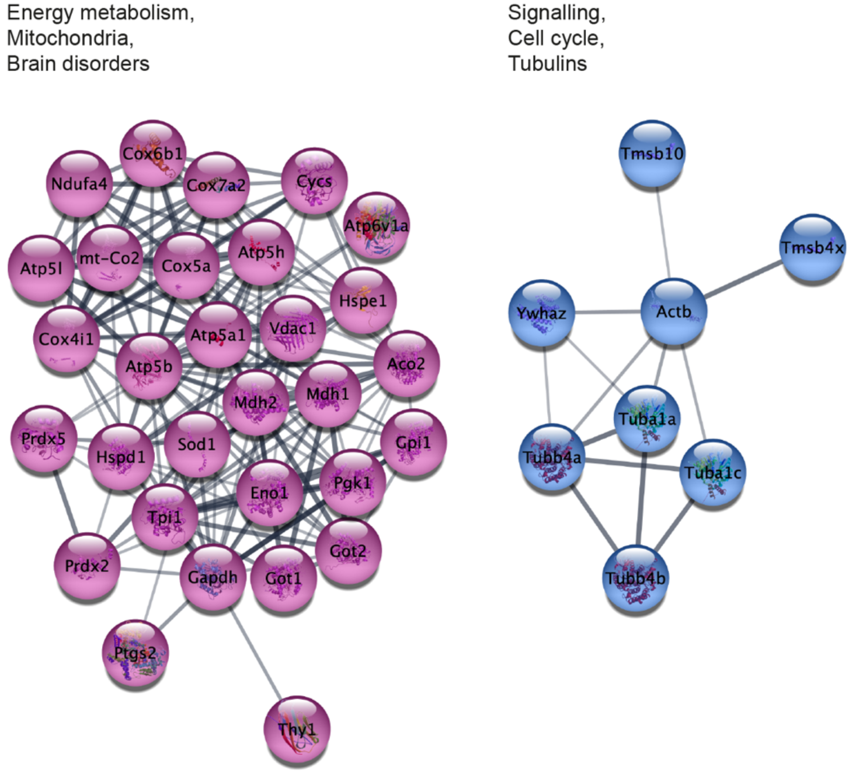

Supplementary Figure 13 (main text Figure 6C). The version of the main text Figure 6 with labels shown using bigger font.

D Thalamus

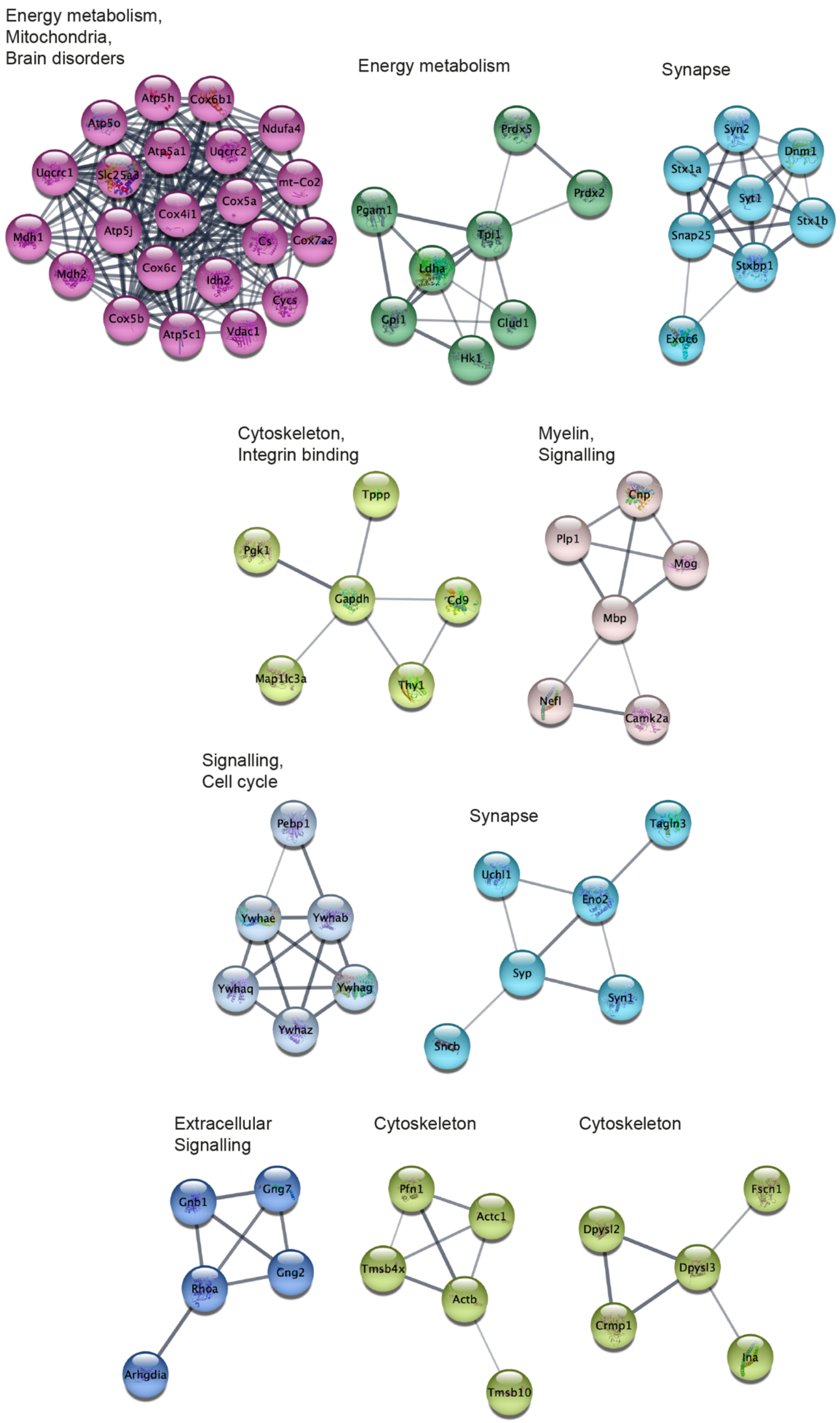
Energy metabolism,
Mitochondria,
Brain disorders
Atp5h
Cox6b1
Atp5o
Ndufa4
Atp5a1
Uqcrc2
Uqcrc1
Slc25a3
mt-Co2
Cox5a
Cox4i1
Atp5j
Cs
Cox7a2
Mdh1
Mdh2
Cox6c
Idh2
Cycs
Cox5b
Atp5c1
Vdac1
Energy metabolism
Prdx5
Prdx2
Pgam1
Tpi1
Ldha
Gpi1
Glud1
Hk1
Synapse
Syn2
Dnm1
Stx1a
Syt1
Stx1b
Snap25
Stxbp1
Exoc6
Cytoskeleton,
Integrin binding
Tppp
Pgk1
Gapdh
Cd9
Map1lc3a
Thy1
Myelin,
Signalling
Cnp
Plp1
Mog
Mbp
Nefl
Camk2a
Signalling,
Cell cycle
Pebp1
Ywhae
Ywhab
Ywhaq
Ywhag
Ywhaz
Synapse
Tagln3
Uchl1
Eno2
Syp
Syn1
Sncb
Extracellular
Signalling
Gng7
Gnb1
Gng2
Rhoa
Arhgdia
Cytoskeleton
Pfn1
Actc1
Tmsb4x
Actb
Tmsb10
Cytoskeleton
Fscn1
Dpysl2
Dpysl3
Crmp1
Ina

Supplementary Figure 14 (main text Figure 6D). The version of the main text Figure 6 with labels shown using bigger font.

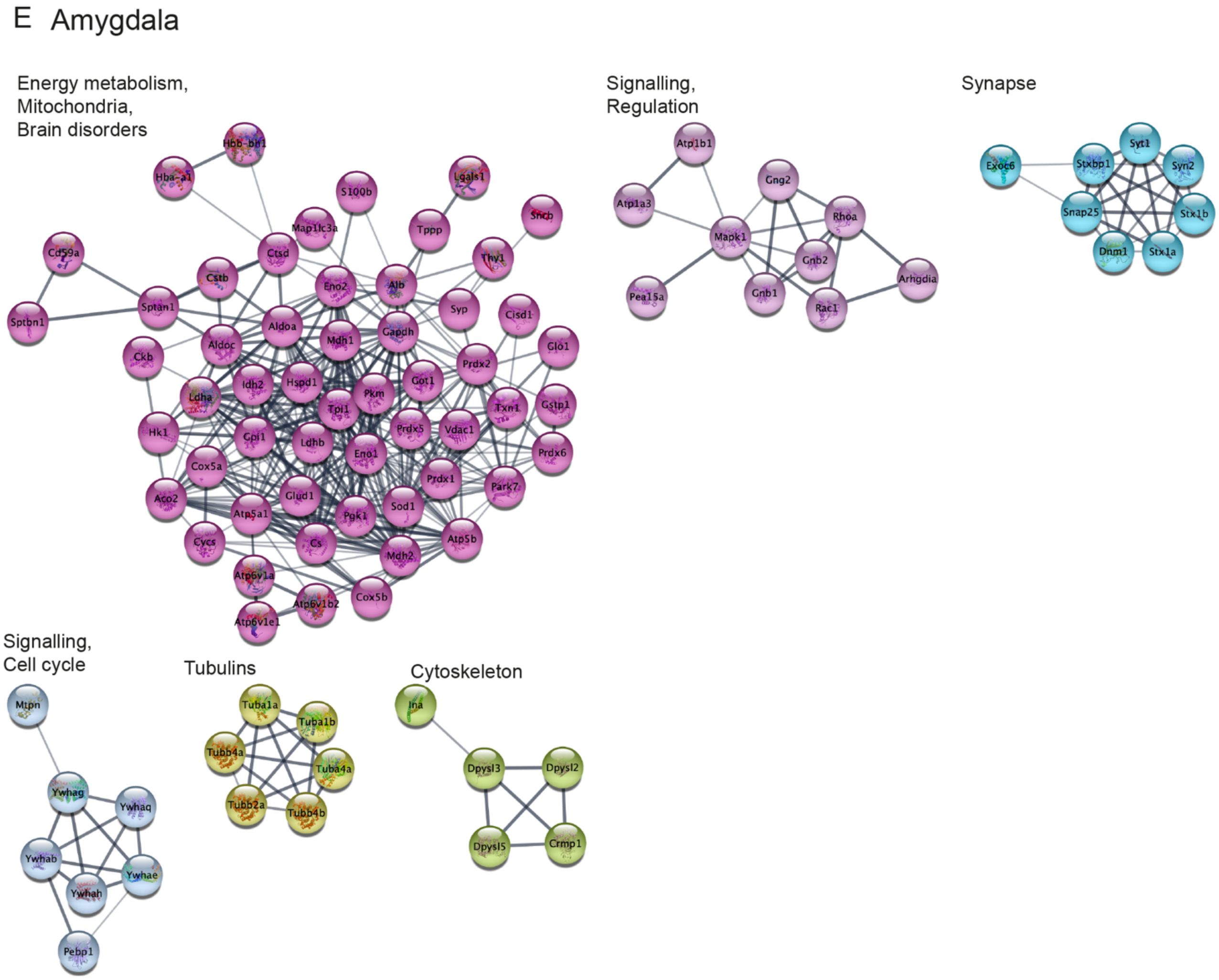

Supplementary Figure 15 (main text Figure 6E). The version of the main text Figure 6 with labels shown using bigger font.

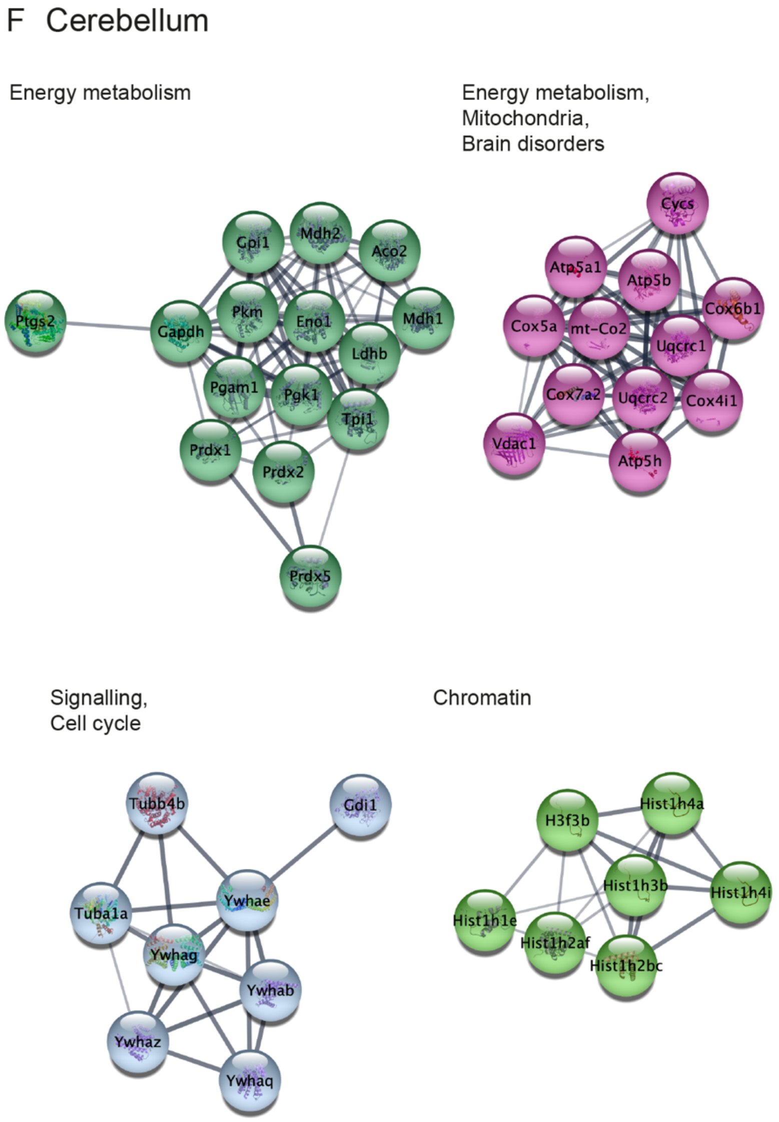

Supplementary Figure 16 (main text Figure 6F). The version of the main text Figure 6 with labels shown using bigger font.

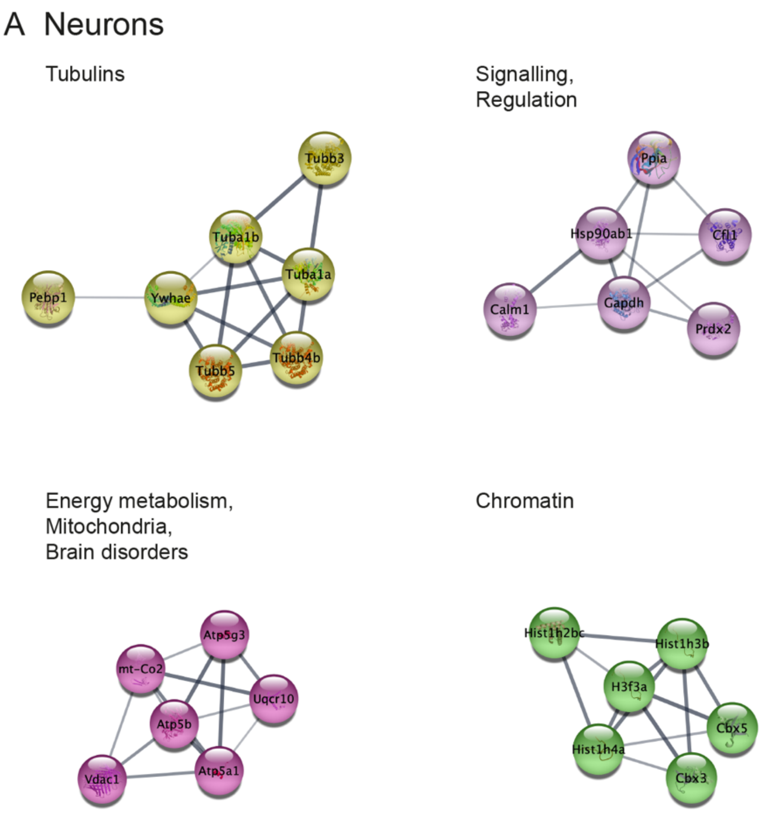

Supplementary Figure 17 (main text Figure 7A). The version of the main text Figure 7 with labels shown using bigger font.

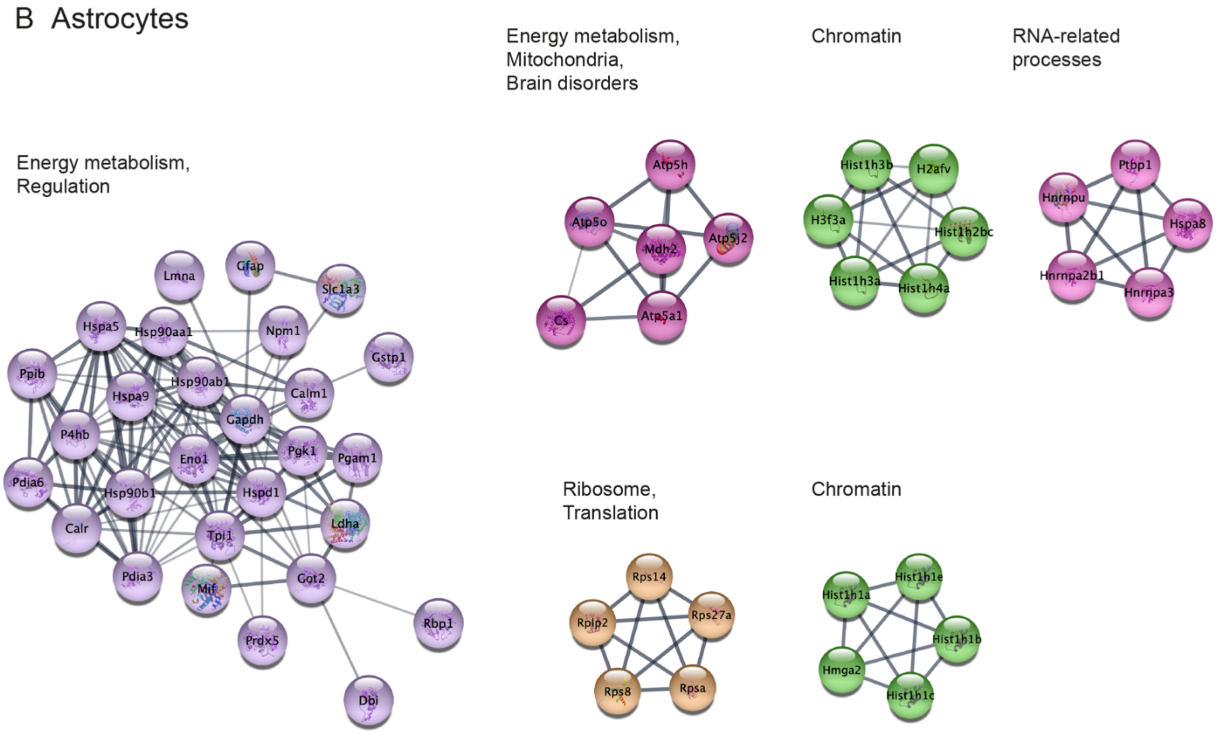

Supplementary Figure 18 (main text Figure 7B). The version of the main text Figure 7 with labels shown using bigger font.

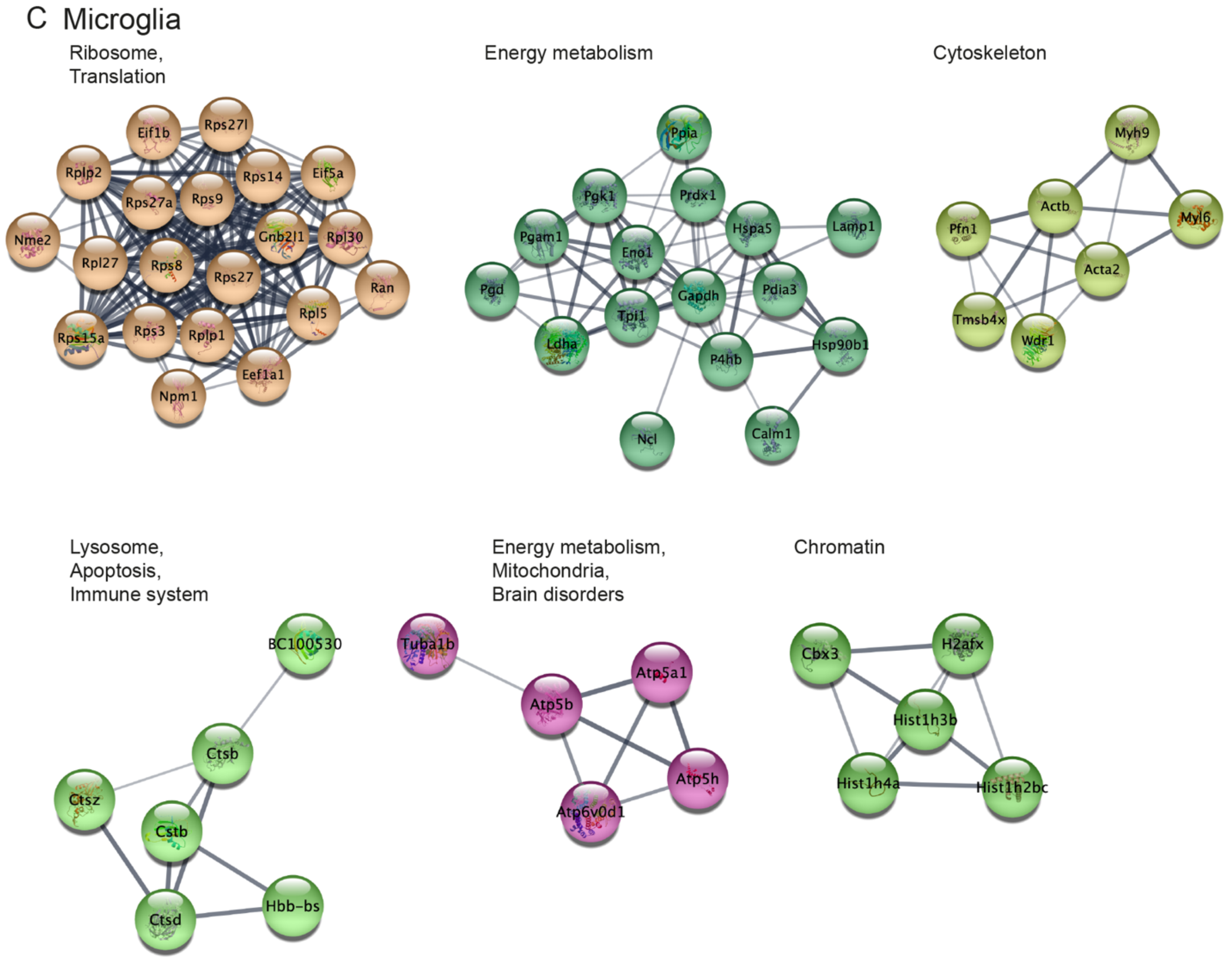

Supplementary Figure 19 (main text Figure 7C). The version of the main text Figure 7 with labels shown using bigger font.

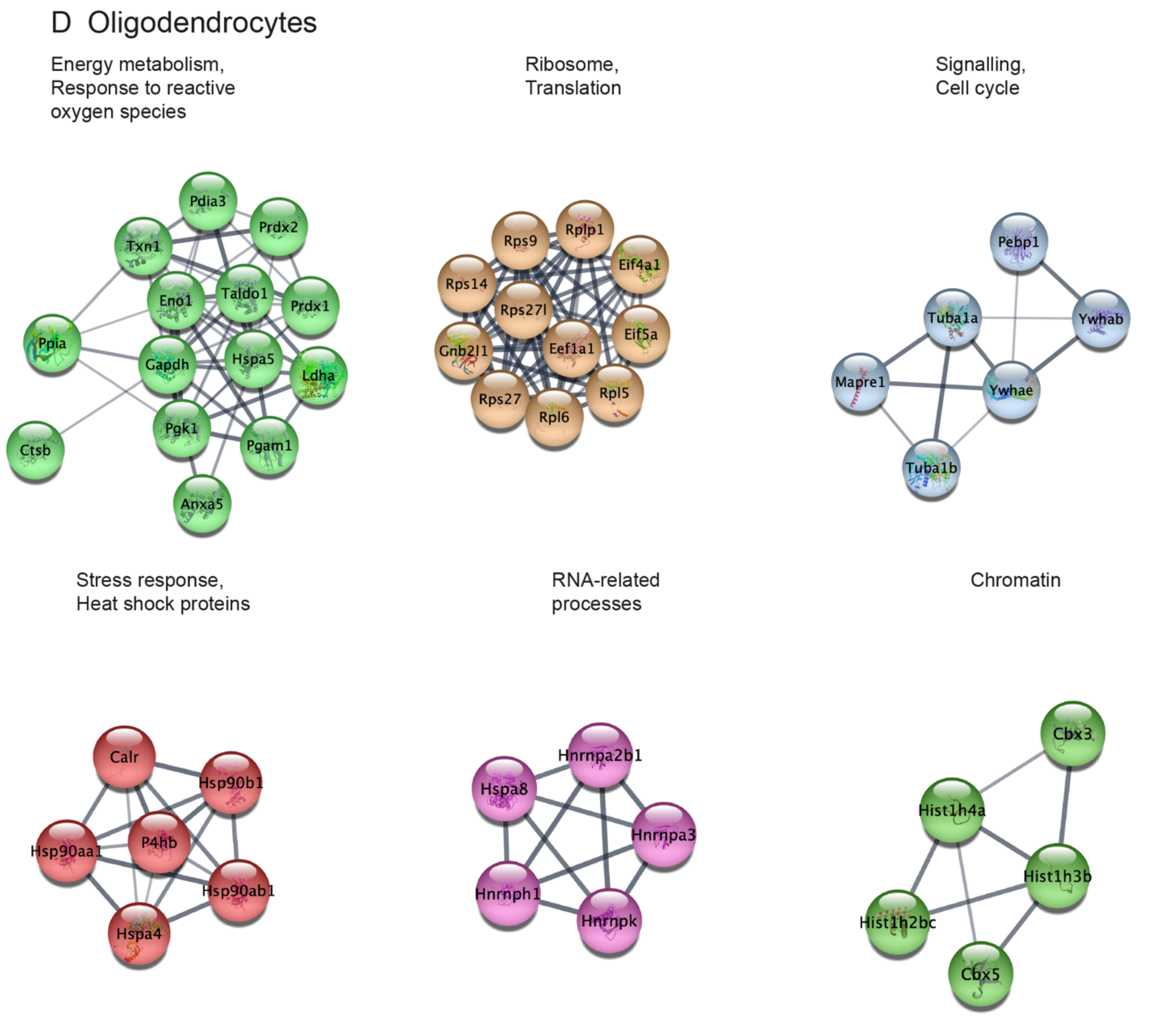

Supplementary Figure 20 (main text Figure 7D). The version of the main text Figure 7 with labels shown using bigger font.

3 The Molecular Atlas application in constraint-based modeling (flux variability analysis)

Methods

Flux balance and variability analyses are some of the most common types of constraint-based modelling, that allow the prediction of the flow of metabolites in a network of reactions, and subsequently, the metabolic capacities of biochemical networks. One of the substantial limitations for interpretability of the results in these kinds of studies is the high uncertainty in the properties of the investigated system, such as a range of mathematically plausible reaction fluxes. To this end, many alternatives have been proposed to better constrain the models (Gavai et al. 2015; Heckmann et al. 2018; Lularevic et al. 2019; Pandey, Hadadi, and Hatzimanikatis 2019; Sánchez et al. 2017; Tian and Reed 2018), with one of the widely-proposed means being gene expression data and enzyme

turnover. One possible way is the incorporation of protein expression and allocation that leads to producing so-called Metabolism and Expression (ME) models (O'Brien and Palsson 2015) that are available for simple organisms, but great efforts are needed to apply this approach for accurate simulations of mammalian metabolism. On the other hand, the limited capacity of enzymes constrains the available range of reaction fluxes (Noor et al. 2016). The capacity of enzymes, which define maximal reaction rates, is reflected in their activities and amounts. The latter often has been represented by the expression of corresponding genes. At the same time, using protein concentrations instead of gene expression can lead to more biologically accurate results due to gene-protein level differences defined by regulatory processes of protein synthesis and degradation. While there are many variations of the flux balance and flux variability methods, the key idea is to analyse the flow of metabolites through the metabolic network optimizing a specified objective function given some mathematical constraints on the reaction rates (Orth, Thiele, and Palsson 2010; Anand, Mukherjee, and Padmanabhan 2020). FBA has a wide range of applications in bioengineering and biotechnology, but its use for brain cells remains limited. Nonetheless some studies are available, including but not limited to those addressing neuron-astrocyte coupling (Cakir et al. 2007; Lewis et al. 2010; DiNuzzo et al. 2017), comparisons of health and diseased states (Cakir et al. 2007; Supandi and van Beek 2018), and a genome-scale astrocytic metabolic network (Martín-Jiménez et al. 2017).

The main factors that affect rates and fluxes of biochemical reactions are catalytic activities, concentrations of enzymes, availability of reactants and co-factors, and the physical state of the system, such as temperature. Under the assumptions that the same enzymes have similar activities in neurons and astrocytes, that conditions and presence of reactants and co-factors are also comparable, the enzyme concentrations should explain differences in the reaction fluxes when comparing neurons and astrocytes. So we aimed to evaluate whether protein concentrations used as constraints will result in meaningful relative maximum capacities of reactions in the neuron and astrocyte. Comparison of the results to what can be achieved with the other types of constraints for the fluxes is out of the scope of our study. We used our protein concentrations data to constrain large scale models of neuron and astrocyte metabolism, and to perform flux balance analysis (FBA) and loopless flux variability analysis (FVA) (Desouki et al. 2015; Schellenberger, Lewis, and Palsson 2011) maximizing an objective function of total adenosine triphosphate (ATP) production. FVA is used to evaluate reaction fluxes boundaries, within which multiple equally optimal solutions can be contained. Loopless FVA is a modification of this method aiming to eliminate thermodynamically infeasible loops (Schellenberger, Lewis, and Palsson 2011). We used the mouse metabolism model iMM1415 (Sigurdsson et al. 2010) as a core reconstruction, but we updated an objective function to represent ATP production as given by the equation (5) below.

$$ATP_{production} = AP4AH1 - AP4AH1_{rev} + ATPS4m - ATPS4m_{rev} + ATPtm - ATPtm_{rev} - NDPK1m + NDPK1m_{rev} + PYK - PYK_{rev} + SUCD1m - SUCD1m_{rev} - SUCOAS1m + SUCOAS1m_{rev} \quad (5)$$

Abbreviations: AP4AH1 = P1,P4-bis(5'-adenosyl)-tetraphosphate adenylohydrolase, ATPS4m = ATP synthase, ATPtm = mitochondrial ADP/ATP transporter, NDPK1m = mitochondrial nucleoside-diphosphate kinase (ATP:GDP), PYK = pyruvate kinase, SUCD1m = succinate dehydrogenase, SUCOAS1m = succinyl-CoA ligase (GDP-forming). While some reactions are clearly irreversible, both forward and reverse (specified by subscript "rev" in equation (5)) directions are given in the computational framework and reversibility is accounted for in flux boundaries. This objective function includes some key ATP and guanosine triphosphate (GTP) producing reactions and succinate dehydrogenase, which is an important link between the TCA cycle and the electron transport chain.

Using gene-protein-reaction rules (GPR) from the original model, which are basically the relations between proteins and biochemical reactions, we set constraints on the lower and upper bounds of fluxes based on protein concentration data from the Brain Molecular Atlas for neurons, astrocytes, cortex and hippocampus, producing four models respectively. In the case of complex gene-protein-reaction relationships containing AND and OR expressions, constraints were set based on logic of subunits or isoforms of enzymes being rate limiting for the reaction. Natural logarithm protein concentrations were scaled to the range of zero to one and then multiplied by standard bounds of -100,000 and 100,000 mmol/gdw/hr for lower and upper bounds, respectively. Reversibility of reactions was considered by setting relevant bounds to zero.

For loopless FVA, we set the fraction of optimum parameter to 0.9 to reduce the number of biologically implausible solutions. We used GUROBI solver (Gurobi Optimization, LLC 2019) in simulations with CobraPy (Ebrahim et al. 2013). Escher (King et al. 2015) Python library and "RECON1.Glycolysis TCA PPP" map were used for visualization of fluxes.

Results

As all this information is available through the Brain Molecular Atlas, we applied it to perform loopless flux variability analysis (FVA) (Desouki et al. 2015; Schellenberger, Lewis, and Palsson 2011) with the objective function set to be ATP production as described in Methods section. Only healthy-state data was used for this analysis. We compared individual maximal reaction capacities (V_{max}) for the main energy metabolism pathways of neuron and astrocyte based on results from loopless FVA (Supplementary Figure 11A).

Qualitatively, we can see expected differences between neuron and astrocyte energy metabolism from our simulations, consistent with reported experimental results as shown in the following observations. First, there is evidence that hexokinase is highly enriched in neurons compared to astrocytes, in mouse as well as in human cortex (Lundgaard et al., 2015). Differences in the maximal capacity of tricarboxylic acid cycle enzymes (citrate synthase), pyruvate kinase flux and pyruvate transport to mitochondria between neurons and astrocytes can be explained by differential preferences of oxidative to glycolytic energy metabolism in these cell types. Moreover, it is known that the malate-aspartate shuttle (MAS) is active in neurons but much less so in astrocytes (McKenna et al. 2006). Particularly, aspartate–glutamate carrier aralar1 is highly enriched on neurons compared to astrocytes (Bélanger et al., 2011). Our simulation qualitatively supports this, as the difference between neuronal and astrocytic "aspartate-glutamate mitochondrial shuttle" (ASPGLUm) flux capacities is positive. Citrate synthase (CSm) has a higher maximal capacity in the neuron than in the astrocyte in the simulation. This is consistent with the information about higher activity of citrate synthase in neurons than in astrocytes (Hassel and Brâthe, 2000). Next, a large difference in maximum capacities is observed for nucleoside diphosphate kinase (NDPK), which is known to be important for presynaptic function (Krishnan et al., 2001), to play a role in neural development, as well as to be associated with a variety of brain disorders (Ansoleaga et al., 2016; Hwan Kim et al., 2002; Qiu et al., 2018), and is reported to be expressed mainly in neurons (Garcia-Esparcia et al., 2015). NDPKs, in general, are multifunctional proteins (Attwood and Muimo, 2018).

The biological context of compared processes is shown below in Supplementary Figure 11B for better understanding of their relations to other pathways, including those involved in neuronal signal transduction and the astrocytic glutamate-glutamine cycle.

References are available from the main text bibliography.

4 Supplementary Information on Sample Sizes

Figure 1. Schematic figure, no sample sizes.

Figure 2. An example of data before and after normalization for the experimental methods used to obtain the data.
(A) 590 entries from 24 data sets (same as in (A)).
Horizontal line corresponds to the median value and is drawn at the level of 20.3 a.u.
Sample sizes for per data set:
Bai 2020: 26
Beltran 2016: 1
Carlyle 2017: 77
Chuang 2018: 4
Davis 2019: 21
Fecher 2019: 34
Fornasiero 2018: 1
Geiger 2013: 10
Guergues 2019: 3
Hamezah 2018: 12
Hamezah 2019: 6
Han 2014: 9
Hasan 2020: 72
Hosp 2017, CSF: 8
Hosp 2017, insoluble: 37
Hosp 2017, soluble: 80
Itzhak 2017: 1
Kjell 2020: 28
Krogager 2018: 2
McKetney 2019: 70
Sharma 2015, cultured: 35
Sharma 2015, isolated: 35
Wisniewski 2015: 6
Zhu 2018: 12

(B) 590 entries from 24 data sets (same as in (A)).
Horizontal line corresponds to the median value and is drawn at the level of 1.57 uM.
Sample sizes for per data set (same as in (A)):
Bai 2020: 26
Beltran 2016: 1
Carlyle 2017: 77
Chuang 2018: 4
Davis 2019: 21
Fecher 2019: 34
Fornasiero 2018: 1

Geiger 2013: 10
Guergues 2019: 3
Hamezah 2018: 12
Hamezah 2019: 6
Han 2014: 9
Hasan 2020: 72
Hosp 2017, CSF: 8
Hosp 2017, insoluble: 37
Hosp 2017, soluble: 80
Itzhak 2017: 1
Kjell 2020: 28
Krogager 2018: 2
McKetney 2019: 70
Sharma 2015, cultured: 35
Sharma 2015, isolated: 35
Wisniewski 2015: 6
Zhu 2018: 12

(C) Protein levels in different data sets before and after normalization. Sample sizes for violin plots per data set:
Bai 2020: 242008
Beltran 2016: 2389
Carlyle 2017: 363748
Chuang 2018: 10552
Davis 2019: 32696
Fecher 2019: 128733
Fornasiero 2018: 1651
Geiger 2013: 24722
Guergues 2019: 14013
Hamezah 2018: 8720
Hamezah 2019: 11564
Han 2014: 56202
Hasan 2020: 260202
Hosp 2017, CSF: 4916
Hosp 2017, insoluble: 32466
Hosp 2017, soluble: 293790
Itzhak 2017: 9019
Kjell 2020: 94128
Krogager 2018: 4414
McKetney 2019: 191456
Sharma 2015, cultured: 167579
Sharma 2015, isolated: 142430
Wisniewski 2015: 19508
Zhu 2018: 13200

Figure 3. The effects of the data processing pipeline on protein levels.

(A) Protein levels before normalization of 74 proteins that are present in the largest number of collected data sets (24 of 25 data sets).
Sample sizes:
LFQ,TMT,SILAC: 23713
iBAQ: 7177
mol/g protein: 244
uM: 79
Sample sizes per protein per group:
ACAT1, LFQ,TMT,SILAC: 330
ACAT1, uM: 1
ACAT1, iBAQ: 85
ACAT1, mol/g protein: 3
ACO2, LFQ,TMT,SILAC: 309
ACO2, uM: 1
ACO2, iBAQ: 92
ACO2, mol/g protein: 3
ADD1, LFQ,TMT,SILAC: 398
ADD1, uM: 2
ADD1, iBAQ: 152
ADD1, mol/g protein: 6
ADD3, LFQ,TMT,SILAC: 335
ADD3, uM: 1
ADD3, iBAQ: 92
ADD3, mol/g protein: 3
ALDOA, LFQ,TMT,SILAC: 337
ALDOA, uM: 1
ALDOA, iBAQ: 130
ALDOA, mol/g protein: 3
ALDOC, LFQ,TMT,SILAC: 310
ALDOC, uM: 1
ALDOC, iBAQ: 83
ALDOC, mol/g protein: 3
ANXA6, LFQ,TMT,SILAC: 365
ANXA6, uM: 1
ANXA6, iBAQ: 84
ANXA6, mol/g protein: 6
ATP5B, LFQ,TMT,SILAC: 336
ATP5B, uM: 1
ATP5B, iBAQ: 104
ATP5B, mol/g protein: 3
BASP1, LFQ,TMT,SILAC: 312
BASP1, uM: 1
BASP1, iBAQ: 96
BASP1, mol/g protein: 5
CAND1, LFQ,TMT,SILAC: 311
CAND1, uM: 1
CAND1, iBAQ: 101

CAND1, mol/g protein: 3
CAP1, LFQ,TMT,SILAC: 310
CAP1, uM: 1
CAP1, iBAQ: 85
CAP1, mol/g protein: 3
CAPZB, LFQ,TMT,SILAC: 422
CAPZB, uM: 1
CAPZB, iBAQ: 131
CAPZB, mol/g protein: 6
CCT2, LFQ,TMT,SILAC: 305
CCT2, uM: 1
CCT2, iBAQ: 82
CCT2, mol/g protein: 3
CCT3, LFQ,TMT,SILAC: 315
CCT3, uM: 1
CCT3, iBAQ: 96
CCT3, mol/g protein: 3
CCT4, LFQ,TMT,SILAC: 305
CCT4, uM: 1
CCT4, iBAQ: 86
CCT4, mol/g protein: 3
CKB, LFQ,TMT,SILAC: 328
CKB, uM: 1
CKB, iBAQ: 95
CKB, mol/g protein: 3
CYB5R3, LFQ,TMT,SILAC: 305
CYB5R3, uM: 1
CYB5R3, iBAQ: 82
CYB5R3, mol/g protein: 3
DPYSL2, LFQ,TMT,SILAC: 309
DPYSL2, uM: 1
DPYSL2, iBAQ: 108
DPYSL2, mol/g protein: 3
EEF1A1, LFQ,TMT,SILAC: 315
EEF1A1, uM: 1
EEF1A1, iBAQ: 103
EEF1A1, mol/g protein: 3
EEF1G, LFQ,TMT,SILAC: 305
EEF1G, uM: 1
EEF1G, iBAQ: 94
EEF1G, mol/g protein: 3
EEF2, LFQ,TMT,SILAC: 309
EEF2, uM: 1
EEF2, iBAQ: 86
EEF2, mol/g protein: 3
EZR, LFQ,TMT,SILAC: 299
EZR, uM: 1
EZR, iBAQ: 89

EZR, mol/g protein: 3
FASN, LFQ,TMT,SILAC: 309
FASN, uM: 1
FASN, iBAQ: 87
FASN, mol/g protein: 3
GAPDH, LFQ,TMT,SILAC: 344
GAPDH, uM: 1
GAPDH, iBAQ: 149
GAPDH, mol/g protein: 3
GDI2, LFQ,TMT,SILAC: 305
GDI2, uM: 1
GDI2, iBAQ: 93
GDI2, mol/g protein: 3
GLUD1, LFQ,TMT,SILAC: 326
GLUD1, uM: 1
GLUD1, iBAQ: 96
GLUD1, mol/g protein: 3
GNB1, LFQ,TMT,SILAC: 309
GNB1, uM: 1
GNB1, iBAQ: 91
GNB1, mol/g protein: 3
GNB2, LFQ,TMT,SILAC: 310
GNB2, uM: 1
GNB2, iBAQ: 74
GNB2, mol/g protein: 3
HK1, LFQ,TMT,SILAC: 315
HK1, uM: 1
HK1, iBAQ: 98
HK1, mol/g protein: 3
HNRNPA2B1, LFQ,TMT,SILAC: 318
HNRNPA2B1, uM: 2
HNRNPA2B1, iBAQ: 93
HNRNPA2B1, mol/g protein: 3
HNRNPK, LFQ,TMT,SILAC: 379
HNRNPK, uM: 2
HNRNPK, iBAQ: 128
HNRNPK, mol/g protein: 3
HSP90AA1, LFQ,TMT,SILAC: 321
HSP90AA1, uM: 1
HSP90AA1, iBAQ: 110
HSP90AA1, mol/g protein: 3
HSP90AB1, LFQ,TMT,SILAC: 322
HSP90AB1, uM: 1
HSP90AB1, iBAQ: 103
HSP90AB1, mol/g protein: 3
HSP90B1, LFQ,TMT,SILAC: 309
HSP90B1, uM: 1

HSP90B1, iBAQ: 86
HSP90B1, mol/g protein: 3
HSPA4, LFQ,TMT,SILAC: 305
HSPA4, uM: 1
HSPA4, iBAQ: 90
HSPA4, mol/g protein: 3
HSPA5, LFQ,TMT,SILAC: 309
HSPA5, uM: 1
HSPA5, iBAQ: 87
HSPA5, mol/g protein: 3
HSPA8, LFQ,TMT,SILAC: 337
HSPA8, uM: 1
HSPA8, iBAQ: 107
HSPA8, mol/g protein: 3
HSPD1, LFQ,TMT,SILAC: 309
HSPD1, uM: 1
HSPD1, iBAQ: 94
HSPD1, mol/g protein: 3
HSPH1, LFQ,TMT,SILAC: 309
HSPH1, uM: 1
HSPH1, iBAQ: 89
HSPH1, mol/g protein: 3
IDH1, LFQ,TMT,SILAC: 293
IDH1, uM: 1
IDH1, iBAQ: 88
IDH1, mol/g protein: 5
LMNA, LFQ,TMT,SILAC: 311
LMNA, uM: 1
LMNA, iBAQ: 89
LMNA, mol/g protein: 3
MAPK1, LFQ,TMT,SILAC: 309
MAPK1, uM: 1
MAPK1, iBAQ: 95
MAPK1, mol/g protein: 3
MARCKS, LFQ,TMT,SILAC: 305
MARCKS, uM: 1
MARCKS, iBAQ: 96
MARCKS, mol/g protein: 3
MDH2, LFQ,TMT,SILAC: 309
MDH2, uM: 1
MDH2, iBAQ: 94
MDH2, mol/g protein: 3
MYH9, LFQ,TMT,SILAC: 305
MYH9, uM: 1
MYH9, iBAQ: 104
MYH9, mol/g protein: 3
NCL, LFQ,TMT,SILAC: 323
NCL, uM: 1

NCL, iBAQ: 106
NCL, mol/g protein: 3
NONO, LFQ,TMT,SILAC: 325
NONO, uM: 1
NONO, iBAQ: 83
NONO, mol/g protein: 3
OTUB1, LFQ,TMT,SILAC: 306
OTUB1, uM: 1
OTUB1, iBAQ: 85
OTUB1, mol/g protein: 3
PDIA3, LFQ,TMT,SILAC: 319
PDIA3, uM: 1
PDIA3, iBAQ: 96
PDIA3, mol/g protein: 3
PGD, LFQ,TMT,SILAC: 305
PGD, uM: 1
PGD, iBAQ: 85
PGD, mol/g protein: 3
PGK1, LFQ,TMT,SILAC: 317
PGK1, uM: 1
PGK1, iBAQ: 94
PGK1, mol/g protein: 3
PHB, LFQ,TMT,SILAC: 305
PHB, uM: 1
PHB, iBAQ: 84
PHB, mol/g protein: 3
PHGDH, LFQ,TMT,SILAC: 305
PHGDH, uM: 1
PHGDH, iBAQ: 83
PHGDH, mol/g protein: 3
PLEC, LFQ,TMT,SILAC: 357
PLEC, uM: 1
PLEC, iBAQ: 108
PLEC, mol/g protein: 3
PRDX2, LFQ,TMT,SILAC: 305
PRDX2, uM: 1
PRDX2, iBAQ: 98
PRDX2, mol/g protein: 3
PURA, LFQ,TMT,SILAC: 305
PURA, uM: 1
PURA, iBAQ: 90
PURA, mol/g protein: 3
RPL18, LFQ,TMT,SILAC: 301
RPL18, uM: 1
RPL18, iBAQ: 104
RPL18, mol/g protein: 3
RPS13, LFQ,TMT,SILAC: 305

RPS13, uM: 1
RPS13, iBAQ: 91
RPS13, mol/g protein: 3
RPS7, LFQ,TMT,SILAC: 301
RPS7, uM: 1
RPS7, iBAQ: 82
RPS7, mol/g protein: 3
SERBP1, LFQ,TMT,SILAC: 334
SERBP1, uM: 2
SERBP1, iBAQ: 88
SERBP1, mol/g protein: 9
SLC25A4, LFQ,TMT,SILAC: 309
SLC25A4, uM: 1
SLC25A4, iBAQ: 107
SLC25A4, mol/g protein: 3
SLC25A5, LFQ,TMT,SILAC: 309
SLC25A5, uM: 1
SLC25A5, iBAQ: 107
SLC25A5, mol/g protein: 3
SLC3A2, LFQ,TMT,SILAC: 331
SLC3A2, uM: 2
SLC3A2, iBAQ: 99
SLC3A2, mol/g protein: 3
STXBP1, LFQ,TMT,SILAC: 431
STXBP1, uM: 1
STXBP1, iBAQ: 152
STXBP1, mol/g protein: 6
TCP1, LFQ,TMT,SILAC: 315
TCP1, uM: 1
TCP1, iBAQ: 83
TCP1, mol/g protein: 3
TPI1, LFQ,TMT,SILAC: 309
TPI1, uM: 1
TPI1, iBAQ: 91
TPI1, mol/g protein: 3
TUBB2A, LFQ,TMT,SILAC: 323
TUBB2A, uM: 1
TUBB2A, iBAQ: 101
TUBB2A, mol/g protein: 3
UBA1, LFQ,TMT,SILAC: 309
UBA1, uM: 1
UBA1, iBAQ: 91
UBA1, mol/g protein: 3
VIM, LFQ,TMT,SILAC: 330
VIM, uM: 1
VIM, iBAQ: 93
VIM, mol/g protein: 3
YWHAB, LFQ,TMT,SILAC: 316

YWHAB, uM: 1
YWHAB, iBAQ: 103
YWHAB, mol/g protein: 3
YWHAE, LFQ,TMT,SILAC: 351
YWHAE, uM: 1
YWHAE, iBAQ: 99
YWHAE, mol/g protein: 3
YWHAG, LFQ,TMT,SILAC: 309
YWHAG, uM: 1
YWHAG, iBAQ: 92
YWHAG, mol/g protein: 3
YWHAH, LFQ,TMT,SILAC: 309
YWHAH, uM: 1
YWHAH, iBAQ: 89
YWHAH, mol/g protein: 3
YWHAZ, LFQ,TMT,SILAC: 311
YWHAZ, uM: 1
YWHAZ, iBAQ: 96
YWHAZ, mol/g protein: 3

(B) Protein levels after normalization of 74 proteins that are present in the largest number of collected data sets (24 of 25 data sets).
Sample size: 31213
Sample sizes per protein:
ACAT1, processed: 419
ACO2, processed: 405
ADD1, processed: 558
ADD3, processed: 431
ALDOA, processed: 471
ALDOC, processed: 397
ANXA6, processed: 456
ATP5B, processed: 444
BASP1, processed: 414
CAND1, processed: 416
CAP1, processed: 399
CAPZB, processed: 560
CCT2, processed: 391
CCT3, processed: 415
CCT4, processed: 395
CKB, processed: 427
CYB5R3, processed: 391
DPYSL2, processed: 421
EEF1A1, processed: 422
EEF1G, processed: 403
EEF2, processed: 399
EZR, processed: 392
FASN, processed: 400

GAPDH, processed: 497
GDI2, processed: 402
GLUD1, processed: 426
GNB1, processed: 404
GNB2, processed: 388
HK1, processed: 417
HNRNPA2B1, processed: 416
HNRNPK, processed: 512
HSP90AA1, processed: 435
HSP90AB1, processed: 429
HSP90B1, processed: 399
HSPA4, processed: 399
HSPA5, processed: 400
HSPA8, processed: 448
HSPD1, processed: 407
HSPH1, processed: 402
IDH1, processed: 387
LMNA, processed: 404
MAPK1, processed: 408
MARCKS, processed: 405
MDH2, processed: 407
MYH9, processed: 413
NCL, processed: 433
NONO, processed: 412
OTUB1, processed: 395
PDIA3, processed: 419
PGD, processed: 394
PGK1, processed: 415
PHB, processed: 393
PHGDH, processed: 392
PLEC, processed: 469
PRDX2, processed: 407
PURA, processed: 399
RPL18, processed: 409
RPS13, processed: 400
RPS7, processed: 387
SERBP1, processed: 433
SLC25A4, processed: 420
SLC25A5, processed: 420
SLC3A2, processed: 435
STXBP1, processed: 590
TCP1, processed: 402
TPI1, processed: 404
TUBB2A, processed: 428
UBA1, processed: 404
VIM, processed: 427
YWHAB, processed: 423
YWHAE, processed: 454

YWHAG, processed: 405
YWHAH, processed: 402
YWHAZ, processed: 411

(C) Variability scores of 74 proteins that are present in the largest number of collected data sets (24 of 25 data sets).
Sample sizes:
processed to uM: 74
LFQ,TMT,SILAC: 74
iBAQ: 74
mol/g protein: 74
uM: 5 (molar from source)

(D) Histograms of protein levels for the same set of 74 proteins as in (A-C):
sample size is 31213 for each group

(E) Histograms of variability scores for the same set of 74 proteins as in (A-C):
sample size “before” is 375, sample size “after” is 74 (we omitted from visualisation zero variability score corresponding to the zero coefficient of variation of the protein level data for a given type of protein)

Figure 4. Correlation of protein concentrations confirms good agreement of the integrated data.
(A, C, E) Pearson coefficient of correlation for protein concentrations from different studies (A), brain regions (C), cell types (E) after normalization: sample sizes are shown in Supplementary Figure 3 (A,C,E). Precise numeric values can be obtained by running Jupyter Notebook BrainMolecularAtlas/main_6_plots.ipynb.
(B) Representative examples for the comparison of protein concentrations data from two studies: sample size 6261
(D) Representative examples for the comparison of protein concentrations data from two brain regions: sample size 9556
(F) Representative examples for the comparison of protein concentrations data from two cell types: sample size 8738

Figure 5. Statistical evaluation of factors with a potential to explain biological variability of protein concentrations.
(A-C) Comparison of protein concentrations in different groups of proteins:
(A) sample sizes 3624 (OSR) and 2941 (DNA repair)
(B) sample sizes 3624 (OSR) and 1318 (OXPHOS)
(C) sample sizes 2166 (neurons) and 2590 (astrocytes)
(D-R) Statistical analyses in permutations with multiple (1000) resampling with the sample sizes of 100.

Figure 6,7. Functional analysis. Detailed results are available from the Supplementary Table 3.

Figure 8. Case study for differential protein concentrations analysis across brain regions and states. Sample size: 3128 proteins

Figure 9. Sources of variability in metabolite concentrations.
(A) Effects of measurement methods and species on the concentrations of metabolites.
Sample sizes are:
choline, human, mass spectrometry 2
choline, human, mrs 15
choline, mouse, mrs 1
creatine, human, mass spectrometry 2
creatine, human, mrs 15
creatine, mouse, mass spectrometry 4
creatine, mouse, mrs 3
gamma-aminobutyric acid, human, mass spectrometry 2
gamma-aminobutyric acid, mouse, mass spectrometry 13
gamma-aminobutyric acid, mouse, mrs 3
gamma-aminobutyric acid, rat, mass spectrometry 1
gamma-aminobutyric acid, rat, mrs 3
gamma-aminobutyric acid, rat, quantitative autoradiography 4
glutathione, human, mass spectrometry 3
glutathione, mouse, mass spectrometry 4
glutathione, mouse, mrs 2
glutathione, rat, mrs 1
glutathione, review, review 3
glycerophosphocholine, human, mass spectrometry 2
glycerophosphocholine, mouse, mrs 2
glycine, human, mass spectrometry 1
glycine, mouse, mass spectrometry 4
glycine, mouse, mrs 2
glycine, rat, mass spectrometry 10
l-alanine, human, mass spectrometry 2
l-alanine, mouse, mass spectrometry 4
l-alanine, mouse, mrs 2
l-alanine, rat, mass spectrometry 1
l-alanine, rat, mrs 3
l-aspartic acid, human, mass spectrometry 4
l-aspartic acid, mouse, mass spectrometry 4
l-aspartic acid, mouse, mrs 3
l-aspartic acid, rat, mass spectrometry 1
l-aspartic acid, rat, mrs 3
l-aspartic acid, rat, quantitative autoradiography 4
l-glutamic acid, cultured astrocytes, review 5
l-glutamic acid, human, mass spectrometry 3
l-glutamic acid, human, mrs 15
l-glutamic acid, human, review 2
l-glutamic acid, mouse, mass spectrometry 13
l-glutamic acid, mouse, mrs 3
l-glutamic acid, rat, mass spectrometry 10
l-glutamic acid, rat, mrs 3
l-glutamic acid, rat, quantitative autoradiography 4
l-glutamic acid, rat, review 1

l-glutamic acid, review, review 2
l-glutamine, cultured astrocytes, review 3
l-glutamine, human, mass spectrometry 1
l-glutamine, human, mrs 10
l-glutamine, mouse, mrs 3
l-glutamine, rat, mass spectrometry 10
l-glutamine, rat, mrs 3
l-glutamine, rat, quantitative autoradiography 4
l-lactic acid, human, mass spectrometry 3
l-lactic acid, mouse, mass spectrometry 4
l-lactic acid, mouse, mrs 2
l-lactic acid, rat, mrs 3
l-lactic acid, review, review 4
n-acetyl-l-aspartic acid, human, mrs 25
n-acetyl-l-aspartic acid, mouse, mass spectrometry 4
n-acetyl-l-aspartic acid, mouse, mrs 3
n-acetyl-l-aspartic acid, rat, mrs 1
phosphocholine, human, mass spectrometry 2
phosphocholine, mouse, mrs 2
phosphocreatine, human, mass spectrometry 2
phosphocreatine, mouse, mass spectrometry 1
phosphocreatine, mouse, mrs 2
phosphocreatine, rat, chemistry 4
phosphoethanolamine, mouse, mass spectrometry 4
phosphoethanolamine, mouse, mrs 2
succinic acid, human, mass spectrometry 2
succinic acid, mouse, mass spectrometry 4
succinic acid, rat, mrs 2
succinic acid, review, review 1
taurine, human, mass spectrometry 2
taurine, mouse, mass spectrometry 4
taurine, mouse, mrs 3
taurine, rat, mass spectrometry 9

(B) Concentrations of molecules measured by magnetic resonance spectroscopy (MRS, left) and by mass spectrometry (MS, right) with the same set of molecules measured by both MRS and MS. Sample sizes are:

choline, human, mrs 15
choline, mouse, mrs 1
creatine, human, mrs 15
creatine, mouse, mrs 3
gamma-aminobutyric acid, mouse, mrs 3
gamma-aminobutyric acid, rat, mrs 3
glutathione, mouse, mrs 2
glutathione, rat, mrs 1
glycerophosphocholine, mouse, mrs 2

glycine, mouse, mrs 2
l-alanine, mouse, mrs 2
l-alanine, rat, mrs 3
l-aspartic acid, mouse, mrs 3
l-aspartic acid, rat, mrs 3
l-glutamic acid, human, mrs 15
l-glutamic acid, mouse, mrs 3
l-glutamic acid, rat, mrs 3
l-glutamine, human, mrs 10
l-glutamine, mouse, mrs 3
l-glutamine, rat, mrs 3
l-lactic acid, mouse, mrs 2
l-lactic acid, rat, mrs 3
n-acetyl-l-aspartic acid, human, mrs 25
n-acetyl-l-aspartic acid, mouse, mrs 3
n-acetyl-l-aspartic acid, rat, mrs 1
phosphocholine, mouse, mrs 2
phosphocreatine, mouse, mrs 2
phosphoethanolamine, mouse, mrs 2
succinic acid, rat, mrs 2
taurine, mouse, mrs 3

choline, human, mass spectrometry 2
creatine, human, mass spectrometry 2
creatine, mouse, mass spectrometry 4
gamma-aminobutyric acid, human, mass spectrometry 2
gamma-aminobutyric acid, mouse, mass spectrometry 13
gamma-aminobutyric acid, rat, mass spectrometry 1
glutathione, human, mass spectrometry 3
glutathione, mouse, mass spectrometry 4
glycerophosphocholine, human, mass spectrometry 2
glycine, human, mass spectrometry 1
glycine, mouse, mass spectrometry 4
glycine, rat, mass spectrometry 10
l-alanine, human, mass spectrometry 2
l-alanine, mouse, mass spectrometry 4
l-alanine, rat, mass spectrometry 1
l-aspartic acid, human, mass spectrometry 4
l-aspartic acid, mouse, mass spectrometry 4
l-aspartic acid, rat, mass spectrometry 1
l-glutamic acid, human, mass spectrometry 3
l-glutamic acid, mouse, mass spectrometry 13
l-glutamic acid, rat, mass spectrometry 10
l-glutamine, human, mass spectrometry 1
l-glutamine, rat, mass spectrometry 10
l-lactic acid, human, mass spectrometry 3
l-lactic acid, mouse, mass spectrometry 4
n-acetyl-l-aspartic acid, mouse, mass spectrometry 4

phosphocholine, human, mass spectrometry 2
phosphocreatine, human, mass spectrometry 2
phosphocreatine, mouse, mass spectrometry 1
phosphoethanolamine, mouse, mass spectrometry 4
succinic acid, human, mass spectrometry 2
succinic acid, mouse, mass spectrometry 4
taurine, human, mass spectrometry 2
taurine, mouse, mass spectrometry 4
taurine, rat, mass spectrometry 9

5 Supplementary Presentation on the Methods Walkthrough

Proteins.

Data mining.

Step 1: collect protein data.
Corresponding code is in the MADIP/step_1_collect_protein_data.ipynb which calls functions from MADIP/collect_protein_data.py

This module loads publicly available data from the referred sources provided in the manuscript A Standardized Brain Molecular Atlas: a resource for systems modeling and simulation.

First, at this step we read the data (xlsx and txt format) from the supplementary materials of referenced sources. There are individual functions in MADIP/collect_protein_data.py to read the data from different sources.

From each table we keep only the data and metadata, which we consider necessary for further use. We adjust column names and metadata terms to be consistent across the sources. Data that we keep includes 'gene_names', 'Uniprot', 'Study', 'Organism', 'location', 'Age_cat', 'Age_days', 'condition', 'sample_id', 'molecular_weight_kDa', 'raw_data', 'raw_data_units'. Additional columns 'gene_name_unified' and 'Uniprot_unified' are empty at this point.

Additionally, we calculated the approximate age in days, but it was not involved in the downstream analyses. Qualitative age category is defined based on the original data sources.

We removed empty entries and entries with zero value in the raw data, which resulted in the decrease in the number of entries from 2,559,290 to 2,142,774.
Nomenclature alignment.
Step 2: protein nomenclature alignment
Corresponding code is in the MADIP/step_2_protein_ids_alignment.ipynb MADIP/protein_ids_alignment_helpers.py and partially MADIP/step_3_protein_conc_calc_tpaPerSample.ipynb

This module provides helper functions for performing alignment of protein and gene ids among different data sources. There are several filtering procedures that are also performed at this step as described below.

First, we load the data frame produced at step 1 in the Jupyter notebook MADIP/step_1_collect_protein_data.ipynb.

We check for the UniProt protein ids which are potentially coming from the experiment contaminants (“CON_” in the beginning of the protein id is an indicator of the potentially contaminant protein according to the MaxQuant annotation). We dropped those 490 entries from the data frame, so the remaining entries count became 2,142,284.

Next, we manually get additional Uniprot data from https://www.uniprot.org (latest data retrieval was done on 21 July 2020) by the following queries:

1) (taxonomy:"Mus musculus (Mouse) [10090]" OR taxonomy:"Rattus norvegicus (Rat) [10116]" OR taxonomy:"Homo sapiens (Human) [9606]") AND reviewed:yes
2) (taxonomy:"Mus musculus (Mouse) [10090]" OR taxonomy:"Rattus norvegicus (Rat) [10116]" OR taxonomy:"Homo sapiens (Human) [9606]") AND reviewed:no

This data will be needed in the later stages of the nomenclature alignment. Queried data was read and formatted by function process_uniprot_mapping_data() from MADIP/protein_ids_alignment_helpers.py (called from the notebook MADIP/step_2_protein_ids_alignment.ipynb)

Next, we check for the gene names consistency **within** studies using function check_GN_consistency_within_studies(df_all) from MADIP/protein_ids_alignment_helpers.py (called from the notebook MADIP/step_2_protein_ids_alignment.ipynb). It allows us to get the gene names repeated in different entries of the same source in combination with other gene names (this is the case due to the protein annotation and identification). Example: UCHL3;UCHL4 + UCHL3.

Next, we decided to remove partial duplicates of type "GN1;GN2" and "GN1" within studies, because we need unique, unambiguous data. This resulted in the decrease from 2,142,284 to 2,140,373 entries.

Next, we addressed the UniProt annotation of the entries. We cropped the isoform index, i.e. transformed some identifiers from “PPPPPP-Number” to “PPPPPP” format, because we considered the data on isoforms of the protein combined together.

Next we obtained the list of unique sets of UniProt identifiers, which we used later to build a graph of related (“synonymous”) UniProt identifiers.

At the next step we have built two graphs of potential synonyms, one for the gene names and the other one for the UniProt protein identifiers, as described in the Methods part of the main text. We counted occurrences of every gene name and UniProt identifier and the most frequent gene name and UniProt identifier in every connected component of the graph as a main identifier across all identifiers of that component (i.e. potentially synonymous identifiers). This produced two mapping dictionaries. Next we performed many manual check for the key-value correspondence in those dictionaries. A few nomenclature conflicts found by manual checks were resolved with the use of the Mouse Genome Database and UniProt, the most common gene names were kept as final identifiers in the integrated Molecular Atlas.

So the same procedure constructing and using the graph of related identifiers has been completed for gene names and UniProt identifiers, which resulted in mapping dictionaries for those two types of identifiers. In each of these two dictionaries, the list of identifiers corresponds to an identifier which is found the most frequently in the data.

Next, we obtained additional data (MOUSE_10090_idmapping.dat.gz, RAT_10116_idmapping.dat.gz, HUMAN_9606_idmapping.dat.gz) from ftp://ftp.uniprot.org/pub/databases/uniprot/current_release/knowledgebase/idmapping/ (latest download has been done on 05 June 2020) to have a mapping between UniProt identifiers and Gene Names.

Then these mapping dictionaries are applied along with the series of manual checks for the consistency of identifiers as can be seen in Jupyter Notebook MADIP/step_2_protein_ids_alignment.ipynb

Some additional checks and refinements are continued in MADIP/step_3_protein_conc_calc_tpaPerSample.ipynb

Concentration estimations.
Step 3: calculation of protein concentrations
Corresponding code is in the MADIP/step_3_protein_conc_calc_tpaPerSample.ipynb

We start MADIP/step_3_protein_conc_calc_tpaPerSample.ipynb with additional refinements of protein IDs as shown in the Jupyter Notebook.

Next we need to retrieve protein sequences from the UniProt database.
Then we use protein sequences to calculate the theoretical number of peptides (where length of the peptide is considered to be from 6 to 29 amino acids), which can be counted by splitting the protein sequence by particular amino acids, by which enzymes would cut the protein sequence in the experiments. We use information on enzymes reported in the original data sources.

We also obtain the molecular weights of proteins from UniProt where it is not given in the data source.

The we apply the total protein mass approach (Wiśniewski et al. 2014) with some adjustments depending of the experimental data type as shown in the MADIP/step_3_protein_conc_calc_tpaPerSample.ipynb

Other transformations are used in the same Jupyter Notebook for the data reported in mol/g protein and mol/(g total protein).

Step 4: clean metadata
Corresponding code is in the MADIP/step_4_cleanMetadata.ipynb

This code is dedicated to the alignment of age categories for the data coming from the different sources. When the data sources explicitly state the qualitative age categories, these data were used. Otherwise the estimate was made based on days/week reported in the sources, with the use of https://www.jax.org/news-and-insights/jax-blog/2017/november/when-are-mice-considered-old and https://www.ncbi.nlm.nih.gov/pmc/articles/PMC3733029/.

The age categories were: 'embrionic' (includes cultured cells, not to be included in the analyses with the objective for identifying age/developmental-stage specific differences), 'adult', 'mature adult', 'middle-aged', 'post-mortem', 'old', 'young', 'child', 'adolescence' (where 'child','adolescence' and 'post-mortem' categories were only used for human samples).

Step 5: normalisation
Corresponding code is in the MADIP/step_5_normaliz.ipynb

First we introduce combined compound_gene_protein_id, row_id, row_gene_id to better group entries in the data.

Then we perform additional exploratory analysis on the data.

Then we select housekeeping proteins and use data on those proteins to perform median normalization.

Evaluation
Step 6: Comparison of estimated protein concentrations to literature
Corresponding code is in the BrainMolecularAtlas/main_6_plots.ipynb
BrainMolecularAtlas/main_14_absValValid.ipynb

It performs basic operations of data frames.

Step 7: Comparison with PubMed mentions
Corresponding code is in the BrainMolecularAtlas/main_figS4_basedOn_pipeline_concHitsAn_2_14july2020.R

It performs selection of proteins to be analysed and queries generation. Then EUtilsSummary from the RISmed library was used to perform queries, but this small and easy to reproduce part of the code is omitted to allow for a more permissive license for the entire project. Next the background concentration and log fold change are calculated. The figures-generating code for this particular case is also not available due to conflicting license types, but it can easily be reproduced with the ggplot R library.

Step 8: Functional network analysis
This step is done using Cytoscape as follows:

- open Cytoscape
- load the data (we selected proteins in every cell type (neurons, astrocytes, microglia, oligodendrocytes) and brain region of interest (cerebellum, cortex, hippocampus, striatum, brainstem, thalamus, amygdala) with concentrations above 99% of overall protein levels across cell types and brain regions, correspondingly)
- click on STRING plugin
- set data as STRING network
- click on clustering
- choose MCL
- set inflation to 5
- run it
- next for every cluster click on STRING: retrieve functional enrichment

color clusters according to obtained annotation, manually summarised from functional enrichment retrieved

Application
Step 9: Flux balance and flux variability analyses
Corresponding code is in the BrainMolecularAtlas/main_10_fba_fig7a.ipynb

This Jupyter Notebook relies on the Cobra package for the flux variability analysis.

Metabolites.

Corresponding code is in the BrainMolecularAtlas/figS7_adj_conc_auto_2_plots.R (for data mining from PubMed) and in the BrainMolecularAtlas/fig10_metabolites.ipynb

The code in BrainMolecularAtlas/figS7_adj_conc_auto_2_plots.R is using the Adjutant R package.

The code in BrainMolecularAtlas/fig10_metabolites.ipynb is based on standard operations with data frames and is using PubChemPy for the alignment of identifiers.

Additional details on the Python environment.
Python version 3.7.4
Python packages (pip freeze, please see the source code to note which packages are used by which script/notebook in particular; to provide the code under more permissive license, we had to remove from the code some non-critical parts which used more restrictive licenses):
altair==4.1.0
anyio==2.2.0
appdirs==1.4.4
argon2-cffi==20.1.0
async-generator==1.10
attrs==20.3.0
Babel==2.9.0
backcall==0.2.0
beautifulsoup4==4.9.3
bioservices==1.7.11
bleach==3.3.0
bluepy==0.14.15
bluepy-configfile==0.1.14
bluepysnap==0.6.1
Bottleneck==1.2.1
cached-property==1.5.1
certifi==2020.12.5
cffi==1.14.5
chardet==4.0.0
Click==7.0
colorama==0.4.4
colorlog==4.8.0
cycler==0.10.0
decorator==4.4.2
defusedxml==0.7.1
easydev==0.11.0
entrypoints==0.3
enum-compat==0.0.3
future==0.17.1
gevent==21.1.2
greenlet==1.0.0
grequests==0.6.0
h5py==2.10.0

idna==2.10
importlib-metadata==3.10.0
ipykernel==5.5.3
ipython==7.22.0
ipython-genutils==0.2.0
jedi==0.18.0
Jinja2==2.11.3
json5==0.9.5
jsonschema==3.2.0
jupyter-client==6.1.12
jupyter-core==4.7.1
jupyter-packaging==0.7.12
jupyter-server==1.5.1
jupyterlab==3.0.12
jupyterlab-pygments==0.1.2
jupyterlab-server==2.4.0
kiwisolver==1.1.0
lazy==1.2
libsonata==0.1.6
lxml==4.4.1
MarkupSafe==1.1.1
matplotlib==3.1.1
mistune==0.8.4
nbclassic==0.2.6
nbclient==0.5.3
nbconvert==6.0.7
nbformat==5.1.3
nest-asyncio==1.5.1
networkx==2.5.1
neurom==1.4.20
notebook==6.3.0
numexpr==2.7.0
numpy==1.17.3
packaging==20.9
pandas==1.0.4
pandocfilters==1.4.3
parso==0.8.2
pathlib2==2.3.5
patsy==0.5.1
pexpect==4.8.0
pickleshare==0.7.5

Pillow==7.0.0
prometheus-client==0.10.0
prompt-toolkit==3.0.18
ptyprocess==0.7.0
PubChemPy==1.0.4
pycparser==2.20
Pygments==2.8.1
pylru==1.2.0
pyparsing==2.4.2
pyrsistent==0.16.0
python-dateutil==2.8.0
pytz==2019.3
PyYAML==5.3.1
pyzmq==22.0.3
requests==2.25.1
requests-cache==0.5.2
scikit-posthocs==0.6.7
scipy==1.3.1
seaborn==0.11.1
Send2Trash==1.5.0
six==1.12.0
sniffio==1.2.0
soupsieve==2.2.1
SQLAlchemy==1.3.9
statsmodels==0.12.2
suds-jurko==0.6
terminado==0.9.4
testpath==0.4.4
toolz==0.11.1
tornado==6.1
tqdm==4.36.1
traitlets==5.0.5
typing-extensions==3.7.4.3
urllib3==1.26.4
wcwidth==0.2.5
webencodings==0.5.1
wrapt==1.12.1
xlrd==1.2.0
XlsxWriter==1.3.8
xmltodict==0.12.0
zipp==3.4.1
zope.event==4.5.0

zope.interface==5.3.0

6 Supplementary Tables and Data Sheets Overview

Table 1. Overview of data sources used in the Brain Molecular Atlas for estimation of the protein and metabolite concentrations (two sheets).

Table 2. Statistics summary and supplemental statistical analysis results (two sheets).

DBM/OVS refers to distance between medians (DBM) as a percentage of overall visible spread (OVS).

Table 3. Results from the metabolites automated PubMed data mining (with corresponding queries).

Data Sheets 1-4. Estimated protein concentrations (split into 4 files due to file size limit in the Journal's editorial management system).

Data Sheet 5. Estimated metabolite concentrations.

Data Sheet 6. Functional enrichment of protein-protein interaction networks related to Figures 6 and 7.

Column "ClusterID" refers to the location (brain region or cell type) and cluster index number.

Data Sheet 7. List of differentially expressed proteins related to Fig 8, Supplementary Figures 6-9. "GroupID" column reports the group name which defines to which part and which figure every entry corresponds.
